# Effects of new hypoglycemic drugs on cardiac remodeling: a systematic review and network meta-analysis

**DOI:** 10.1186/s12872-023-03324-6

**Published:** 2023-06-09

**Authors:** Yi-lin Huang, Xiao-zhuo Xu, Jing Liu, Pin-yao Wang, Xue-li Wang, Hong-lin Feng, Cheng-jiang Liu, Xu Han

**Affiliations:** 1 Department of Geriatrics, Affiliated Hospital of Nanjing University of Chinese Medicine, 155 Hanzhongmen Road, Nanjing, 210001 Jiangsu China; 2 Department of General Medicine, Affiliated Anqing First People’s Hospital of Anhui Medical University, Anqing, Anhui China

**Keywords:** SGLT-2 inhibitors, GLP-1 agonists, DPP-4 inhibitors, Cardiac remodeling

## Abstract

**Background:**

In recent years, the incidence of diabetes mellitus has been increasing annually, and cardiovascular complications secondary to diabetes mellitus have become the leading cause of death in diabetic patients. Considering the high incidence of type 2 diabetes (T2DM) combined with cardiovascular disease (CVD), some new hypoglycemic agents with cardiovascular protective effects have attracted extensive attention. However, the specific role of these regimens in ventricular remodeling remains unknown. The purpose of this network meta-analysis was to compare the effects of sodium glucose cotransporter type 2 inhibitor (SGLT-2i), glucagon-like peptide 1 receptor agonist (GLP-1RA) and dipeptidyl peptidase-4 inhibitor (DPP-4i) on ventricular remodeling in patients with T2DM and/or CVD.

**Methods:**

Articles published prior to 24 August 2022 were retrieved in four electronic databases: the Cochrane Library, Embase, PubMed, and Web of Science. This meta-analysis included randomized controlled trials (RCTs) and a small number of cohort studies. The differences in mean changes of left ventricular ultrasonic parameters between the treatment and control groups were compared.

**Results:**

A total of 31 RCTs and 4 cohort studies involving 4322 patients were analyzed. GLP-1RA was more significantly associated with improvement in left ventricular end-systolic diameter (LVESD) [MD = -0.38 mm, 95% CI (-0.66, -0.10)] and LV mass index (LVMI) [MD = -1.07 g/m^2^, 95% CI (-1.71, -0.42)], but significantly decreased e' [MD = -0.43 cm/s 95% CI (-0.81, -0.04)]. DPP-4i was more strongly associated with improvement in e' [MD = 3.82 cm/s, 95% CI (2.92,4.7)] and E/e'[MD = -5.97 95% CI (-10.35, -1.59)], but significantly inhibited LV ejection fraction (LVEF) [MD = -0.89% 95% CI (-1.76, -0.03)]. SGLT-2i significantly improved LVMI [MD = -0.28 g/m^2^, 95% CI (-0.43, -0.12)] and LV end-diastolic diameter (LVEDD) [MD = -0.72 ml, 95% CI (-1.30, -0.14)] in the overall population, as well as E/e' and SBP in T2DM patients combined with CVD, without showing any negative effect on left ventricular function.

**Conclusion:**

The results of the network meta-analysis provided high certainty to suggest that SGLT-2i may be more effective in cardiac remodeling compared to GLP-1RA and DPP-4i. While GLP-1RA and DPP-4i may have a tendency to improve cardiac systolic and diastolic function respectively. SGLT-2i is the most recommended drug for reversing ventricular remodeling in this meta-analysis.

**Supplementary Information:**

The online version contains supplementary material available at 10.1186/s12872-023-03324-6.

## Introduction

Diabetes is a chronic metabolic disorder whose prevalence is increasing annually. The total number of people with diabetes in the world is expected to increase to nearly 780 million by 2045, with type 2 diabetes mellitus (T2DM) accounting for 90% of the total [1, 2]. Long-term persistent chronic hyperglycemia will lead to destructive macrovascular, microvascular lesions and other complications. Among them, cardiovascular disease (CVD) is the main clinical risk factor for death in patients with diabetes [3, 4]. Epidemiological studies have found that T2DM carries a two to six times risk of death from cardiovascular etiologies than people without T2DM [5].

In the traditional treatment for T2DM, metformin is usually used as the first-line drug, and sulfonylureas and thiazolidinediones can be added on top of it [6]. However, the effects of these drugs on cardiovascular system are not clear. Studies showed that the addition of rosiglitazone to the hypoglycemic treatment for T2DM increases the risk of heart failure and certain fractures [7, 8]. Considering the high incidence of T2DM combined with CVD, some new hypoglycemic agents with cardiovascular protective effects have attracted extensive attention. The sodium glucose cotransporter 2 (SGLT-2i), such as dapagliflozin, canagliflozin, and empagliflozin, have demonstrated good cardiovascular safety in large-scale experimental studies on cardiovascular outcomes, especially in reducing the risk of heart failure [9–11]. Glucagon-like peptide 1 receptor agonist (GLP-1RA) has been proved to be beneficial for CVD by both oral administration and subcutaneous injection [12]. Kato et al. found that dipeptidyl peptidase-4 inhibitor (DPP-4i), alogliptin improved coronary flow reserve in patients with T2DM and coronary artery disease [13].

Cardiac remodeling, including changes in ventricular wall thickness, ventricular volume and cardiac mass, is a progressive pathological change in the original substance and morphology of the ventricle. Although the development of ventricular remodeling and the prognosis of heart failure is consistent [14, 15], the process of ventricular remodeling is more prolonged and complex [16]. Previous studies have found that in patients with heart failure, SGLT-2i can significantly improve the volume, mass, and ventricular systolic function in the left ventricular [17]. In contrast, the REFORM trial failed to demonstrate any effect of SGLT-2i on cardiac remodeling in patients with heart failure and T2DM [18]. Sardu et al*.* observed that in T2DM patients with heart failure, GLP-1RA significantly improved left ventricular ejection fraction (LVEF) and 6-min walking test (6MWT) [19]. However, Kumarathurai et al*.* proved that GLP-1RA had no statistically significant benefit for systolic function [20].

Given the inconsistent results of existing studies, more data are needed to analyze and compare the efficacy of different treatments. However, there is a lack of head-to-head clinical trials. Network meta-analysis can combine direct and indirect comparisons to help investigators analyze the efficacy of different regimens. Therefore, this study comprehensively evaluated the effects of SGLT-2i, DPP-4i, and GLP-1RA on cardiac remodeling through network meta-analysis, in order to explore the agents that have the best efficacy for reversing ventricular remodeling in patients with T2DM and/or CVD.

This study has several strengths. First, this meta-analysis updated results from clinical studies over the past two years, thus several recently published, large-scale and high-quality RCTs have been included. Second, in order to increase the credibility of the study, our study excluded clinical experiments with the number of participants in each group being less than 20. Finally, this article is the first study including the effects of SGLT-2i, GLP-1RA and DPP-4i on systolic blood pressure (SBP), immunoreactive amino-terminal pro-brain natriuretic peptide (NT-proBNP) and 6-min walk test (6MWT), of which 6MWT is a strong independent predictor of mortality in outpatients with heart failure [21].

## Methods

### Eligibility standards

This meta-analysis was carried out in accordance with the guidelines on preferred reporting elements for systematic reviews and meta-analyses (PIRISMA). The study protocol was registered in PROSPERO, an international prospective register of systematic reviews (registration code CRD42022365986).

The studies were included while they reached the specific criteria for this review were:

- population: patients 18 years of age or over who have been diagnosed with T2DM with or without CVD, or patients aged 18 years or older with CVD alone;
- intervention: comparison between a GLP-1RA, a DPP-4i, or a SGLT-2i and an active control. The experimental group treated with placebo or one of the three drugs can be used as a control group, other hypoglycemic drugs are not included.
- outcome: report at least one outcome variable evaluated by echocardiography or cardiovascular magnetic resonance (CMR). The main outcomes were changes in LV remodeling parameters, including systolic and diastolic function, mass, and volume.
- study design: studies were RCT with parallel or cross-group designs or cohort studies.

### Search strategies

Computer searches were carried out on the databases of the Cochrane Library, Embase, PubMed, and Web of Science from its inception to 24 August, 2022. These terms were used in the research: ("cardiac reverse remodeling" OR "left ventricular dysfunction") AND ("sodium glucose transporter 2 inhibitors" OR "glucagon like peptide 1 agonists" OR "dipeptidyl peptidase 4 inhibitors") AND ("randomized controlled trial" OR "controlled clinical trial") (Additional file 1).

### Study selection

The EndNote X9 software [22] was used to exclude duplicates and documents that do not meet the inclusion criteria. Unique studies were again cross-checked manually (LJ, WXL and LCJ). Two investigators (XXZ and WPY) screened the literature for compliance by reviewing the titles and abstracts, read the complete texts, and extracted the data from the selected studies separately. Any discrepancies will be resolved by consensus and, if necessary, a third examiner will be consulted for arbitration. The reasons for inclusion or exclusion are well documented. Case reports, letters, records of meetings were excluded. The process of the study is documented and summarized by the PRISMA flow chart.

### Data extraction

Two investigators (LJ and WPY) used predefined data tables to record data for each item included in the literature independently. For example, authors, population, year of release, gender ratio, subject ages, study design, sample size, intervention, grouping and the number of people in the group, baseline and endpoint data, including counts and effect estimates (mean ± SD), country, follow-up months, title, and conclusion. Data was independently examined for accuracy.

### Definition of outcomes

This meta-analysis was assessed on the difference in average variation in echocardiographic endpoints between treatment groups and controls. The echocardiographic parameters included LVEF, LV end-diastolic diameter (LVEDD), LV end-systolic diameter (LVESD), LV end-diastolic volume (LVEDV), LV end-systolic volume (LVESV), LV mass index (LVMI), early diastolic velocity (e′), early and mitral inflow E velocity to tissue Doppler e′ ratio (E/e′), diastolic to late diastolic velocities ratio (E/A). The results of systolic blood pressure (SBP), immunoreactive amino-terminal pro-brain natriuretic peptide (NT-proBNP) and 6MWT were also counted in this study as a reference for evaluating the effect of drugs on improving left ventricular function.

### Assessment of risk of bias

In accordance with the risk of bias tool (Risk of Bias) of the Review Manager 5.3 software [23], Two evaluators (XXZ and LJ) conducted a quality assessment of the study, with the following main evaluations: Blinding of subjects and participants, allocation concealment, random sequence generation, blinding of outcome assessments, selective reporting, incomplete outcome data, and other biases. The quality of the literature was assessed on three grades: "unclear" (lack of relevant information or uncertain bias), "high" (high bias) and "low" (low bias). The different evaluation levels of the first two will be resolved by discussion with a third researcher. Finally, the results of the risk bias assessment were visualized by the software mentioned above.

### Statistical analysis

A network meta-analysis was used to estimate comparative effects by combining the direct and indirect evidence provided by the selected treatment options. To visualize the geometry of the network and the nodal connections, we have represented the geometry of the network of proofs with a network diagram. The heterogeneity tests were mainly determined according to I^2^. If heterogeneity did not exist between study results (I^2^ ≤ 50%), the present study used a fixed effect model for the meta-analysis. If there was heterogeneity between study outcomes (I^2^ > 50%), the source of heterogeneity was further analyzed. After excluding effects due to significant clinical heterogeneity, a randomized effects model was used for the meta-analysis. A network meta-analysis was performed using STATA 16.0 software [24] under a frequency-based random effects model, in which study outcome indicators were networked by grouping instructions. Additionally, data processing, network data plots, funnel plots, forest plots and area under curve ranking (SUCRA) were performed sequentially. The strengths and weaknesses of the interventions were ranked according to the magnitude of SUCRA. SUCRA = 1 means that the treatment was completely effective, and SUCRA = 0 indicates that the treatment was completely ineffective.

## Results

### Study selection

**Fig. 1:**
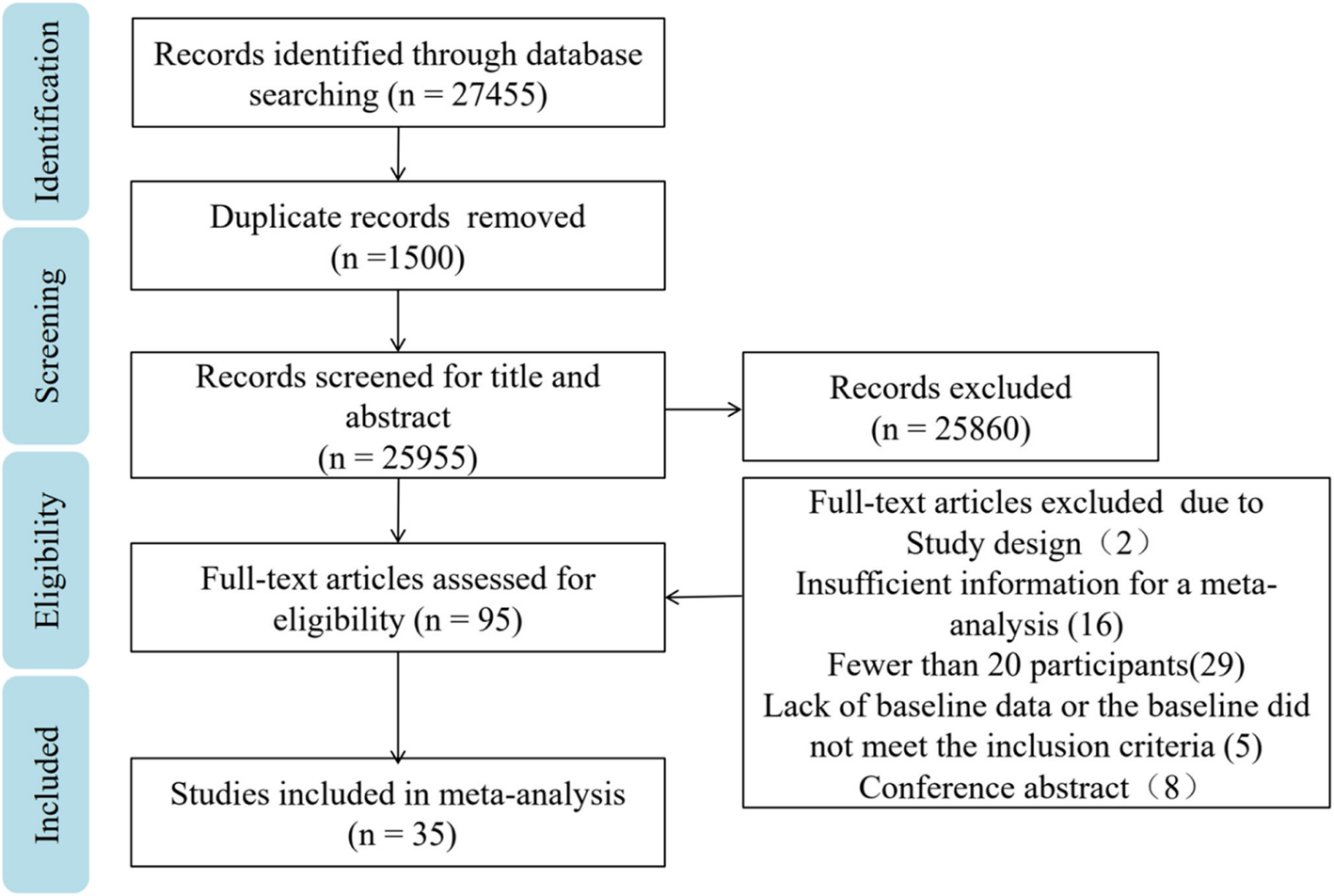
Summary of study identification and selection

After an initial search, 27455 articles were downloaded from 4 databases. Then after reading the titles and abstracts, 95 articles were obtained by excluding articles with duplicate content and those not relevant to this study. After reading the full text in detail, 60 articles were excluded for the following reasons: the study design (*n* = 2), insufficient information for a meta-analysis (*n* = 16), fewer than 20 participants (*n* = 29), lack of baseline data or the baseline did not meet the inclusion criteria (*n* = 5), conference abstract (*n* = 8). Thirty-five studies were subsequently included in this network meta-analysis (Fig. 1).

### Study characteristics

**Fig. 2:**
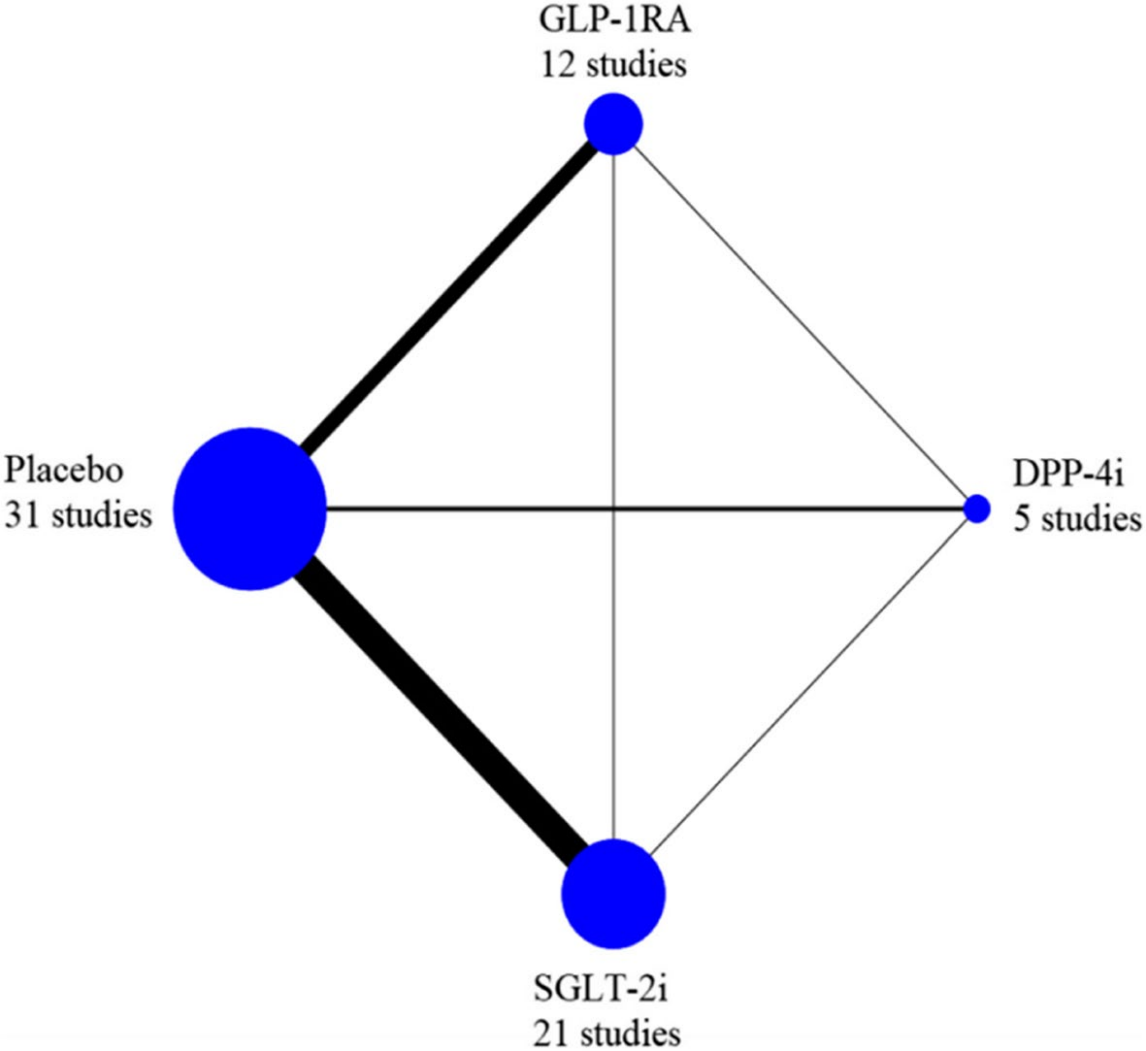
Network plot for all studies. Note: DPP-4i: dipeptidyl peptidase-4 inhibitor; GLP-1RA: glucagon-like peptide-1 receptor agonist; SGLT-2i: sodium glucose cotransporter type 2 inhibitor

In this network meta-analysis, 35 studies were included, comprising a total sample size of 4322 participants. The 35 studies included 31 RCTs and 4 cohort studies. The summary data of each included study are shown in Additional file 2: Table S1, and the network plot is shown in Fig. 2.

### Risk of bias within studies

Of all studies, six were designed as open-label [25–30], and two of them were judged to be at high risk of selective reporting bias. One article did not specify the method of randomization [29], and the other article reported more than 20% of total withdrawals during follow-up, but did not mention the specific reasons [27]. Four studies were cohort studies and were rated high quality according to NOS scores. The details of risk of bias quality assessment for each RCT and cohort study is shown in Additional file 3: Figure S1-2, Table S2.

### Synthesis of results

#### LVEF

1. Evidence Network. Twenty-eight literatures reported LVEF involved three antidiabetic regimens. The size of the sample containing the intervention determines the size of the points, and the number of RCTs of the treatment intervention determines the thickness of the line. There is four closed-loop formation (Additional file 4: Figure S3(a)).
2. Network Meta-analysis. Compared with placebo, MD and 95% CI of DPP-4i was -0.89% and (-1.76, -0.03). No difference was found in the pairwise comparison of treatment effects between the two drugs. The specific results are shown in Table 1 and Additional file 6: FigureS5(a).

**Table 1:** Matrix of pairwise comparisons of regimens on change of LVEF% (shown as standard mean difference and 95% confidence intervals)

	B	C	D	A
SCURA(%)				
B	0	-0.32 (-0.77,0.13)	-0.45 (-1.95,1.04)	0.20 (-1.75,2.14)
C Placebo	0.32 (-0.13,0.77)	0	-0.13 (-1.55,1.30)	-0.65 (-1.36,0.06)
D	0.45 (-1.04,1.95)	0.13 (-1.30,1.55)	0	-0.32 (-0.87,0.22)
A	-0.20 (-2.14,1.75)	0.65 (-0.06,1.36)	0.32 (-0.22,0.87)	0

*Abbre*: A DDP-4i, B GLP-1RA, C Placebo, D SGLT-2i

#### Results of subgroup analyses

To identify patients who may benefit more from antidiabetes therapy, patients were divided into two subgroups according to whether they had CVD with T2DM or CVD alone. Treatment with GLP-1RA significantly improved the LVEF in patients with CVD alone [MD = 1.65%, 95% CI (0.49, 2.81)], while no significant difference was identified between 3 interventions and placebo among patients with CVD and T2DM (Additional file 9: TableS3(a),(b)).

#### LVEDD

1. Evidence Network. Ten literatures reported LVEDD involved three antidiabetic regimens. All 3 cis indicate direct comparisons and did not form a closed loop (Additional file 4: Figure S3(b)).
2. Network Meta-analysis. The difference in mean pre- and post-treatment change in SGLT-2i for LVEDD compared to placebo was greater than zero [MD -0.72 mm 95% CI (-1.30, -0.14)], suggesting that SGLT-2i was more associated with improvement in LVEDD than placebo (Table 2 and Additional file 6: FigureS5(b)).

**Table 2:** Matrix of pairwise comparisons of regimens on change of LVEDD (shown as standard mean difference and 95% confidence intervals)

	D	B	A	C
SCURA(%)				
D	0	0.66 (-0.44,1.76)	0.66 (-0.44,1.77)	0.72 (0.14,1.30)
B	-0.66 (-1.76,0.44)	0	0.01 (-1.32,1.33)	0.06 (-0.87,1.00)
A	-0.66 (-1.77,0.44)	-0.01 (-1.33,1.32)	0	0.06 (-0.88,1.00)
C Placebo	-0.72 (-1.30,-0.14)	-0.06 (-1.00,0.87)	-0.06 (-1.00,0.88)	0

*Abbre:* A DDP-4i, B GLP-1RA, C Placebo, D SGLT-2i

#### LVEDV

1. Evidence Network. Eighteen literatures reported LVEDV involved three antidiabetic regimens. All 3 cis indicate direct comparisons and did not form a closed loop (Additional file 4: Figure S3(b)).
2. Network Meta-analysis. No significant differences emerged between the four interventions (Table 3 and Additional file 6: FigureS5(c)).

**Table 3:** Matrix of pairwise comparisons of regimens on change of LVEDV (shown as standard mean difference and 95% confidence intervals)

	B	C	A	D
SCURA(%)				
B	0	0.18 (-0.93,1.30)	0.34 (-2.75,3.43)	0.46 (-1.00,1.93)
C Placebo	-0.18 (-1.30,0.93)	0	0.16 (-2.73,3.04)	0.28 (-0.67,1.22)
A	-0.34 (-3.43,2.75)	-0.16 (-3.04,2.73)	0	0.12 (-2.91,3.15)
D	-0.46 (-1.93,1.00)	-0.28 (-1.22,0.67)	-0.12 (-3.15,2.91)	0

*Abbre*: A DDP-4i, B GLP-1RA, C Placebo, D SGLT-2i

#### LVESD

1. Evidence Network. LVESD was reported in 6 studies, involving 2 antidiabetic therapies (GLP-1RA and SGLT-2i). All interventions represent direct comparison without closed-loop formation (Additional file: 4 Figure S3(d)).
2. Network Meta-analysis. GLP-1RA significantly reduced LVESD compared with placebo [MD = -0.38 mm, 95% CI (-0.66, -0.10)]. There was no difference in the pairwise comparison of treatment effects between the two drugs (Table 4 and Additional file 6: FigureS5(d)).

**Table 4:** Matrix of pairwise comparisons of regimens on change of LVESD (shown as standard mean difference and 95% confidence intervals)

	A	B	C
SCURA(%)			
A	0	0.38 (0.10,0.66)	0.16 (-0.24,0.57)
B Placebo	-0.38 (-0.66,-0.10)	0	-0.22 (-0.51,0.08)
C	-0.16 (-0.57,0.24)	0.22 (-0.08,0.51)	0

*Abbre*: A GLP-1RA, B Placebo, C SGLT-2i

#### LVESV

1. Evidence Network. Sixteen pieces of literature reported LVESV involving 3 interventions. All interventions represent direct comparison without closed-loop formation (Additional file 4: Figure S3(e)).
2. Network Meta-analysis. No significant difference was identified in LVESV between the 3 interventions (Table 5 and Additional file 6: FigureS5(e)).

**Table 5:** Matrix of pairwise comparisons of regimens on change of LVESV (shown as standard mean difference and 95% confidence intervals)

	A	C	B
SCURA(%)			
A	0	0.03 (-0.45,0.50)	0.31 (-0.03,0.65)
C	-0.03 (-0.50,0.45)	0	0.28 (-0.05,0.61)
B Placebo	-0.31 (-0.65,0.03)	-0.28 (-0.61,0.05)	0

*Abbre*: A GLP-1RA, B Placebo, C SGLT-2i

#### LVMI

1. Evidence Network. Ten literatures reported LVMI involved three antidiabetic regimens. There is one closed-loop formation (Additional file 4: Figure S3(f)).
2. Network Meta-analysis. In terms of the outcome of LVMI, two classes of drug showed significant benefits with regard to reducing LVMI in all patients compared to placebo: GLP-1RA [MD = -1.07 g/m^2^, 95% CI (-1.71, -0.42)], SGLT-2i [MD = -0.28 g/m^2^, 95% CI (-0.43, -0.12)]. In the pairwise comparison, GLP-1RA showed efficacy compared to SGLT-2i [MD = -0.79 g/m^2^, 95% CI (-1.46, -0.12)]. In addition, DPP-4i showed a negative impact compared to SGLT-2i [MD = 1.34 g/m^2^, 95% CI (0.93, 1.75)] (Table 6 and Additional file 6: FigureS5(f)).

**Table 6:** Matrix of pairwise comparisons of regimens on change of LVMI (shown as standard mean difference and 95% confidence intervals)

	A	B	D	C
SCURA(%)				
A	0	0.27 (-0.23,0.78)	1.34 (0.93,1.75)	-0.51 (-1.38,0.35)
B	-0.27 (-0.78,0.23)	0	0.79 (0.12,1.46)	1.07 (0.42,1.71)
D	-1.34 (-1.75,-0.93)	-0.79 (-1.46,-0.12)	0	0.28 (0.12,0.43)
C Placebo	0.51 (-0.35,1.38)	-1.07 (-1.71,-0.42)	-0.28 (-0.43,-0.12)	0

*Abbre:* A DDP-4i, B GLP-1RA, C Placebo, D SGLT-2i

#### e’

1. Evidence Network. e’ was reported in 6 studies, involving 3 antidiabetic therapies. There is no closed-loop formation (Additional file 4: Figure S3(g)).
2. Network Meta-analysis. The results showed that DPP-4i was the only drug that significantly increased e' [MD = 3.82 cm/s, 95% CI (2.92, 4.7)]. Also compared to placebo, the difference in mean change in e’ with GLP-1RA treatment was less than zero [MD = -0.43 cm/s, 95% CI (-0.81, -0.04)], indicating a negative effect of GLP-1RA on e’. In the pairwise comparison, SGLT-2i [MD = -2.94, 95% CI (-4.24, -1.68)] and GLP-1RA [MD = -4.24, 95% CI (-5.22, -3.27)] significantly reduced e' compared to DPP-4i. While GLP-1RA had a more significant negative effect compared to SGLT-2i [MD = -1.28 95% CI (-2.27, -0.3)] (Additional file 9: TableS3(i) and Additional file 6: FigureS5(g)).

#### E/e’

1. Evidence Network. E/e’ was reported in 15 studies, involving 3 antidiabetic therapies. There is one closed-loop formation (Additional file 4: Figure S3(h)).
2. Network Meta-analysis. Compared with placebo, DPP-4i significantly improved E/e' [MD = -5.97 95%CI (-10.35,-1.59)], while GLP-1RA showed a negative impact compared to DPP-4i [MD = 5.78 95% CI (0.60, 10.95)] (Additional file 9: Table S3(j) and Additional file 6: FigureS5(h)).

#### Results of subgroup analyses

The subgroup of patients with CVD and T2DM included 5 studies. In the analysis, SGLT-2i showed a more significant ability to reduce E/e' than placebo [MD =—0.08; 95% CI (-0.79, -0.06)]. No significant difference was found for the rest of the results (Additional file 9: TableS3(c)).

#### E/A

1. Evidence Network. E/A was reported in 12 studies, involving 4 antidiabetic therapies. There is one closed-loop formation (Additional file 4: Figure S3(i)).
2. Network Meta-analysis. No significant difference was identified in the mean change of E/A between the 4 interventions (Additional file 9: Table S3(k) and Additional file 6: FigureS5(i)).

#### SBP, NT-proBNP and 6MWT

A significant association between GLP-1RA therapy and improvement of 6-min walk distance was found in the overall population compared with placebo [MD = 1.52 m, 95% CI (0.29, 2.76)]. No significant difference was identified in the mean change of SBP and NT-proBNP between the 4 interventions. The detailed results and sub-group analysis are shown in Additional file 10, figures are shown in Additional files 4, 6 and 8: Figure S3-6(j,k,l,q,s,t).

### SUCRA probability ranking

According to the SUCRA results, the ranking of the efficacy of the 3 regimens and placebo is shown in Additional file 7: TableS3 and Additional file 8: FigureS6(a-t). SGLT-2i ranked first in treatment effect on LVEDD, and GLP-1RA ranked first in the treatment effect on LVESD, LVEDV, LVESV, E/e’, NT-pro BNP and 6MWT. DPP-4i ranked first in the treatment effect on LVMI, e', E/A and SBP.

### Publication bias

Funnel plots were used to compare the differences in mean changes in all evaluation metrics between the treatment and placebo groups. Most of the scatter points in all of the funnel plots were located on either side of the vertical line. They were fundamentally symmetric and may have some level of publication bias (Additional file 5: Figure S4(a-t)).

### Inconsistency test

No evidence of statistically significant inconsistency was found for any of the ventricular remodeling parameters (global inconsistency tests *P* > 0.05).

## Discussion

This meta-analysis evaluated the effects of three novel hypoglycemic agents on patients with T2DM and/or CVD, focusing on cardiac remodeling parameters, including cardiac function and structure. The main findings were as follows: GLP-1RA treatments significantly improved LVMI and LVESD, but were strongly associated with a negative effect on e’ compared to placebo. Treatment with DPP-4i significantly improved diastolic function in the general population, including e' and E/e', but significantly inhibited LVEF. SGLT-2i significantly improved LVMI and LVEDD in the overall population, as well as E/e' and SBP in T2DM patients combined with CVD, without showing any negative effect on left ventricular function. Therefore, we recommend SGLT-2i as the best agent for improving ventricular remodeling.

This meta-analysis showed that SGLT-2i has a beneficial effect on LVMI, LVEDD in the overall population and significantly improved E/e' and SBP in patients with T2DM combined with CVD. Compared with the meta-analysis published before [31], this meta-analysis demonstrated the superiority of SGLT-2i in reducing LVMI. This may be attributed to our inclusion of clinical data published after 2019, such as the RCT conducted by Ersbøll et, al [32]. An abnormal increase in LVMI can lead to left ventricular hypertrophy (LVH), which is an important predictor of CVD outcomes and mortality [33]. Recent study suggests that the temporal relationship between T2DM and LVH may be bidirectional [34]. This finding highlights the dual therapeutic implications of SGLT-2i treatment for patients with T2DM, as well as for patients with CVD alone. However, the underlying mechanism of SGLT-2i on LV structure and function remains unclear. Recent studies are focused on the core role of autophagy recovery, which is the key mechanism leading to weakened cardiac remodeling and ultimately beneficial to heart failure [35]. Wang et al*.* found that empagliflozin could alleviate myocardial I/R injury and cardiomyocyte apoptosis by inhibiting PERK/ATF4/Beclin1 signal transduction [36]. Yu et al. found that dapagliflozin directly acts on myocardial cells through NHE1/NCX signaling pathway. High-dose of dapagliflozin pretreatment may limit the activation of NLRP3 inflammasome and mediate its selective autophagy [37]. All these evidence prove the role of SGLT-2i in protecting the heart through the autophagy pathway.

Compared with SGLT-2i, GLP-1RA performed better in the improvement of LVMI and were more closely related to the improvement of left ventricular systolic function. However, GLP-1RA had a significant adverse effect on e' when compared to placebo. Burns et al. showed a significant decrease in e' in 15 subjects who accelerated heart rate by atrial pacing [38], suggesting a possible inverse relationship between heart rate and diastolic function. Previous meta-analysis found that GLP-1RA could increase heart rate [39], which may explain the negative effect of GLP-1RA on e'. Based on our findings, GLP-1RA may be a good choice for the treatment of diabetes combined with CVD, but its use in patients with diastolic dysfunction, especially tachycardia, requires caution.

In contrast to GLP-1RA, DPP-4i reduced e' and E/e', which indicates the improvement of left ventricular diastolic function. This was relevant to the inclusion of subgroup analysis in the PROLOGUE trial [28]. Subgroup analyses of the PROLOGUE trial found that sitagliptin significantly reduced the increase in E/e' relative to conventional treatment alone, changing the prognosis by improving diastolic function, and this effect was independent of the patient's blood glucose and blood pressure levels. Meta-analysis [31] by Zhang et al*.* also included subgroup analysis of PROLOGUE test, but no significant effect of DPP-4i on E/e' or e' was found. The difference in the conclusions may be due to the fact that e' includes cross-sections, longitudinal sections and mean values, while this analysis includes only mean values.

On the other hand, our study found a significant association between DPP-4i and decreased LVEF. In a prospective randomized study published by Hiruma et al*.*, patients' LVEF decreased significantly after 12 weeks of sitagliptin use [40]. Similarly, in animal experiments, Mulvihill EE et al. demonstrated that DPP-4 inhibitors impair cardiac function in rodent models [41]. In three large cardiovascular outcomes trials, TECOS, EXAMINE, and SAVOR-(TIMI)53 also did not find any cardiovascular protective effect of DPP-4i [42–44]. Based on the experimental results mentioned above, DPP-4i may not be recommended as a first-choice agent for delaying cardiac remodeling.

### Limitations

As a meta-analysis of three new hypoglycemic drugs in the treatment of ventricular remodeling, this study has important clinical implications for the treatment of T2DM combined with CVD, and can effectively guide the clinical use of drugs. However, the results of this meta-analysis should be interpreted with due considerations to the limitations. First, we included patients with T2DM, CVD, and the first two comorbidities. This may result in inter-study heterogeneity, with some effect on the overall results, in consideration of which, corresponding subgroup analyses were performed. Second, the ventricular structure changes estimated by echocardiography are variable, which may exaggerate or ignore the therapeutic effect. Third, the number of articles and participants in the analysis of e' and 6MWT indicators was relatively small, there was a lack of controlled clinical trials with a large sample size to conduct a more powerful demonstration of our outcome.

## Conclusion

The results of the network meta-analysis suggested that SGLT-2i may be more effective in cardiac remodeling compared with GLP-1RA and DPP-4i. In contrast, GLP-1RA and DPP-4i may have a tendency to improve cardiac systolic and diastolic function, respectively. This analysis provides valuable reference for the treatment of T2DM and/or CVD. However, the best treatment should be decided based on the individual patient, safety outcomes, and patient, caregiver, and clinician preferences. And more high-quality, large-sample, multicenter, randomized, double-blind trials are needed to confirm the reliability of the findings.

## Supplementary Information

**Additional file 1.**

**Additional file 2:** **Table S1.** Characteristics of included studies.

**Additional file 3:** **Figure S1.** Risk of bias graph. **Figure S2.** Risk of bias summary. **Table S2.** The Newcastle-Ottawa Quality Assessment Scale for included controlled studies.

**Additional file 4:** **Figure S3.**Network plot for overall population.Network plot of LVEF.Network plot of LVEDD.Network plot of LVEDV.Network plot of LVESD.Network plot of LVESV.Network plot of LVMI.Network plot of e’.Network plot of E/e’.Network plot of E/A.Network plot of SBP.Network plot of NT-pro BNP.Network plot of 6MWT.Network plot for subgroup of patients with T2DM and CVD.Network plot of LVEF.Network plot of LVEDV.Network plot of LVESV.Network plot of E/e’.Network plot of SBP.Network plot for subgroup of patients with CVD alone.Network plot of LVEF.Network plot of NT-pro BNP.Network plot of 6MWT. Note: e’: early diastolic velocity; E/e’: mitral inflow E velocity to tissue doppler e’ ratio; E/A: early diastolic to late diastolic velocities ratio; CVD: cardiovascular disease; DPP-4i: dipeptidyl peptidase-4 inhibitor; GLP-1RA: glucagon-like peptide-1 receptor agonist; LVEDD: left ventricular end-diastolic diameter; LVEDV: LV end-diastolic volume; LVEF: LV ejection fraction; LVESD: LV end-systolic diameter; LVESV: LV end-systolic volume; LVMI: LV mass index; NT-pro BNP: immunoreactive amino-terminal pro-brain natriuretic peptide; SBP: systolic blood pressure; SGLT-2i: sodium glucose cotransporter type 2 inhibitor; T2DM: type 2 diabetes; 6MWT: 6-min walk test.

**Additional file 5:** **Figure S4.** Funnel plot of mean difference.Funnel plot of mean difference of LVEF%. Note: A=DDP-4i, B=GLP-1RA, C=Placebo, D=SGLT-2i.Funnel plot of mean difference of LVEDD. Note: A=DDP-4i, B=GLP-1RA, C=Placebo, D=SGLT-2i.Funnel plot of mean difference of LVEDV.Funnel plot of mean difference of LVEDV.Funnel plot of mean difference of LVESD. Note: A=GLP-1RA, B=Placebo, C=SGLT-2i.Funnel plot of mean difference of LVESV. Note: A=GLP-1RA, B=Placebo, C=SGLT-2i.Funnel plot of mean difference of LVMI. Note: A=DDP-4i, B=GLP-1RA, C=Placebo, D=SGLT-2i.Funnel plot of mean difference of e’. Note: A=DDP-4i, B=GLP-1RA, C=Placebo, D=SGLT-2i.Funnel plot of mean difference of E/e’. Note: A=DDP-4i, B=GLP-1RA, C=Placebo, D=SGLT-2i.Funnel plot of mean difference of E/A. Note: A=DDP-4i, B=GLP-1RA, C=Placebo, D=SGLT-2i.Funnel plot of mean difference of SBP Note: A=DDP-4i, B=GLP-1RA, C=Placebo, D=SGLT-2i.Funnel plot of mean difference of NT-pro BNP Note: A=DDP-4i, B=GLP-1RA, C=Placebo, D=SGLT-2i.Funnel plot of mean difference of 6MWT Note: A= GLP-1RA, B=Placebo, C=SGLT-2i.Funnel plot of mean difference of LVEF% Note: A=DDP-4i, B=GLP-1RA, C=Placebo, D=SGLT-2i.Funnel plot of mean difference of LVEDV Note: A=DDP-4i, B=GLP-1RA, C=Placebo, D=SGLT-2i.Funnel plot of mean difference of LVESV Note: A=DDP-4i, B=GLP-1RA, C=Placebo, D=SGLT-2i.Funnel plot of mean difference of E/e’ Note: A=DDP-4i, B=GLP-1RA, C=Placebo, D=SGLT-2i.Funnel plot of mean difference of SBP Note: A=DDP-4i, B=GLP-1RA, C=Placebo, D=SGLT-2i.Funnel plot of mean difference of LVEF% Note: A=GLP-1RA, B=Placebo, C=SGLT-2i.Funnel plot of mean difference of NT-pro BNP Note: A = GLP-1RA, B = Placebo, C = SGLT-2i.Funnel plot of mean difference of 6MWT Note: A = GLP-1RA, B = Placebo, C = SGLT-2i Note: e’: early diastolic velocity; E/e’: mitral inflow E velocity to tissue doppler e’ ratio; E/A: early diastolic to late diastolic velocities ratio; CVD: cardiovascular disease; DPP-4i: dipeptidyl peptidase-4 inhibitor; GLP-1RA: glucagon-like peptide-1 receptor agonist; LVEDD: left ventricular end-diastolic diameter; LVEDV: LV end-diastolic volume; LVEF: LV ejection fraction; LVESD: LV end-systolic diameter; LVESV: LV end-systolic volume; LVMI: LV mass index; NT-pro BNP: immunoreactive amino-terminal pro-brain natriuretic peptide; SBP: systolic blood pressure; SGLT-2i: sodium glucose cotransporter type 2 inhibitor; T2DM: type 2 diabetes; 6MWT: 6-min walk test.

**Additional file 6:** **Figure S5.** Forest plot of mean difference.Forest plot of mean difference of LVEF%.Forest plot of mean difference of LVEDD.Forest plot of mean difference of LVEDV.Forest plot of mean difference of LVESD.Forest plot of mean difference of LVESV.Forest plot of mean difference of LVM.Forest plot of mean difference of e’.Forest plot of mean difference of E/e’.Forest plot of mean difference of E/A.Forest plot of mean difference of SBP.Forest plot of mean difference of NT-pro BNP.Forest plot of mean difference of 6MWT.Forest plot of mean difference of LVEF%.Forest plot of mean difference of LVEDV.Forest plot of mean difference of LVESV.Forest plot of mean difference of E/e’.Forest plot of mean difference of SBP.Forest plot of mean difference of LVEF%.Forest plot of mean difference of NT-pro BNP.Forest plot of mean difference of 6MWT. Note: e’: early diastolic velocity; E/e’: mitral inflow E velocity to tissue doppler e’ ratio; E/A: early diastolic to late diastolic velocities ratio; CVD: cardiovascular disease; DPP-4i: dipeptidyl peptidase-4 inhibitor; GLP-1RA: glucagon-like peptide-1 receptor agonist; LVEDD: left ventricular end-diastolic diameter; LVEDV: LV end-diastolic volume; LVEF: LV ejection fraction; LVESD: LV end-systolic diameter; LVESV: LV end-systolic volume; LVMI: LV mass index; NT-pro BNP: immunoreactive amino-terminal pro-brain natriuretic peptide; SBP: systolic blood pressure; SGLT-2i: sodium glucose cotransporter type 2 inhibitor; T2DM: type 2 diabetes; 6MWT: 6-min walk test.

**Additional file 7:** **Table S3.** Treatment Rankings. Note: e’: early diastolic velocity; E/e’: mitral inflow E velocity to tissue doppler e’ ratio; E/A: early diastolic to late diastolic velocities ratio; CVD: cardiovascular disease; DPP-4i: dipeptidyl peptidase-4 inhibitor; GLP-1RA: glucagon-like peptide-1 receptor agonist; LVEDD: left ventricular end-diastolic diameter; LVEDV: LV end-diastolic volume; LVEF: LV ejection fraction; LVESD: LV end-systolic diameter; LVESV: LV end-systolic volume; LVMI: LV mass index; NT-pro BNP: immunoreactive amino-terminal pro-brain natriuretic peptide; SBP: systolic blood pressure; SGLT-2i: sodium glucose cotransporter type 2 inhibitor; T2DM: type 2 diabetes; 6MWT: 6-min walk test.

**Additional file 8:** **Figure S6.** Curve diagram of SUCRA of outcome indicators.Curve diagram of SUCRA of LVEF% Note: 1 DDP-4i, 2 GLP-1RA, 3 Placebo, 4 SGLT-2i.Curve diagram of SUCRA of LVEDD Note: 1 DDP-4i, 2 GLP-1RA, 3 Placebo, 4 SGLT-2i.Curve diagram of SUCRA of LVEDV Note: 1 DDP-4i, 2 GLP-1RA, 3 Placebo, 4 SGLT-2i.Curve diagram of SUCRA of LVESD Note: 1 GLP-1RA, 2 Placebo, 3 SGLT-2i.Curve diagram of SUCRA of LVESV Note: 1 GLP-1RA, 2 Placebo, 3 SGLT-2i.Curve diagram of SUCRA of LVMI Note: 1 DDP-4i, 2 GLP-1RA, 3 Placebo, 4 SGLT-2i.Curve diagram of SUCRA of e’ Note: 1 DDP-4i, 2 GLP-1RA, 3 Placebo, 4 SGLT-2i.Curve diagram of SUCRA of E/e’ Note: 1 DDP-4i, 2 GLP-1RA, 3 Placebo, 4 SGLT-2i.Curve diagram of SUCRA of E/A Note: 1 DDP-4i, 2 GLP-1RA, 3 Placebo, 4 SGLT-2i.Curve diagram of SUCRA of SBP Note: 1 DDP-4i, 2 GLP-1RA, 3 Placebo, 4 SGLT-2i.Curve diagram of SUCRA of NT-pro BNP Note: 1 DDP-4i, 2 GLP-1RA, 3 Placebo, 4 SGLT-2i.Curve diagram of SUCRA of 6MWT Note: 1 GLP-1RA, 2 Placebo, 3 SGLT-2i.Curve diagram of SUCRA of LVEF%. Note: 1 DDP-4i, 2 GLP-1RA, 3 Placebo, 4 SGLT-2i.Curve diagram of SUCRA of LVEDV Note: 1 DDP-4i, 2 GLP-1RA, 3 Placebo, 4 SGLT-2i.Curve diagram of SUCRA of LVESV Note: 1 DDP-4i, 2 GLP-1RA, 3 Placebo, 4 SGLT-2i.Curve diagram of SUCRA of E/e’ Note: 1 DDP-4i, 2 GLP-1RA, 3 Placebo, 4 SGLT-2i.Curve diagram of SUCRA of SBP Note: 1 DDP-4i, 2 GLP-1RA, 3 Placebo, 4 SGLT-2i.Curve diagram of SUCRA of LVEF%. Note: 1 GLP-1RA, 2 Placebo, 3 SGLT-2i.Curve diagram of SUCRA of NT-pro BNP. Note: 1 GLP-1RA, 2 Placebo, 3 SGLT-2i.Curve diagram of SUCRA of 6MWT. Note: 1 GLP-1RA, 2 Placebo, 3 SGLT-2i Note: e’: early diastolic velocity; E/e’: mitral inflow E velocity to tissue doppler e’ ratio; E/A: early diastolic to late diastolic velocities ratio; CVD: cardiovascular disease; DPP-4i: dipeptidyl peptidase-4 inhibitor; GLP-1RA: glucagon-like peptide-1 receptor agonist; LVEDD: left ventricular end-diastolic diameter; LVEDV: LV end-diastolic volume; LVEF: LV ejection fraction; LVESD: LV end-systolic diameter; LVESV: LV end-systolic volume; LVMI: LV mass index; NT-pro BNP: immunoreactive amino-terminal pro-brain natriuretic peptide; SBP: systolic blood pressure; SGLT-2i: sodium glucose cotransporter type 2 inhibitor; T2DM: type 2 diabetes; 6MWT: 6-min walk test.

**Additional file 9:** **Table S3.**Matrix of pairwise comparisons of regmins on change of LVEF% in subgroup of patients with CVD alone.Matrix of pairwise comparisons of regimens on change of LVEF% in subgroup of patients with CVD and T2DM.Matrix of pairwise comparisons of regimens on change of E/e’ in subgroup of patients with CVD and T2DM.Matrix of pairwise comparisons of regimens on change of LVESV in subgroup of patients with CVD and T2DM.Matrix of pairwise comparisons of regimens on change of LVEDV in subgroup of patients with CVD and T2DM.Matrix of pairwise comparisons of regimens on change of SBP in subgroup of patients with CVD and T2DM.Matrix of pairwise comparisons of regmins on change of NT-pro BNP in subgroup of patients with CVD alone.Matrix of pairwise comparisons of regmins on change of 6MWT in subgroup of patients with CVD alone.Matrix of pairwise comparisons of regimens on change of e' in overall population.Matrix of pairwise comparisons of regimens on change of E/e' in overall population.Matrix of pairwise comparisons of regimens on change of E/A in overall population.Matrix of pairwise comparisons of regimens on change of SBP in overall population.Matrix of pairwise comparisons of regimens on change of NT-pro BNP in overall population.Matrix of pairwise comparisons of regimens on change of 6MWT in overall population.

**Additional file 10.**

## Data Availability

All data relevant to the study are included in the article or uploaded as supplementary information.
