# Supplementary material for: Effects of new hypoglycemic drugs on cardiac remodeling: a systematic review and network meta-analysis
Source: BMC Cardiovasc Disord. 2023 Jun 9;23:293. doi: 10.1186/s12872-023-03324-6 (PMC10251583; doi:10.1186/s12872-023-03324-6)
Supplement: Supplementary file 1 — Additional file 1. [file 12872_2023_3324_MOESM1_ESM.pdf]

Search strategy:
#1 ("Remodeling, Ventricular" OR "Ventricle Remodeling" OR "Remodeling, Ventricle" OR "Cardiac Remodeling, Ventricular" OR "Remodeling, Ventricular Cardiac" OR "Ventricular Cardiac Remodeling" OR "Myocardial Remodeling, Ventricular" OR "Remodeling, Ventricular Myocardial" OR "Ventricular Myocardial Remodeling" OR "Left Ventricle Remodeling" OR "Remodeling, Left Ventricle" OR "Ventricle Remodeling, Left" OR "Left Ventricular Remodeling" OR "Remodeling, Left Ventricular" OR "Ventricular Remodeling, Left" OR "cardiac reverse remodeling" OR "left ventricular dysfunction" OR "LVD" OR "CRR" OR "ejection fraction" OR "EF" OR "left ventricular ejection fraction" OR "LVEF" OR "end diastolic volume" OR "EDV" OR "end diastolic dimension" OR "EDD" OR "end systolic volume" OR "ESV" OR "end systolic dimension" OR "ESD" OR "LVEDD" OR "left ventricular end diastolic dimension" OR "LVEDV" OR "left ventricular end diastolic volume" OR "LVESD" OR "left ventricular end systolic dimension" OR "LVESV" OR "left ventricular end systolic volume" OR "left ventricular diameter" OR "left ventricular volume" OR "left ventricular mass index" OR "LVMI" OR "left atrial volume" OR "LAV" OR "left atrial volume index" OR "LAVI")

#2 ("dipeptidyl peptidase iv inhibitors" OR "dpp 4 inhibitors" OR "sodium glucose transporter 2 inhibitors" OR "sglt 2 inhibitors" OR "glucagon like peptide 1 agonists" OR "glp 1 agonists" OR "exenatide" OR "lyxumia" OR "liraglutide" OR "saxenda" OR "tanzeum" OR "albiglutide" OR "trulicity" OR "dulaglutide" OR "canagliflozin" OR "dapagliflozin" OR "empagliflozin" OR "ertugliflozin" OR "ipragliflozin" OR "luseogliflozin" OR "tofogliflozin" OR "sitagliptin" OR "vildagliptin" OR "saxagliptin" OR "alogliptin" OR "linagliptin" OR "gemigliptin" OR "teneligliptin")

#3 ("randomized controlled trial" OR "controlled clinical trial" OR “randomized" OR "randomly" OR "placebo")

#4: #1 and #2 and #3 ("Remodeling, Ventricular" OR "Ventricle Remodeling" OR "Remodeling, Ventricle" OR "Cardiac Remodeling, Ventricular" OR "Remodeling, Ventricular Cardiac" OR "Ventricular Cardiac Remodeling" OR "Myocardial Remodeling, Ventricular" OR "Remodeling, Ventricular Myocardial" OR "Ventricular Myocardial Remodeling" OR "Left Ventricle Remodeling" OR "Remodeling, Left Ventricle" OR "Ventricle Remodeling, Left" OR "Left Ventricular Remodeling" OR "Remodeling, Left Ventricular" OR "Ventricular Remodeling, Left" OR "cardiac reverse remodeling" OR "left ventricular dysfunction" OR "LVD" OR "CRR" OR "ejection fraction" OR "EF" OR "left ventricular ejection fraction" OR "LVEF" OR "end diastolic volume" OR "EDV" OR "end diastolic dimension" OR "EDD" OR "end systolic volume" OR "ESV" OR "end systolic dimension" OR "ESD" OR "LVEDD" OR "left ventricular end diastolic dimension" OR "LVEDV" OR "left ventricular end diastolic volume" OR "LVESD" OR "left ventricular end systolic dimension" OR "LVESV" OR "left ventricular end systolic volume" OR "left ventricular diameter" OR "left ventricular volume" OR "left ventricular mass index"

OR "LVMI" OR "left atrial volume" OR "LAV" OR "left atrial volume index" OR "LAVI") AND ("dipeptidyl peptidase iv inhibitors" OR "dpp 4 inhibitors" OR "sodium glucose transporter 2 inhibitors" OR "sglt 2 inhibitors" OR "glucagon like peptide 1 agonists" OR "glp 1 agonists" OR "exenatide" OR "lyxumia" OR "liraglutide" OR "saxenda" OR "tanzeum" OR "albiglutide" OR "trulicity" OR "dulaglutide" OR "canagliflozin" OR "dapagliflozin" OR "empagliflozin" OR "ertugliflozin" OR "ipragliflozin" OR "luseogliflozin" OR "tofogliflozin" OR "sitagliptin" OR "vildagliptin" OR "saxagliptin" OR "alogliptin" OR "linagliptin" OR "gemigliptin" OR "teneligliptin") AND ("randomized controlled trial" OR "controlled clinical trial" OR “randomized" OR "randomly" OR "placebo")
