# Supplementary material for: Effects of new hypoglycemic drugs on cardiac remodeling: a systematic review and network meta-analysis
Source: BMC Cardiovasc Disord. 2023 Jun 9;23:293. doi: 10.1186/s12872-023-03324-6 (PMC10251583; doi:10.1186/s12872-023-03324-6)
Supplement: Supplementary file 2 — Additional file 2: Table S1. Characteristics of included studies. [file 12872_2023_3324_MOESM2_ESM.pdf]

Table S1 Characteristics of included studies

Study	Year	Age	Male%	Patients	Sample size	Treatmet	Country
Ikonomidis et al.[1]	2020	58(10)	71.9	T2DM+CVD	80	GLP-1RA, SGLT-2i	Greece
Paiman et al.[2]	2019	55 (11)	40.0	T2DM	47	GLP-1RA	Netherlands
Santos-Gallego et al.[3]	2021	62(12.1)	64.0	CVD	80	SGLT-2i	USA
Akasaka et al.[4]	2022	71.9(8)	60.3	T2DM+CVD	68	SGLT-2i	Japan
Rau et al.[5]	2021	62(6.8)	77.2	T2DM	42	SGLT-2i	Germany
Verma et al.[6]	2019	63.3(9)	46.4	T2DM+CVD	90	SGLT-2i	Canada
Hiruma et al.[7]	2021	52.8(9.7)	72.0	T2DM	42	SGLT-2i	Japan
Brown et al.[8]	2020	65.53(6.87)	57.6	T2DM+CVD	66	SGLT-2i	UK
Bojer et al.[9]	2021	62.2 (10)	80.0	T2DM+CVD	40	GLP-1RA	Denmark
Cioffi et al.[10]	2021	69(9)	55.8	T2DM	187	DPP-4i	Italy

Singh et al.[11]	2020	67.1(6.9)	66.1	T2DM+CVD	56	SGLT-2i	UK
Bami et al.[12]	2020	63.3 (9)	92.7	T2DM+CVD	97	SGLT-2i	Canada
Webb et al.[13]	2020	43.4(7)	46.0	T2DM	61	DPP-4i, GLP-1RA	UK
Omar et al.[14]	2021	64(11)	85.3	T2DM+CVD	179	SGLT-2i	Denmark
Oldgren et al.[15]	2021	64.4(7.2)	53.0	T2DM	49	SGLT-2i	Sweden
Ersbøll et al.[16]	2022	66.2 (9.2)	80.5	T2DM+CVD	87	SGLT-2i	Denmark
Lee et al.[17]	2020	68.7(11.1)	73.3	T2DM+CVD	92	SGLT-2i	UK
Bizino et al.[18]	2019	60(6)	59.2	T2DM	49	GLP-1RA	Netherlands
Otagaki et al.[19]	2019	65(13.8)	69.1	T2DM	42	SGLT-2i	Japan
Chen et al.[20]	2015	57.7(11.3)	73.3	CVD	83	GLP-1RA	China
Jorsal et al.[21]	2017	65(9.2)	89.2	T2DM+CVD	241	GLP-1RA	Denmark
Lepore et al.[22]	2016	56(10)	74.0	CVD	56	GLP-1RA	USA

Sardu et al.[23]	2018	72(6)	72.1	T2DM+CVD	559	GLP-1RA	Italy
Yamada et al.[24]	2017	69(8)	67.0	T2DM	115	DPP-4i	Japan
Yamamoto et al.[25]	2017	71(10)	63.8	CVD+T2DM	137	DPP-4i	Japan
Zhang et al.[26]	2017	59.1(11.8)	75.0	CVD	52	GLP-1RA	China
Zhang et al.[27]	2022	56.32(12.2)	83.3	CVD	56	SGLT-2i	China
E.Reis et al.[28]	2022	60.9(13)	82.5	CVD	40	SGLT-2i	Portugal
E.Nassif et al.[29]	2021	70.1(9.68)	49.4	CVD	324	SGLT-2i	USA
Mason et al.[30]	2021	62(8)	91.9	T2DM+CVD	74	SGLT-2i	Canada
Kumarathurai et al.[31]	2021	63.1(6.6)	80.0	T2DM+CVD	58	GLP-1RA	Denmark
Abraham et al.[32]	2021	69.7(9.7)	65.0	CVD	627	SGLT-2i	USA
Gamaza-Chulián et al.[33]	2021	66.8(8.6)	55.8	T2DM	52	SGLT-2i	Spain
Jensen et al.[34]	2020	64.7(12)	85.3	CVD	186	SGLT-2i	Denmark
Nielsen et al.[35]	2020	65.2(9.4)	90.1	T2DM+CVD	208	GLP-1RA	Denmark

Note: CVD cardiovascular disease, DPP-4i dipeptidyl peptidase-4 inhibitor; GLP-1RA glucagon-like peptide-1 receptor agonist, SGLT-2i sodium glucose cotransporter type 2 inhibitor, T2DM type 2 diabetes mellitus.

List of included literature:

1. Ikonomidis I, Pavlidis G, Thymis J, et al. Effects of Glucagon-Like Peptide-1 Receptor Agonists, Sodium-Glucose Cotransporter-2 Inhibitors, and Their Combination on Endothelial Glycocalyx, Arterial Function, and Myocardial Work Index in Patients With Type 2 Diabetes Mellitus After 12-Month Treatment. J Am Heart Assoc. 2020;9(9):e015716. https://doi.org/10.1161/JAHA.119.015716
2. Paiman E, van Eyk HJ, van Aalst M, et al. Effect of Liraglutide on Cardiovascular Function and Myocardial Tissue Characteristics in Type 2 Diabetes Patients of South Asian Descent Living in the Netherlands: A Double-Blind, Randomized, Placebo-Controlled Trial. J Magn Reson Imaging. 2020;51(6):1679-88. https://doi.org/10.1002/jmri.27009
3. Santos-Gallego CG, Vargas-Delgado AP, Requena-Ibanez JA, et al. Randomized Trial of Empagliflozin in Nondiabetic Patients With Heart Failure and Reduced Ejection Fraction. J Am Coll Cardiol. 2021;77(3):243-55. https://doi.org/10.1016/j.jacc.2020.11.008
4. Akasaka H, Sugimoto K, Shintani A, et al. Effects of ipragliflozin on left ventricular diastolic function in patients with type 2 diabetes and heart failure with preserved ejection fraction: The EXCEED randomized controlled multicenter study. Geriatr Gerontol Int. 2022;22(4):298-304. https://doi.org/ 10.1186/s12933-020-01175-5
5. Rau M, Thiele K, Hartmann NK, et al. Empagliflozin does not change cardiac index nor systemic vascular resistance but rapidly improves left ventricular filling pressure in patients with type 2 diabetes: a randomized controlled study. Cardiovasc Diabetol. 2021;20(1):6. https://doi.org/ 10.1111/ggi.14363
6. Verma A, Meris A, Skali H, et al. Prognostic implications of left ventricular mass and geometry following myocardial infarction: the VALIANT (VALsartan In Acute myocardial iNfarcTion) Echocardiographic Study. JACC Cardiovasc Imaging. 2008;1(5):582-91. https://doi.org/10.1016/j.jcmg.2010.10.008
7. Hiruma S, Shigiyama F, Hisatake S, et al. A prospective randomized study comparing effects of empagliflozin to sitagliptin on cardiac fat

accumulation, cardiac function, and cardiac metabolism in patients with early-stage type 2 diabetes: the ASSET study. Cardiovasc Diabetol. 2021;20(1):32. https://doi.org/10.1186/s12933-021-01228-3
8. Brown A, Gandy S, McCrimmon R, et al. A randomized controlled trial of dapagliflozin on left ventricular hypertrophy in people with type two diabetes: the DAPA-LVH trial. Eur Heart J. 2020;41(36):3421-32. https://doi.org/10.1093/eurheartj/ehaa419
9. Bojer AS, Sørensen MH, Bjerre J, et al. Metabolic improvement with short-term, glucagon-like peptide-1 receptor agonist treatment does not improve cardiac diastolic dysfunction in patients with type 2 diabetes: A randomized, double-blind, placebo-controlled trial. Diabetes Obes Metab. 2021;23(10):2374-84. https://doi.org/10.1177/2047487320939217
10. Cioffi G, Giorda CB, Lucci D, et al. Effects of linagliptin on left ventricular DYsfunction in patients with type 2 DiAbetes and concentric left ventricular geometry: results of the DYDA 2 trial. Eur J Prev Cardiol. 2021;28(1):8-17. https://doi.org/10.1016/j.echo.2020.02.005
11. Singh J, Mordi IR, Vickneson K, et al. Dapagliflozin Versus Placebo on Left Ventricular Remodeling in Patients With Diabetes and Heart Failure: The REFORM Trial. Diabetes Care. 2020;43(6):1356-9. https://doi.org/10.2337/dc19-2187
12. Bami K, Gandhi S, Leong-Poi H, et al. Effects of Empagliflozin on Left Ventricular Remodeling in Patients with Type 2 Diabetes and Coronary Artery Disease: Echocardiographic Substudy of the EMPA-HEART CardioLink-6 Randomized Clinical Trial. J Am Soc Echocardiogr. 2020;33(5):644-6. https://doi.org/10.1111/dom.14480
13. Webb DR, Htike ZZ, Swarbrick DJ, et al. A randomized, open-label, active comparator trial assessing the effects of 26 weeks of liraglutide or sitagliptin on cardiovascular function in young obese adults with type 2 diabetes. Diabetes Obes Metab. 2020;22(7):1187-96. https://doi.org/10.1111/dom.14023
14. Omar M, Jensen J, Ali M, et al. Associations of Empagliflozin With Left Ventricular Volumes, Mass, and Function in Patients With Heart Failure and Reduced Ejection Fraction: A Substudy of the Empire HF Randomized Clinical Trial. JAMA Cardiol. 2021;6(7):836-40. https://doi.org/ 10.1002/ejhf.657
15. Oldgren J, Laurila S, Åkerblom A, et al. Effects of 6 weeks of treatment with dapagliflozin, a sodium-glucose co-transporter-2 inhibitor, on myocardial function and metabolism in patients with type 2 diabetes: A randomized, placebo-controlled, exploratory study. Diabetes Obes Metab. 2021;23(7):1505-17. https://doi.org/10.1007/s12020-015-0798-0
16. Ersbøll M, Jürgens M, Hasbak P, et al. Effect of empagliflozin on myocardial structure and function in patients with type 2 diabetes at high cardiovascular risk: the SIMPLE randomized clinical trial. Int J Cardiovasc Imaging. 2022;38(3):579-87.

https://doi.org/10.1016/j.jchf.2016.01.008
17. Lee M, Brooksbank K, Wetherall K, et al. Effect of Empagliflozin on Left Ventricular Volumes in Patients With Type 2 Diabetes, or Prediabetes, and Heart Failure With Reduced Ejection Fraction (SUGAR-DM-HF). Circulation. 2021;143(6):516-25. https://doi.org/10.1007/s10557-019-06892-y
18. Bizino MB, Jazet IM, Westenberg J, et al. Effect of liraglutide on cardiac function in patients with type 2 diabetes mellitus: randomized placebo-controlled trial. Cardiovasc Diabetol. 2019;18(1):55. https://doi.org/10.1161/CIRCULATIONAHA.120.052186
19. Otagaki M, Matsumura K, Kin H, et al. Effect of Tofogliflozin on Systolic and Diastolic Cardiac Function in Type 2 Diabetic Patients. Cardiovasc Drugs Ther. 2019;33(4):435-42. https://doi.org/10.1186/s12933-019-0857-6
20. Chen WR, Shen XQ, Zhang Y, et al. Effects of liraglutide on left ventricular function in patients with non-ST-segment elevation myocardial infarction. Endocrine. 2016;52(3):516-26. https://doi.org/10.1001/jamacardio.2020.6827
21. Jorsal A, Kistorp C, Holmager P, et al. Effect of liraglutide, a glucagon-like peptide-1 analogue, on left ventricular function in stable chronic heart failure patients with and without diabetes (LIVE)-a multicentre, double-blind, randomised, placebo-controlled trial. Eur J Heart Fail. 2017;19(1):69-77. https://doi.org/10.1007/s10554-021-02443-5
22. Lepore JJ, Olson E, Demopoulos L, et al. Effects of the Novel Long-Acting GLP-1 Agonist, Albiglutide, on Cardiac Function, Cardiac Metabolism, and Exercise Capacity in Patients With Chronic Heart Failure and Reduced Ejection Fraction. JACC Heart Fail. 2016;4(7):559-66. https://doi.org/10.1111/dom.14363
23. Sardu C, Paolisso P, Sacra C, et al. Cardiac resynchronization therapy with a defibrillator (CRTd) in failing heart patients with type 2 diabetes mellitus and treated by glucagon-like peptide 1 receptor agonists (GLP-1 RA) therapy vs. conventional hypoglycemic drugs: arrhythmic burden, hospitalizations for heart failure, and CRTd responders rate. Cardiovasc Diabetol. 2018;17(1):137.
24. Yamada H, Tanaka A, Kusunose K, et al. Effect of sitagliptin on the echocardiographic parameters of left ventricular diastolic function in patients with type 2 diabetes: a subgroup analysis of the PROLOGUE study. Cardiovasc Diabetol. 2017;16(1):63. https://doi.org/10.1186/s12933-017-0546-2
25. Yamamoto M, Seo Y, Ishizu T, et al. Effect of Dipeptidyl Peptidase-4 Inhibitors on Cardiovascular Outcome and Cardiac Function in Patients With Diabetes and Heart Failure - Insights From the Ibaraki Cardiac Assessment Study-Heart Failure (ICAS-HF) Registry. Circ J. 2017;81(11):1662-9. https://doi.org/ 10.1253/circj.CJ-17-0240
26. Zhang JY, Wang XY, Wang X. Effects of liraglutide on hemodynamic parameters in patients with heart failure. Oncotarget.

2017;8(37):62693-702. https://doi.org/10.18632/oncotarget.18570
27. Zhang H, Liu Z. Effects of Dapagliflozin in Combination with Metoprolol Sustained-Release Tablets on Prognosis and Cardiac Function in Patients with Acute Myocardial Infarction after PCI. Comput Math Methods Med. 2022;2022:5734876. https://doi.org/10.1155/2022/5734876
28. Reis J, Teixeira AR, Gonçalves AV, et al. Dapagliflozin Impact on the Exercise Capacity of Non-Diabetic Heart Failure with Reduced Ejection Fraction Patients. J Clin Med. 2022;11(10). https://doi.org/10.3390/jcm11102935
29. Nassif ME, Windsor SL, Borlaug BA, et al. The SGLT2 inhibitor dapagliflozin in heart failure with preserved ejection fraction: a multicenter randomized trial. Nat Med. 2021;27(11):1954-60. https://doi.org/ 10.1111/dom.14135
30. Mason T, Coelho-Filho OR, Verma S, et al. Empagliflozin Reduces Myocardial Extracellular Volume in Patients With Type 2 Diabetes and Coronary Artery Disease. JACC Cardiovasc Imaging. 2021;14(6):1164-73. https://doi.org/10.1016/j.ahj.2020.07.011
31. Kumarathurai P, Sajadieh A, Anholm C, et al. Effects of liraglutide on diastolic function parameters in patients with type 2 diabetes and coronary artery disease: a randomized crossover study. Cardiovasc Diabetol. 2021;20(1):12. https://doi.org/10.1186/s12933-020-01205-2
32. Abraham WT, Lindenfeld J, Ponikowski P, et al. Effect of empagliflozin on exercise ability and symptoms in heart failure patients with reduced and preserved ejection fraction, with and without type 2 diabetes. Eur Heart J. 2021;42(6):700-10. https://doi.org/10.1093/eurheartj/ehaa943
33. Gamaza-Chulián S, Díaz-Retamino E, González-Testón F, et al. Effect of sodium-glucose cotransporter 2 (SGLT2) inhibitors on left ventricular remodelling and longitudinal strain: a prospective observational study. BMC Cardiovasc Disord. 2021;21(1):456. https://doi.org/10.1186/s12872-021-02250-9
34. Jensen J, Omar M, Kistorp C, et al. Twelve weeks of treatment with empagliflozin in patients with heart failure and reduced ejection fraction: A double-blinded, randomized, and placebo-controlled trial. Am Heart J. 2020;228:47-56. https://doi.org/10.1038/s41591-021-01536-x
35. Nielsen R, Jorsal A, Tougaard RS, et al. The impact of the glucagon-like peptide-1 receptor agonist liraglutide on natriuretic peptides in heart failure patients with reduced ejection fraction with and without type 2 diabetes. Diabetes Obes Metab. 2020;22(11):2141-50. https://doi.org/ 10.1016/j.jcmg.2020.10.017
