# Supplementary material for: Effects of new hypoglycemic drugs on cardiac remodeling: a systematic review and network meta-analysis
Source: BMC Cardiovasc Disord. 2023 Jun 9;23:293. doi: 10.1186/s12872-023-03324-6 (PMC10251583; doi:10.1186/s12872-023-03324-6)
Supplement: Supplementary file 3 — Additional file 3: Figure S1. Risk of bias graph. Figure S2. Risk of bias summary. Table S2. The Newcastle-Ottawa Quality Assessment Scale for included controlled studies. [file 12872_2023_3324_MOESM3_ESM.pdf]

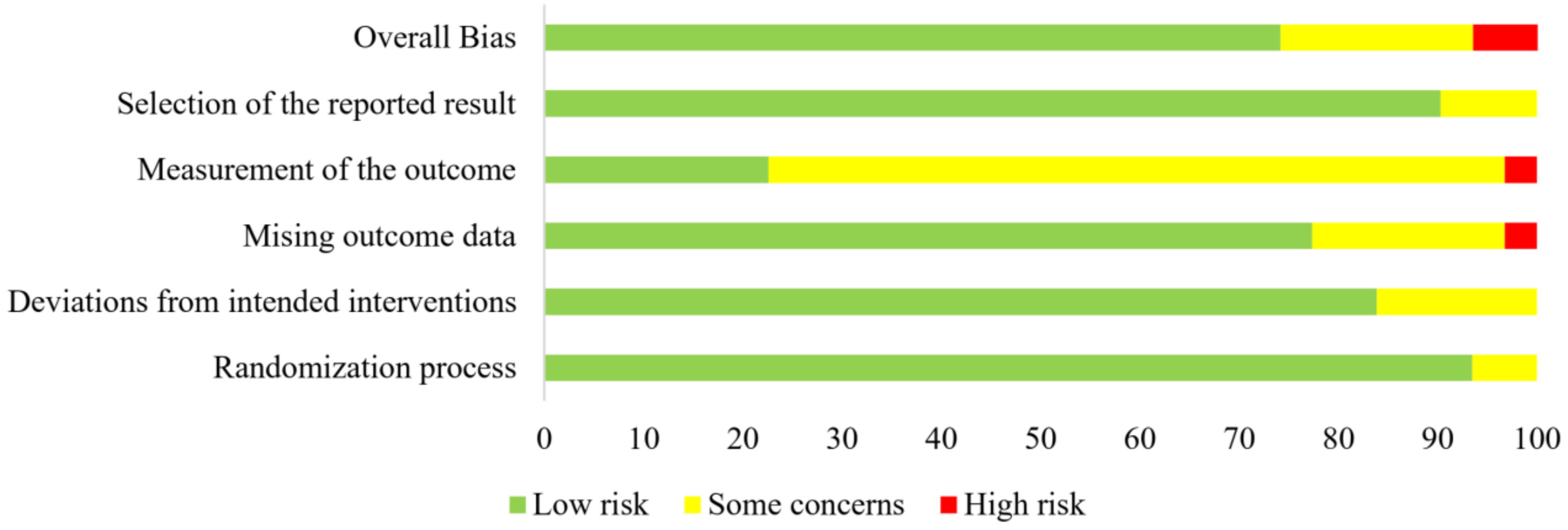

Figure S1 Risk of bias graph

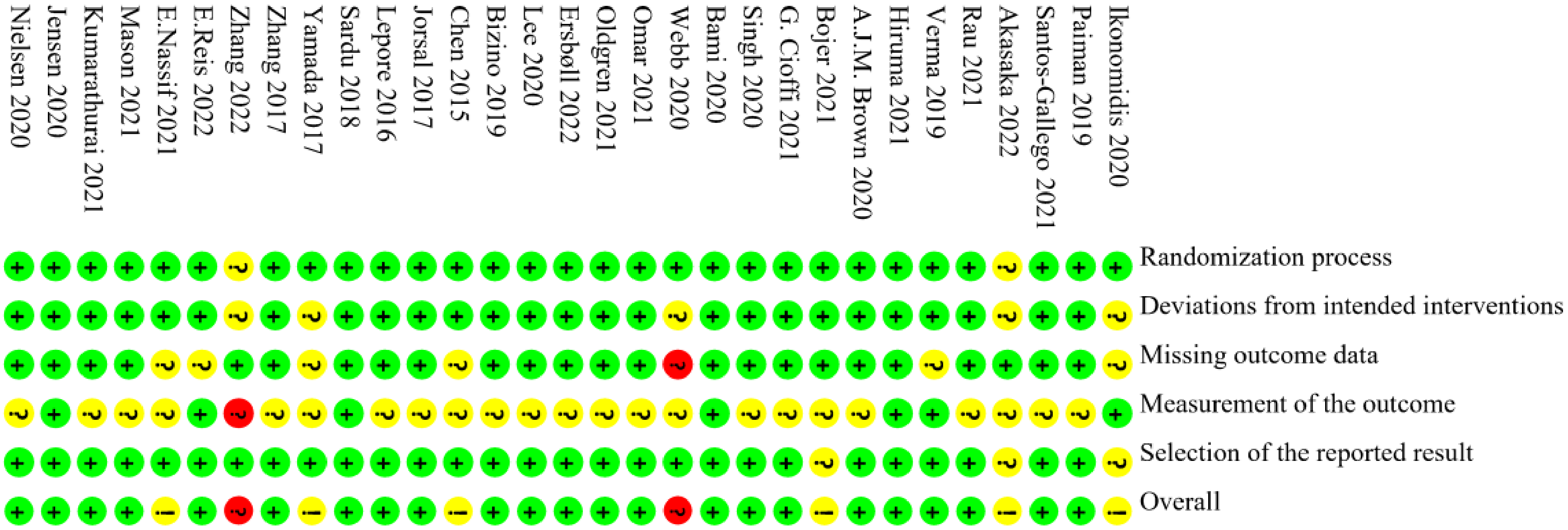

Figure S2 Risk of bias summary

Table S2 The Newcastle-Ottawa Quality Assessment Scale for included controlled studies

Study	Selection of the study groups	Comparability of the groups	Outcome	Total score
Otagaki et al.[19]	****	**	**	8
Sardu et al.[23]	****	**	***	9
Yamamoto et al.[25]	****	**	***	9
Gamaza-Chulián et al.[33]	****	*	***	8

Note: References in table S2 can be found in Additional file 2: characteristics of studies.
