# Supplementary material for: Effects of new hypoglycemic drugs on cardiac remodeling: a systematic review and network meta-analysis
Source: BMC Cardiovasc Disord. 2023 Jun 9;23:293. doi: 10.1186/s12872-023-03324-6 (PMC10251583; doi:10.1186/s12872-023-03324-6)
Supplement: Supplementary file 4 — Additional file 4: Figure S3.Network plot for overall population.Network plot of LVEF.Network plot of LVEDD.Network plot of LVEDV.Network plot of LVESD.Network plot of LVESV.Network plot of LVMI.Network plot of e’.Network plot of E/e’.Network plot of E/A.Network plot of SBP.Network plot of NT-pro BNP.Network plot of 6MWT.Network plot for subgroup of patients with T2DM and CVD.Network plot of LVEF.Network plot of LVEDV.Network plot of LVESV.Network plot of E/e’.Network plot of SBP.Network plot for subgroup of patients with CVD alone.Network plot of LVEF.Network plot of NT-pro BNP.Network plot of 6MWT. Note: e’: early diastolic velocity; E/e’: mitral inflow E velocity to tissue doppler e’ ratio; E/A: early diastolic to late diastolic velocities ratio; CVD: cardiovascular disease; DPP-4i: dipeptidyl peptidase-4 inhibitor; GLP-1RA: glucagon-like peptide-1 receptor agonist; LVEDD: left ventricular end-diastolic diameter; LVEDV: LV end-diastolic volume; LVEF: LV ejection fraction; LVESD: LV end-systolic diameter; LVESV: LV end-systolic volume; LVMI: LV mass index; NT-pro BNP: immunoreactive amino-terminal pro-brain natriuretic peptide; SBP: systolic blood pressure; SGLT-2i: sodium glucose cotransporter type 2 inhibitor; T2DM: type 2 diabetes; 6MWT: 6-min walk test. [file 12872_2023_3324_MOESM4_ESM.pdf]

Figure S3 (a-l) Network plot for overall population.

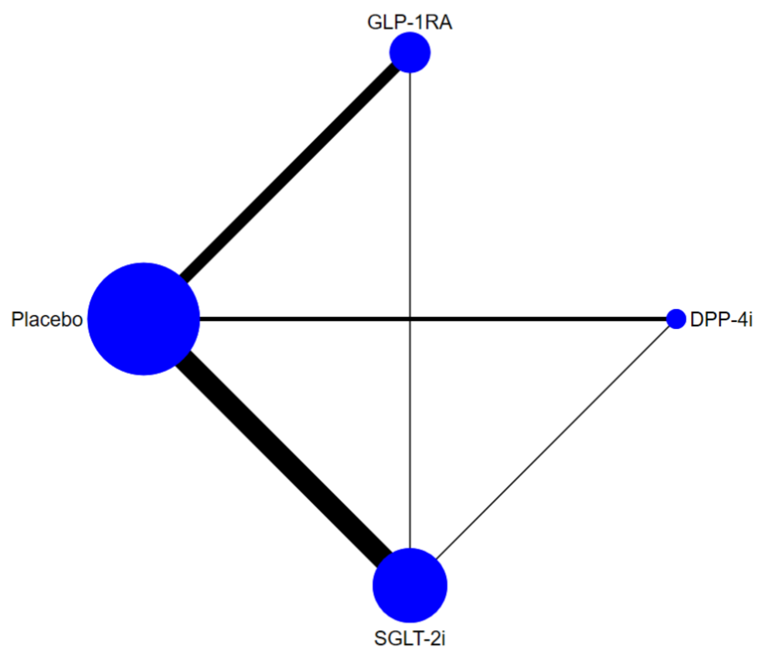

Figure S3 (a) Network plot of LVEF

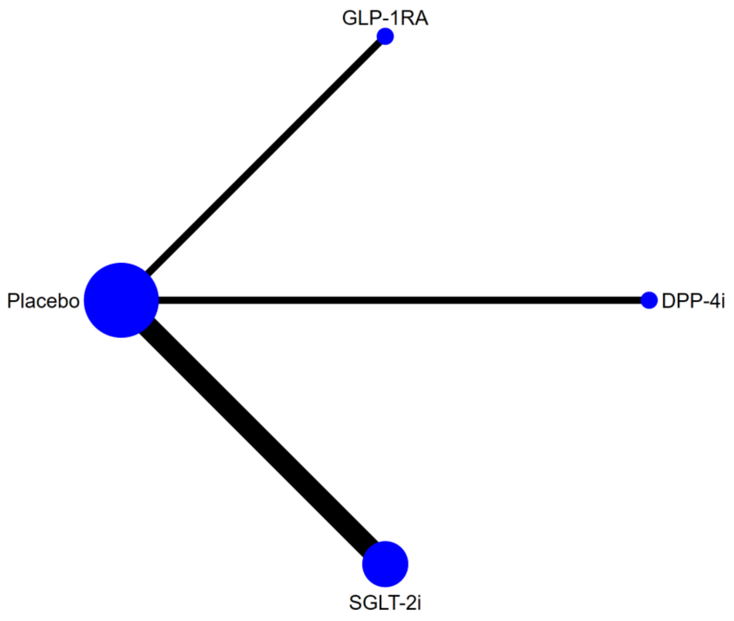

Figure S3 (b) Network plot of LVEDD

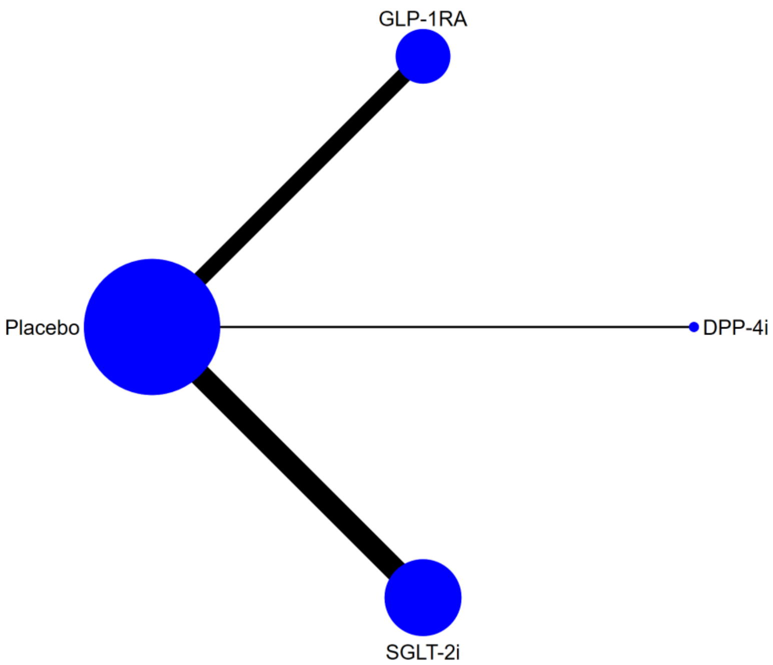

Figure S3 (c) Network plot of LVEDV

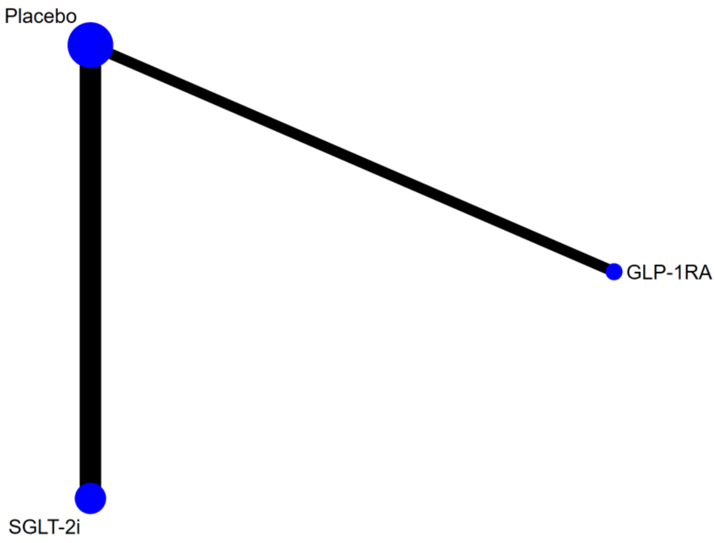

Figure S3 (d) Network plot of LVESD

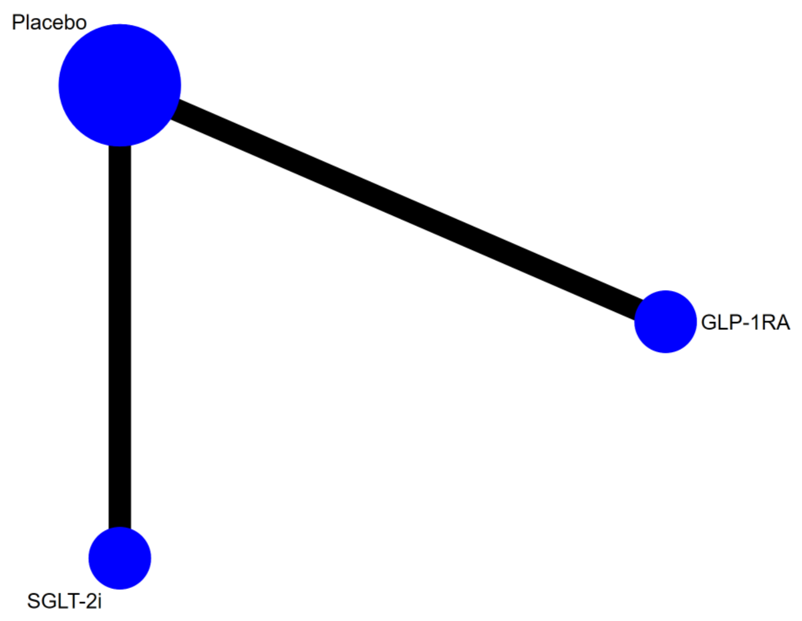

Figure S3 (e) Network plot of LVESV

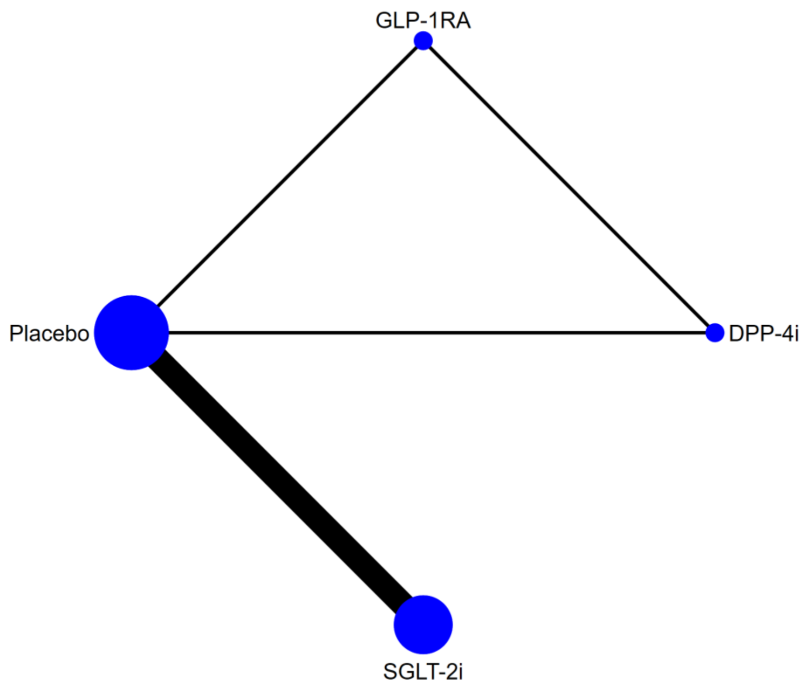

Figure S3 (f) Network plot of LVMI

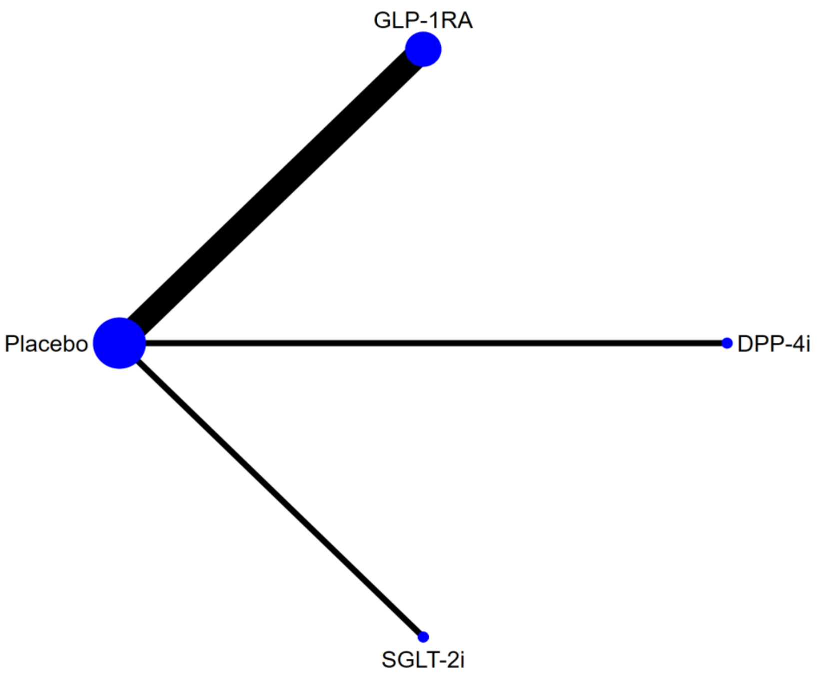

Figure S3 (g) Network plot of e’

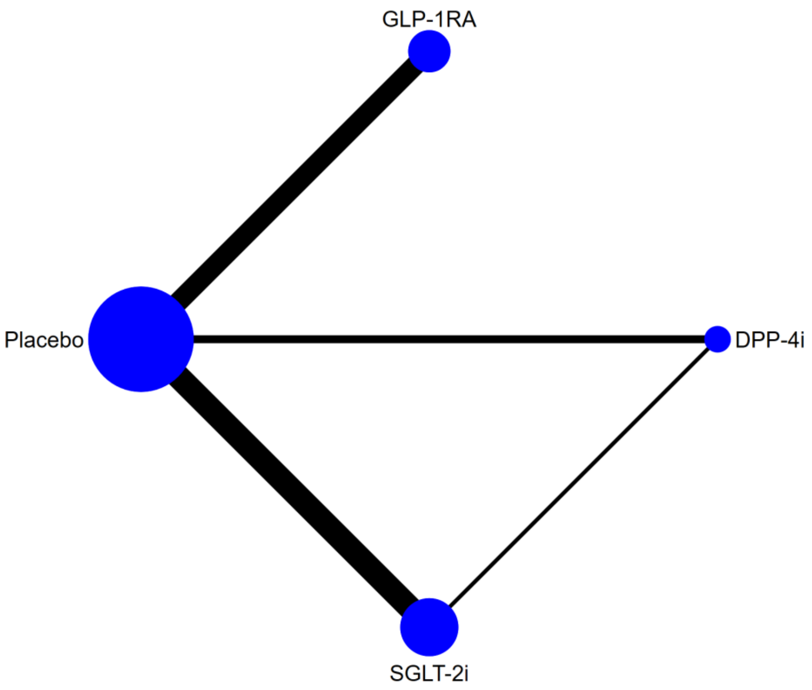

Figure S3 (h) Network plot of E/e’

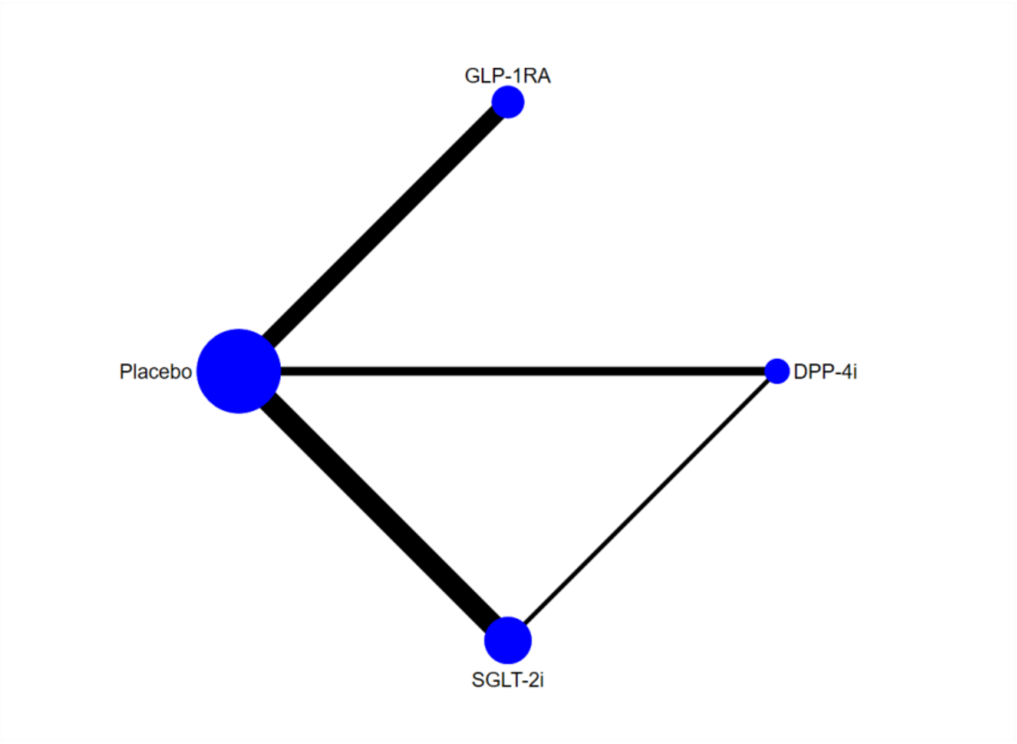

Figure S3 (i) Network plot of E/A

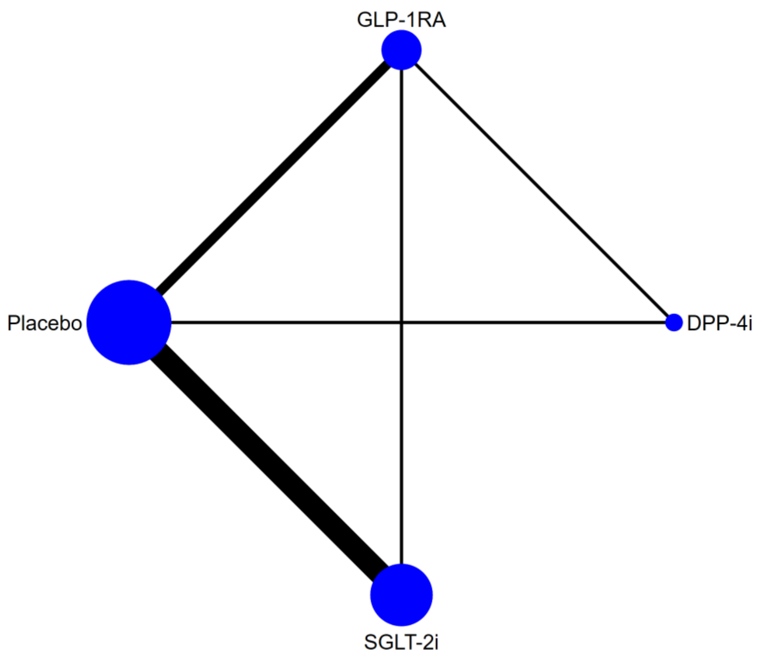

Figure S3 (j) Network plot of SBP

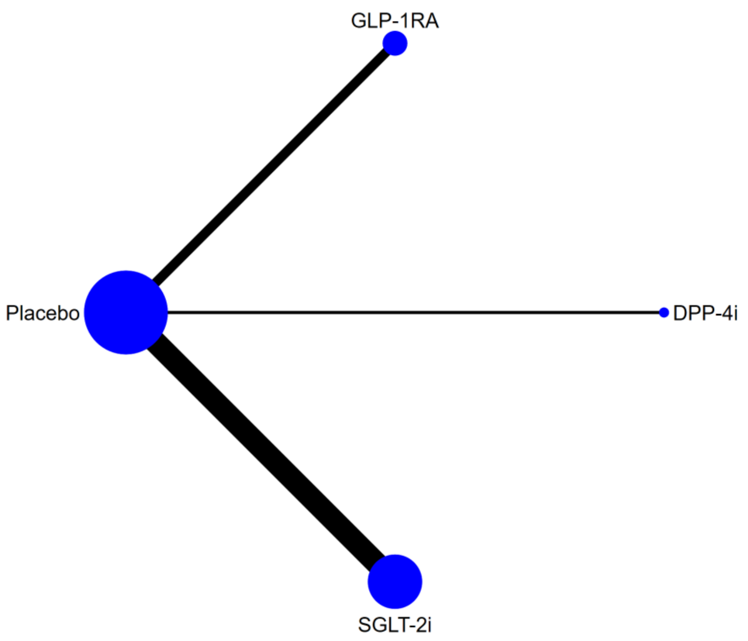

Figure S3 (k) Network plot of NT-pro BNP

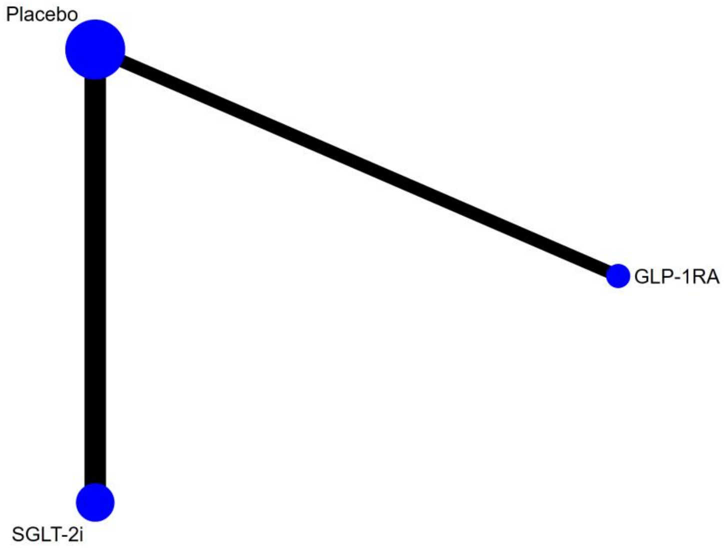

Figure S3 (l) Network plot of 6MWT

Figure S3 (m-q) Network plot for subgroup of patients with T2DM and CVD.

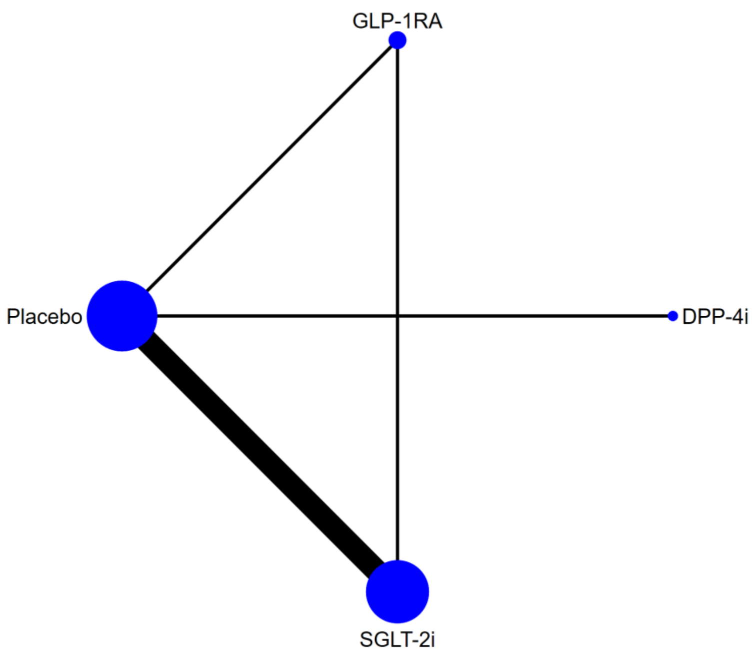

Figure S3 (m) Network plot of LVEF

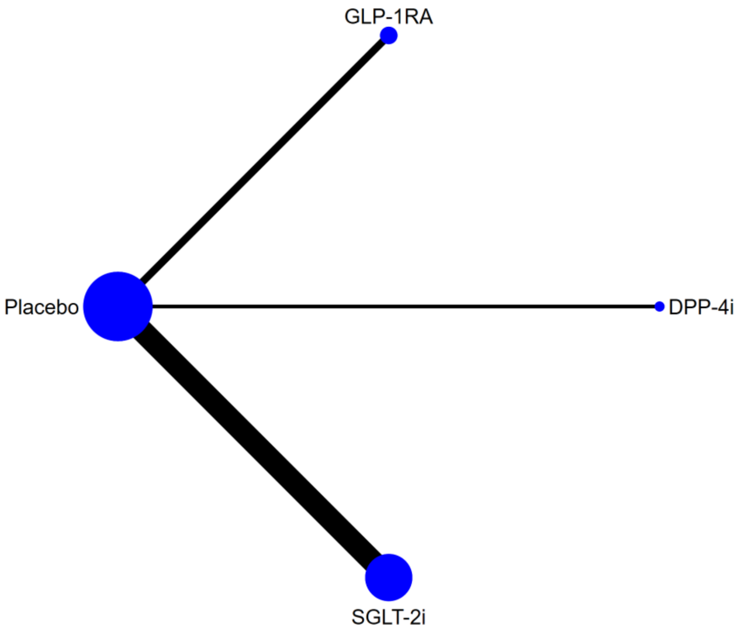

Figure S3 (n) Network plot of LVEDV

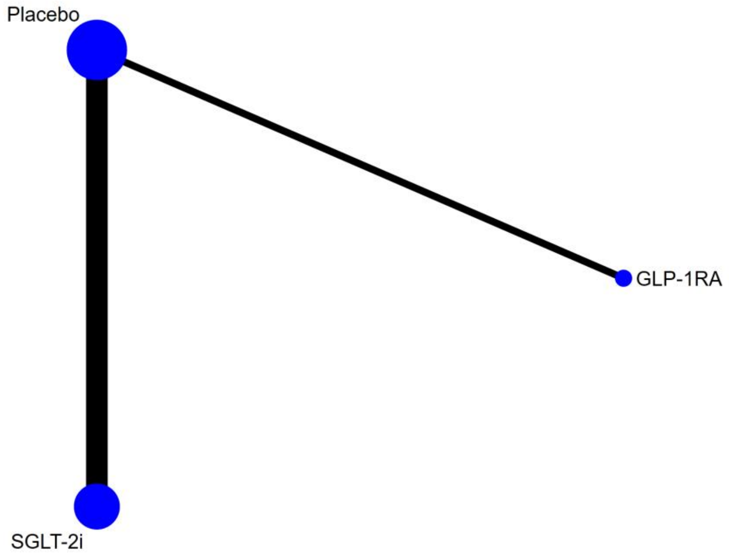

Figure S3 (o) Network plot of LVESV

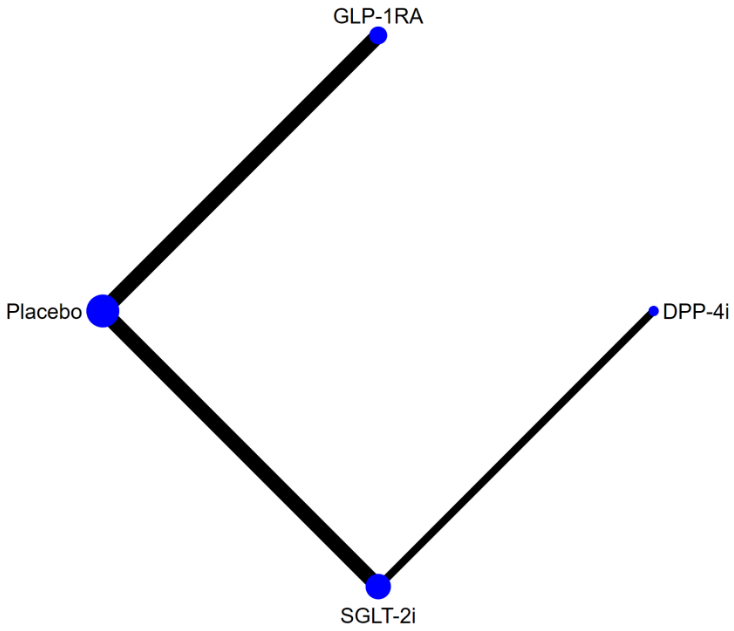

Figure S3 (p) Network plot of E/e’

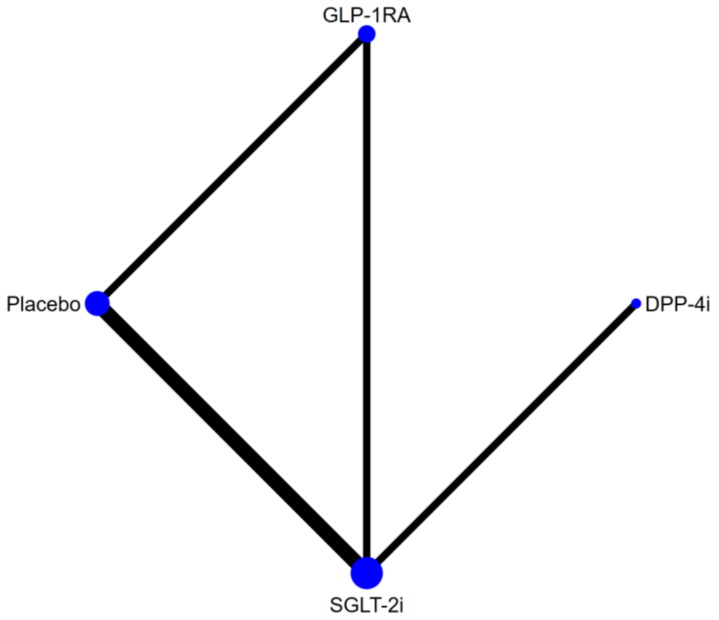

Figure S3 (q) Network plot of SBP

Figure S3 (r-t) Network plot for subgroup of patients with CVD alone.

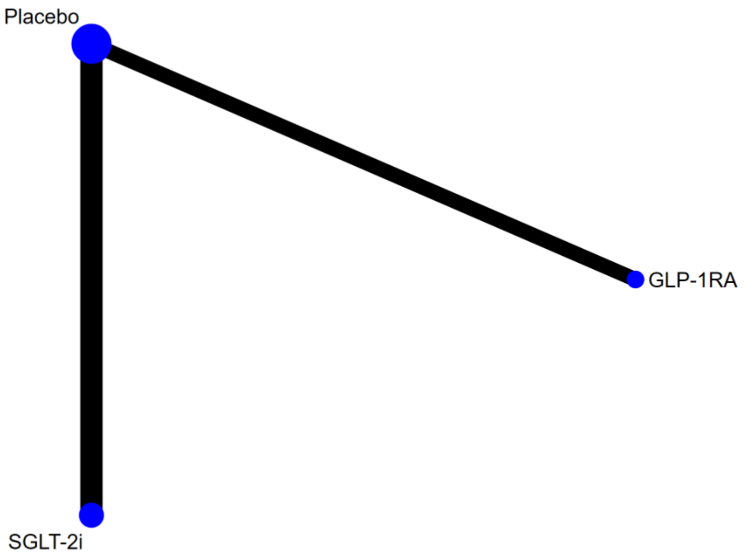

Figure S3 (r) Network plot of LVEF

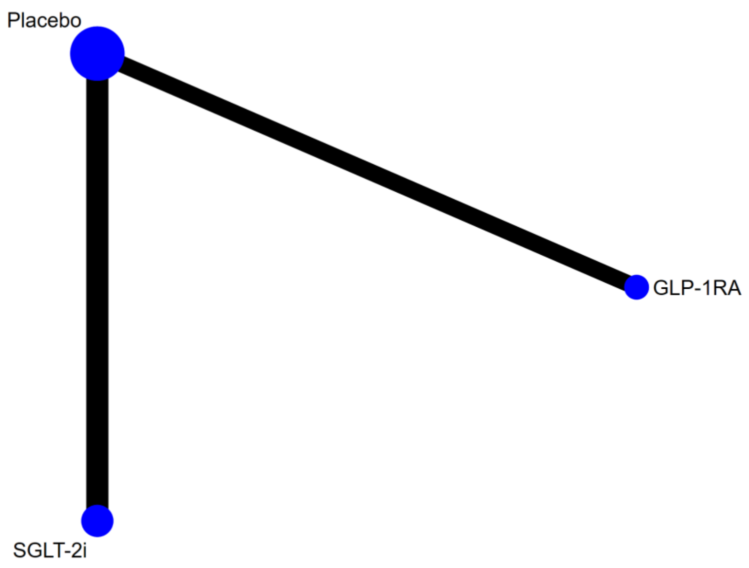

Figure S3 (s) Network plot of NT-pro BNP

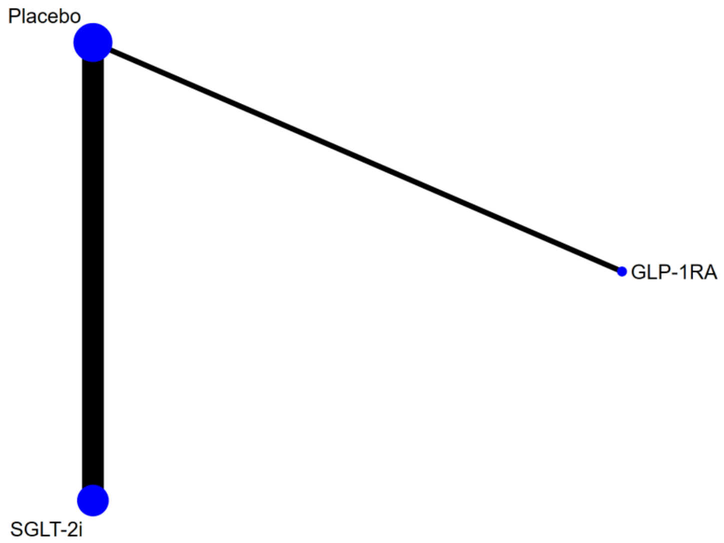

Figure S3 (t) Network plot of 6MWT

Note: e': early diastolic velocity; **E/e'**: mitral inflow E velocity to tissue doppler e' ratio; **E/A**: early diastolic to late diastolic velocities ratio**; CVD**: cardiovascular disease; **DPP-4i**: dipeptidyl peptidase-4 inhibitor; **GLP-1RA**: glucagon-like peptide-1 receptor agonist; **LVEDD**: left ventricular end-diastolic diameter; **LVEDV**: LV end-diastolic volume; **LVEF**: LV ejection fraction; **LVESD**: LV end-systolic diameter; **LVESV**: LV end-systolic volume;

LVMI: LV mass index; **NT-pro BNP**: immunoreactive amino-terminal pro-brain natriuretic peptide; **SBP**: systolic blood pressure; **SGLT-2i**: sodium glucose cotransporter type 2 inhibitor; **T2DM**: type 2 diabetes; **6MWT**: 6-min walk test.
