# Supplementary material for: Effects of new hypoglycemic drugs on cardiac remodeling: a systematic review and network meta-analysis
Source: BMC Cardiovasc Disord. 2023 Jun 9;23:293. doi: 10.1186/s12872-023-03324-6 (PMC10251583; doi:10.1186/s12872-023-03324-6)
Supplement: Supplementary file 5 — Additional file 5: Figure S4. Funnel plot of mean difference.Funnel plot of mean difference of LVEF%. Note: A=DDP-4i, B=GLP-1RA, C=Placebo, D=SGLT-2i.Funnel plot of mean difference of LVEDD. Note: A=DDP-4i, B=GLP-1RA, C=Placebo, D=SGLT-2i.Funnel plot of mean difference of LVEDV.Funnel plot of mean difference of LVEDV.Funnel plot of mean difference of LVESD. Note: A=GLP-1RA, B=Placebo, C=SGLT-2i.Funnel plot of mean difference of LVESV. Note: A=GLP-1RA, B=Placebo, C=SGLT-2i.Funnel plot of mean difference of LVMI. Note: A=DDP-4i, B=GLP-1RA, C=Placebo, D=SGLT-2i.Funnel plot of mean difference of e’. Note: A=DDP-4i, B=GLP-1RA, C=Placebo, D=SGLT-2i.Funnel plot of mean difference of E/e’. Note: A=DDP-4i, B=GLP-1RA, C=Placebo, D=SGLT-2i.Funnel plot of mean difference of E/A. Note: A=DDP-4i, B=GLP-1RA, C=Placebo, D=SGLT-2i.Funnel plot of mean difference of SBP Note: A=DDP-4i, B=GLP-1RA, C=Placebo, D=SGLT-2i.Funnel plot of mean difference of NT-pro BNP Note: A=DDP-4i, B=GLP-1RA, C=Placebo, D=SGLT-2i.Funnel plot of mean difference of 6MWT Note: A= GLP-1RA, B=Placebo, C=SGLT-2i.Funnel plot of mean difference of LVEF% Note: A=DDP-4i, B=GLP-1RA, C=Placebo, D=SGLT-2i.Funnel plot of mean difference of LVEDV Note: A=DDP-4i, B=GLP-1RA, C=Placebo, D=SGLT-2i.Funnel plot of mean difference of LVESV Note: A=DDP-4i, B=GLP-1RA, C=Placebo, D=SGLT-2i.Funnel plot of mean difference of E/e’ Note: A=DDP-4i, B=GLP-1RA, C=Placebo, D=SGLT-2i.Funnel plot of mean difference of SBP Note: A=DDP-4i, B=GLP-1RA, C=Placebo, D=SGLT-2i.Funnel plot of mean difference of LVEF% Note: A=GLP-1RA, B=Placebo, C=SGLT-2i.Funnel plot of mean difference of NT-pro BNP Note: A = GLP-1RA, B = Placebo, C = SGLT-2i.Funnel plot of mean difference of 6MWT Note: A = GLP-1RA, B = Placebo, C = SGLT-2i Note: e’: early diastolic velocity; E/e’: mitral inflow E velocity to tissue doppler e’ ratio; E/A: early diastolic to late diastolic velocities ratio; CVD: cardiovascular disease; DPP-4i: dipeptidyl peptidase-4 inhibitor; GLP-1RA [file 12872_2023_3324_MOESM5_ESM.pdf]

Figure S4 Funnel plot of mean difference

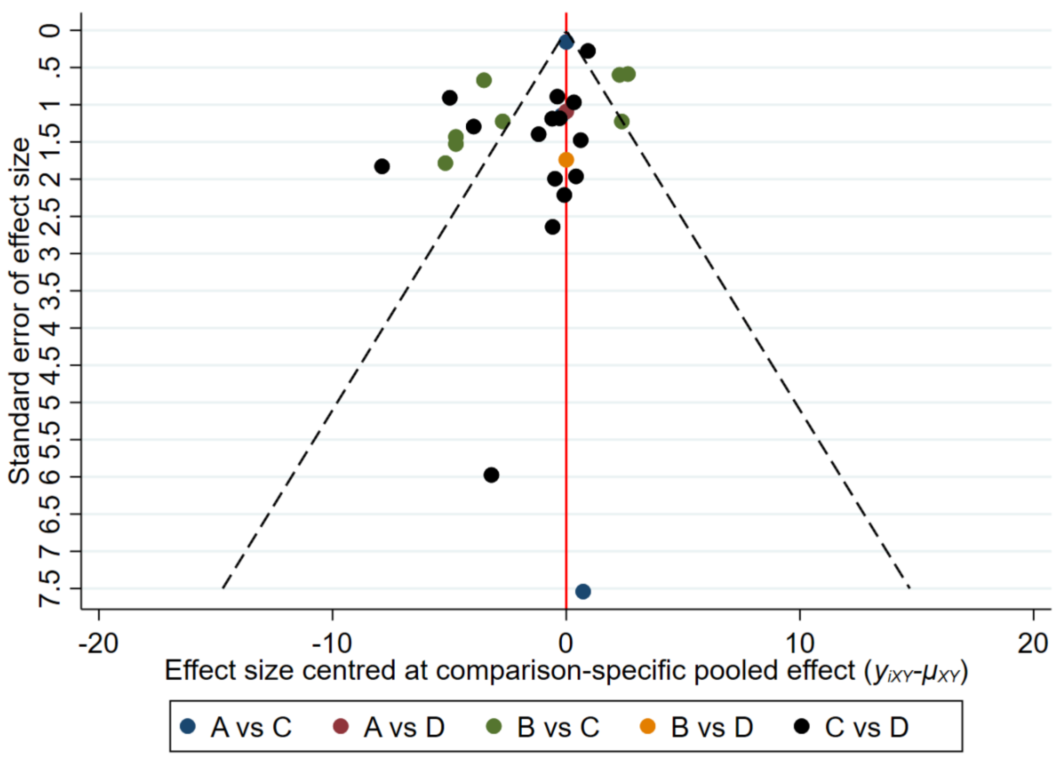

Figure S4(a) Funnel plot of mean difference of LVEF%

Note: A=DDP-4i, B=GLP-1RA, C=Placebo, D=SGLT-2i

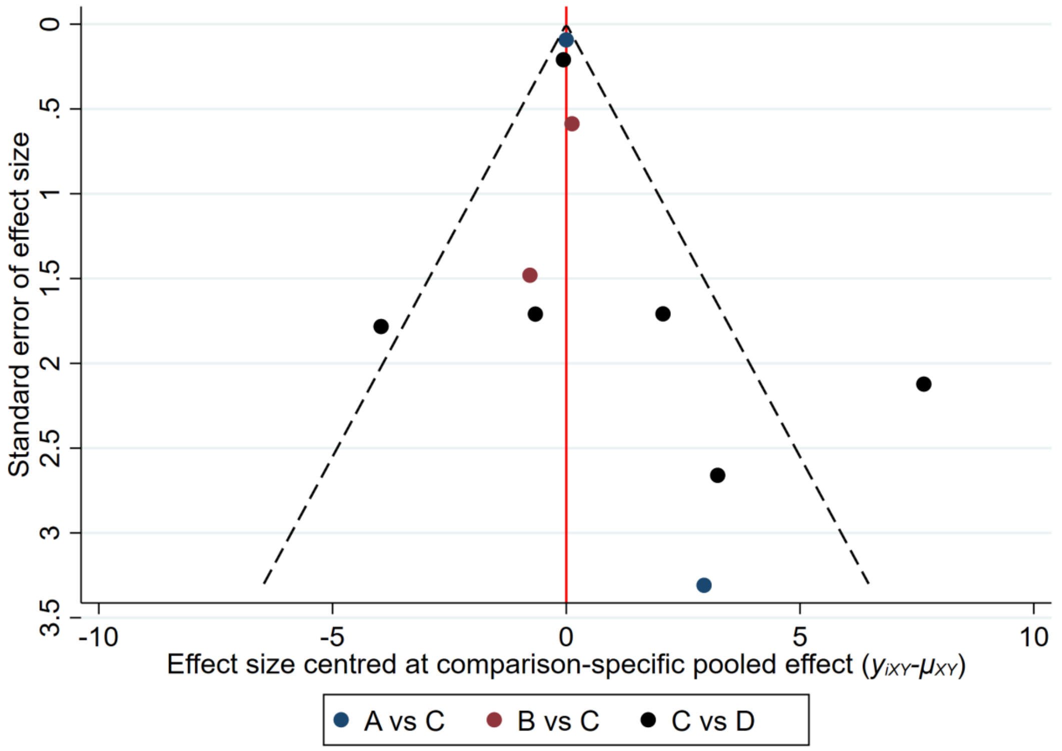

Figure S4(b) Funnel plot of mean difference of LVEDD

Note: A=DDP-4i, B=GLP-1RA, C=Placebo, D=SGLT-2i

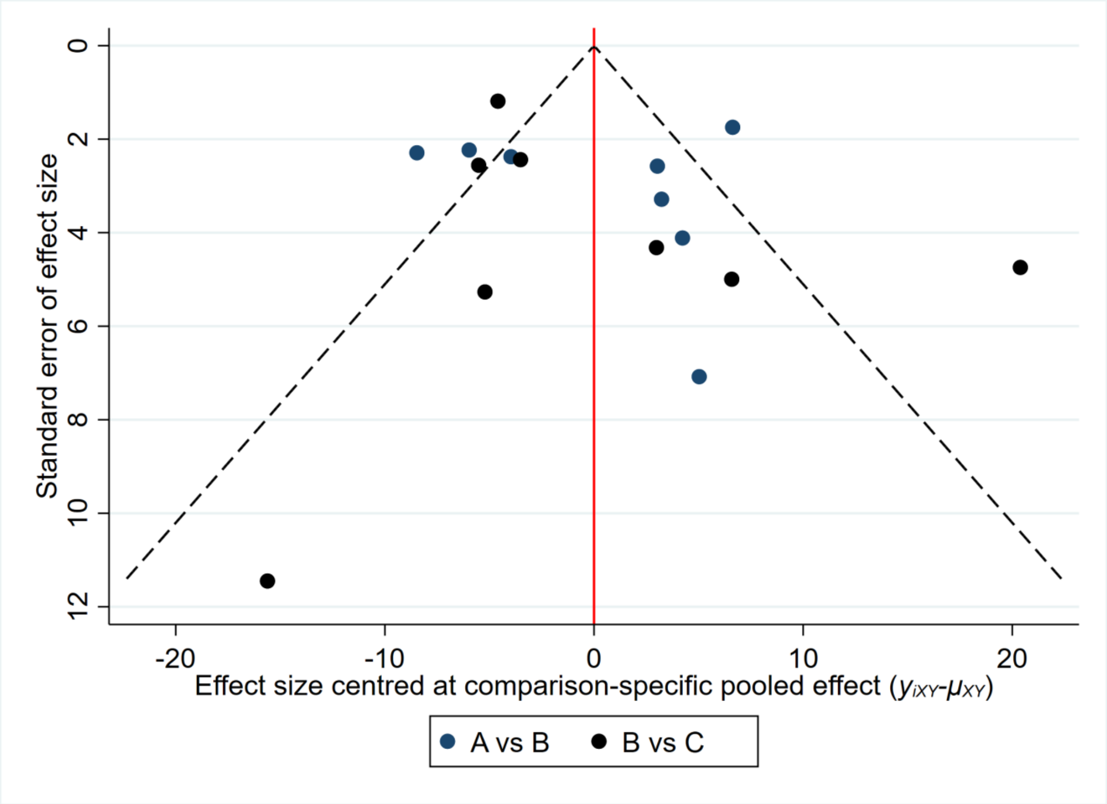

Figure S4(c) Funnel plot of mean difference of LVEDV

Note: A=DDP-4i, B=GLP-1RA, C=Placebo, D=SGLT-2i

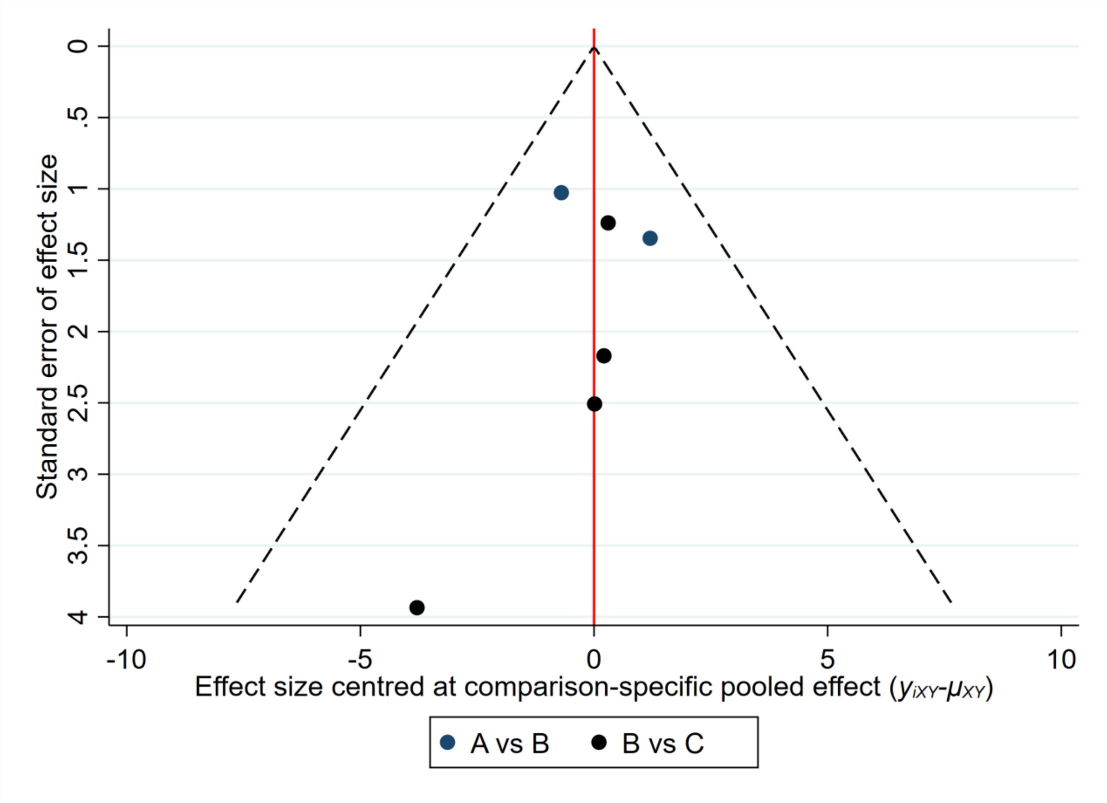

Figure S4(d) Funnel plot of mean difference of LVESD

Note: A=GLP-1RA, B=Placebo, C=SGLT-2i

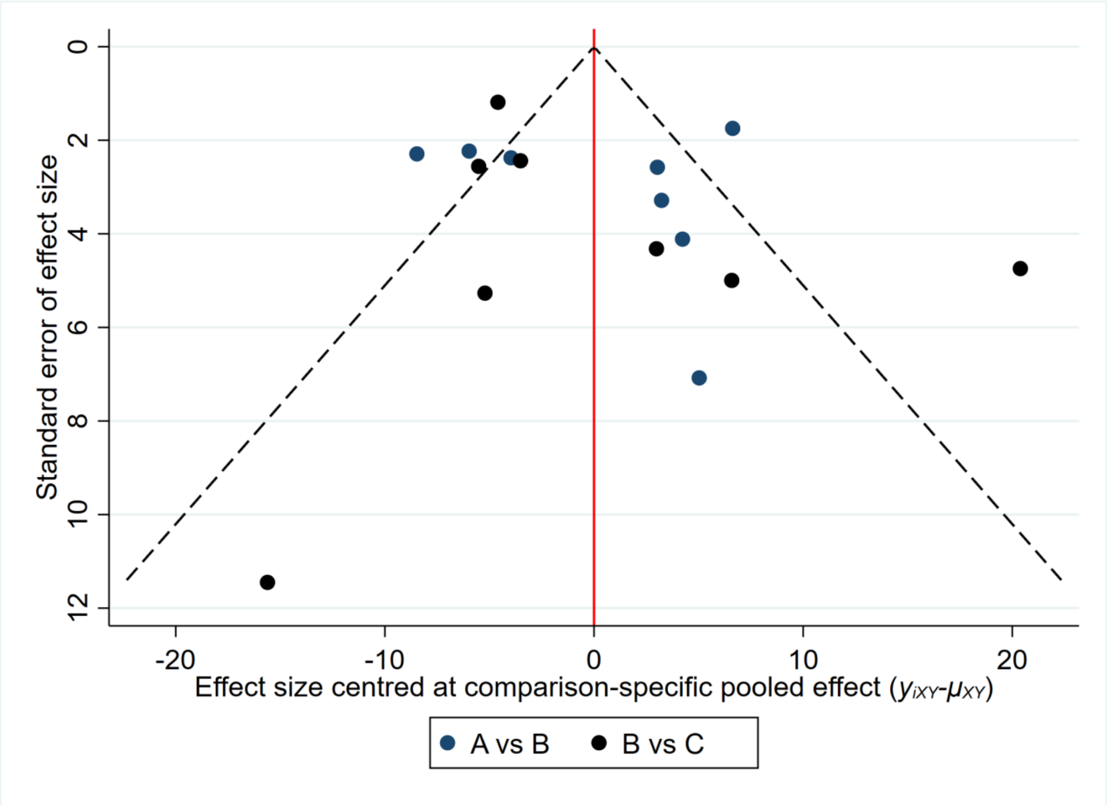

Figure S4(e) Funnel plot of mean difference of LVESV

Note: A=GLP-1RA, B=Placebo, C=SGLT-2i

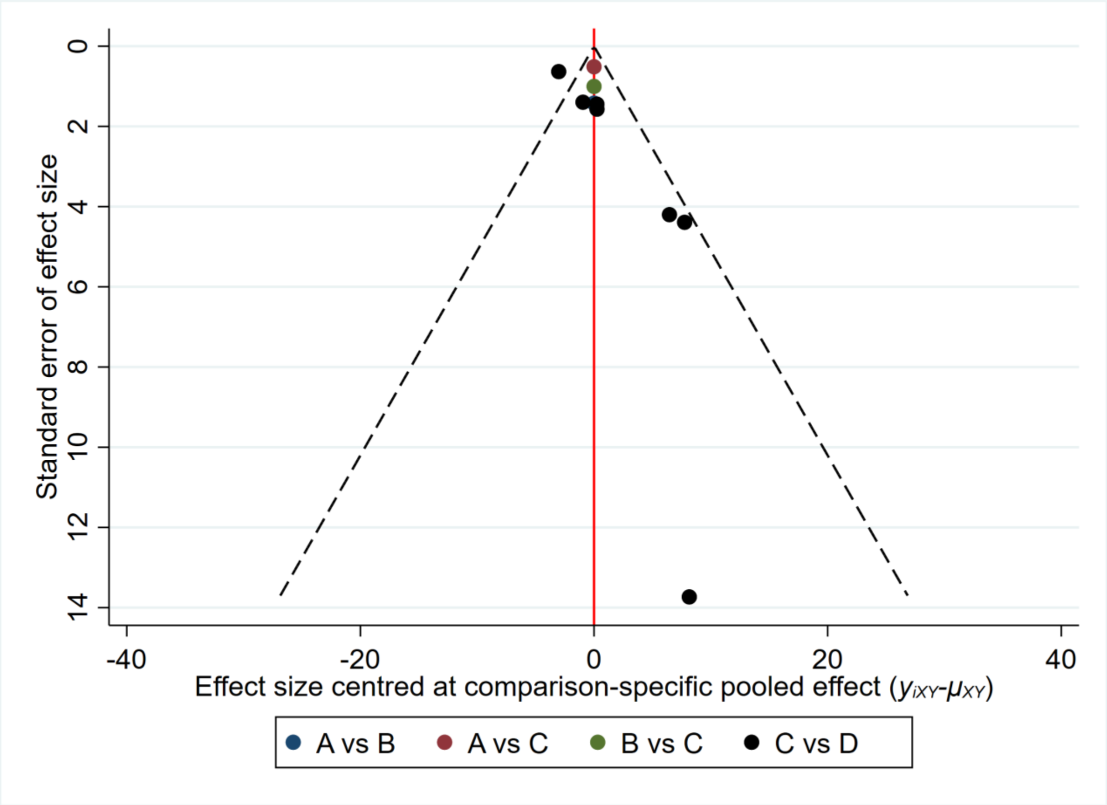

Figure S4(f) Funnel plot of mean difference of LVMI

Note: A=DDP-4i, B=GLP-1RA, C=Placebo, D=SGLT-2i

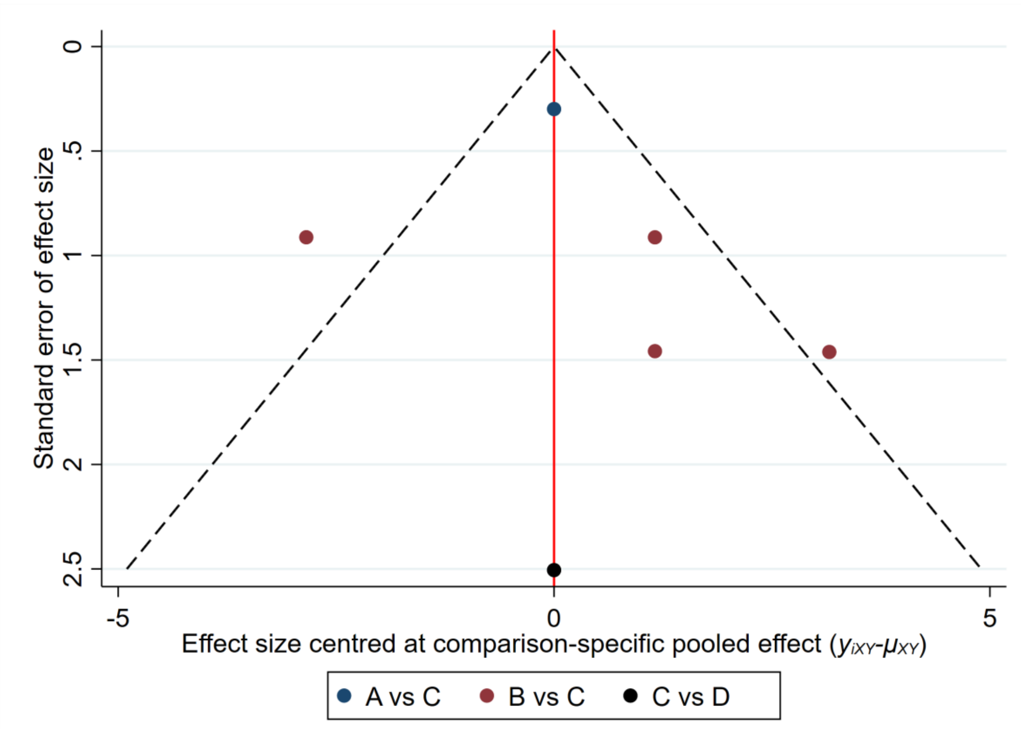

Figure S4(g) Funnel plot of mean difference of e’

Note: A=DDP-4i, B=GLP-1RA, C=Placebo, D=SGLT-2i

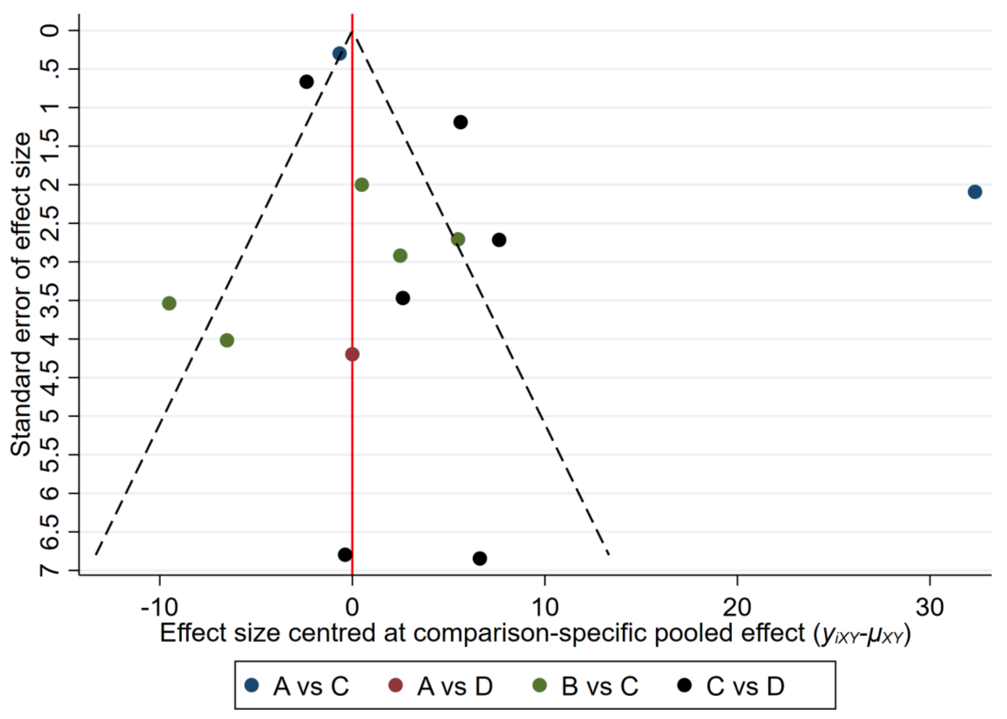

Figure S4(h) Funnel plot of mean difference of E/e’

Note: A=DDP-4i, B=GLP-1RA, C=Placebo, D=SGLT-2i

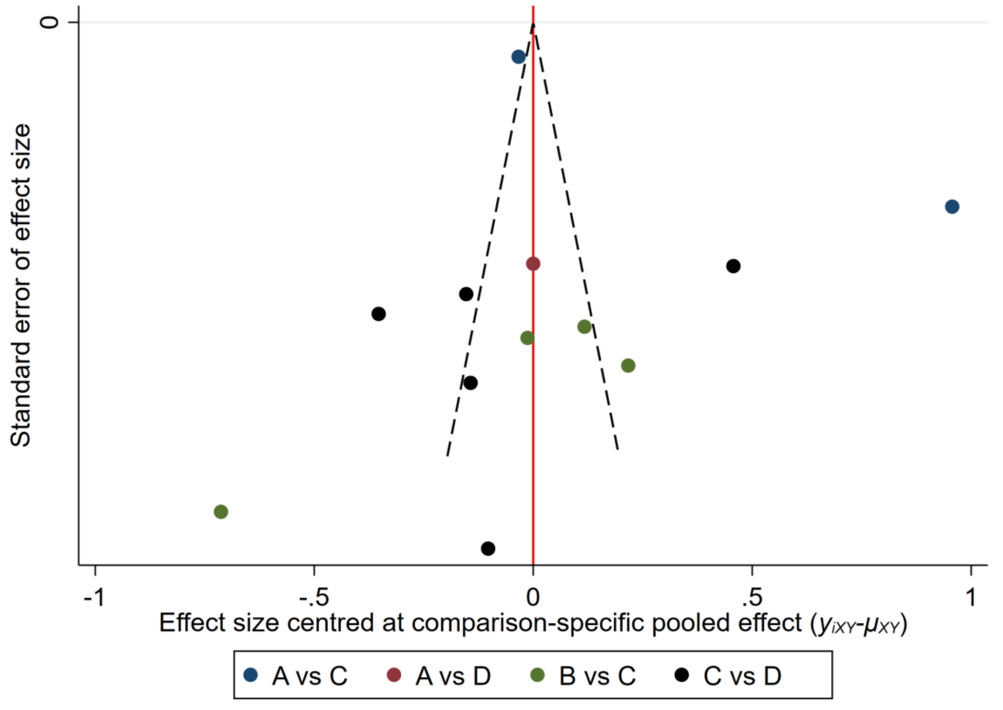

Figure S4(i) Funnel plot of mean difference of E/A

Note: A=DDP-4i, B=GLP-1RA, C=Placebo, D=SGLT-2i

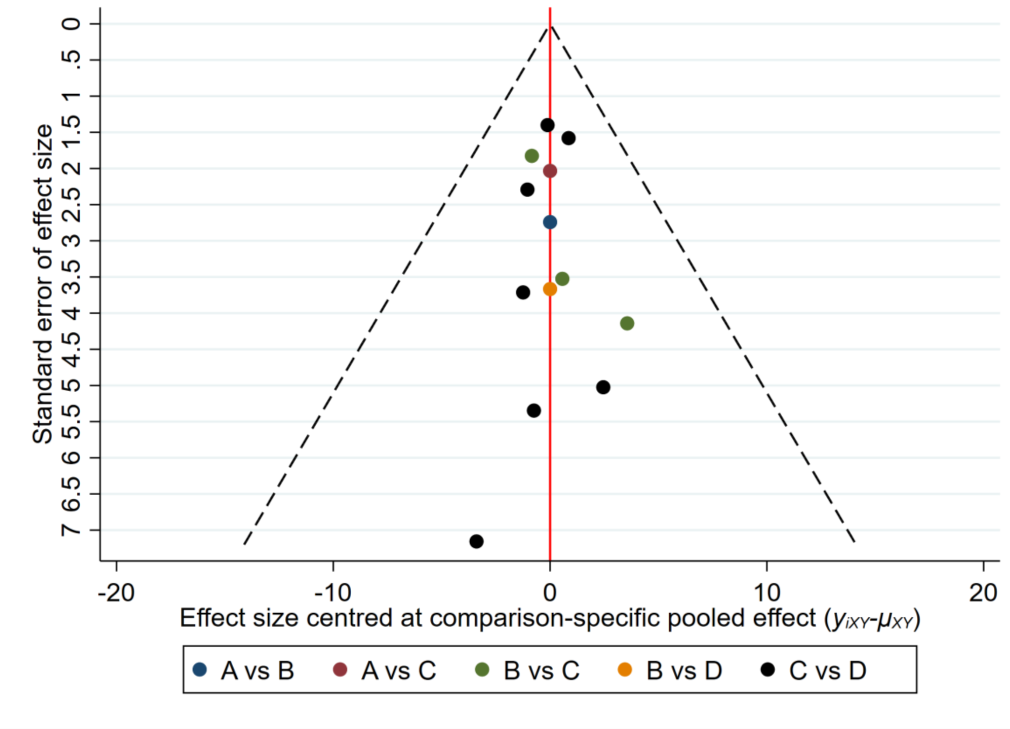

Figure S4(j) Funnel plot of mean difference of SBP

Note: A=DDP-4i, B=GLP-1RA, C=Placebo, D=SGLT-2i

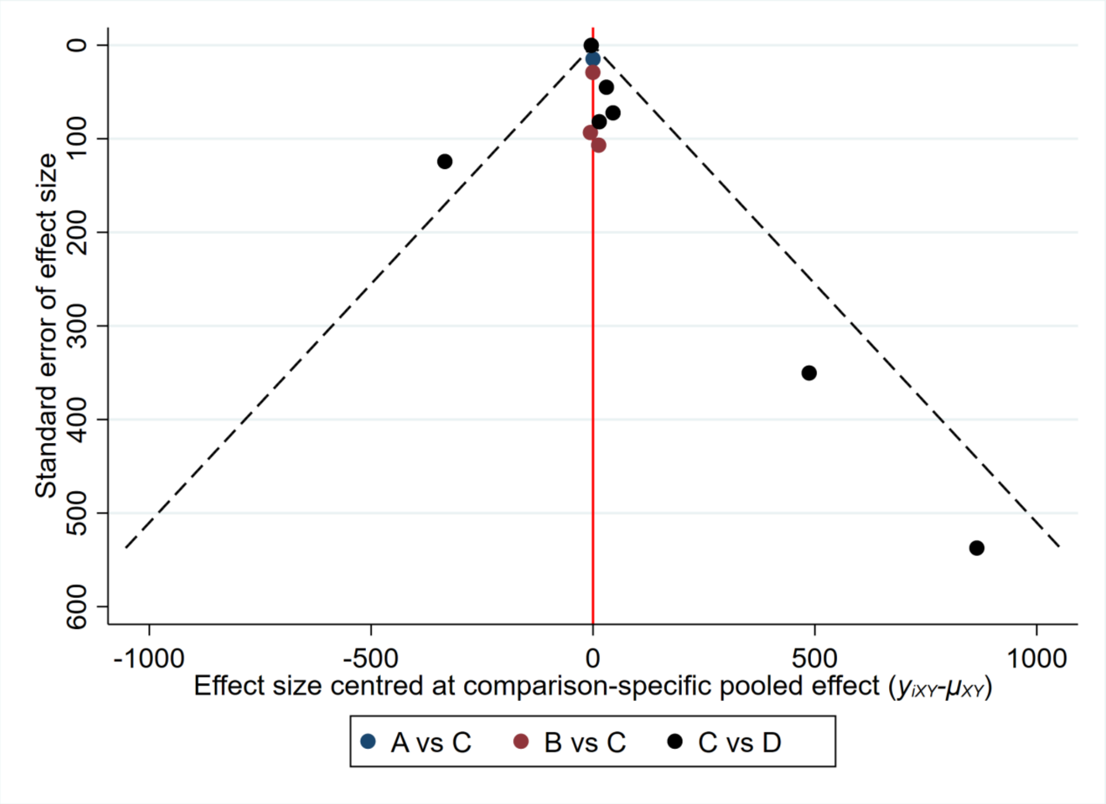

Figure S4(k) Funnel plot of mean difference of NT-pro BNP

Note: A=DDP-4i, B=GLP-1RA, C=Placebo, D=SGLT-2i

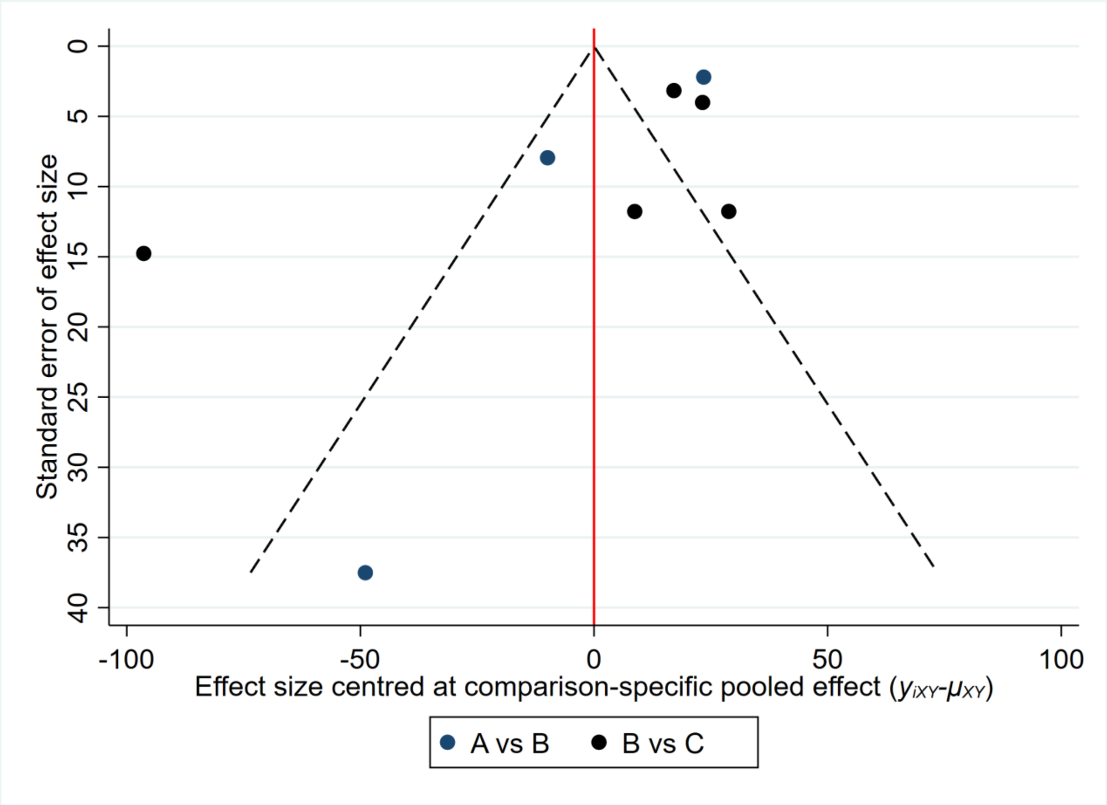

Figure S4(l) Funnel plot of mean difference of 6MWT

Note: A= GLP-1RA, B=Placebo, C=SGLT-2i

Subgroup of patients with T2DM and CVD

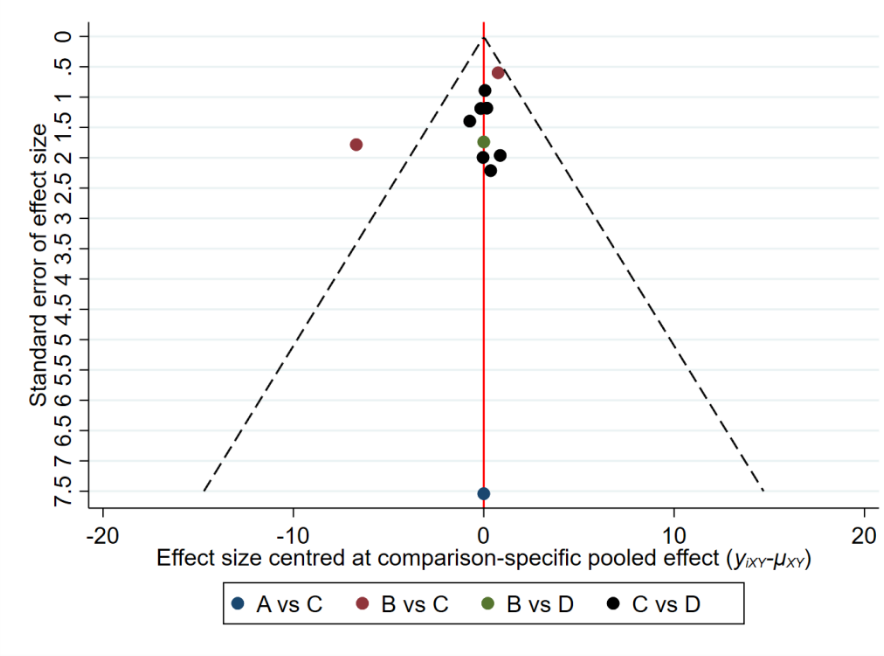

Figure S4(m) Funnel plot of mean difference of LVEF%

Note: A=DDP-4i, B=GLP-1RA, C=Placebo, D=SGLT-2i

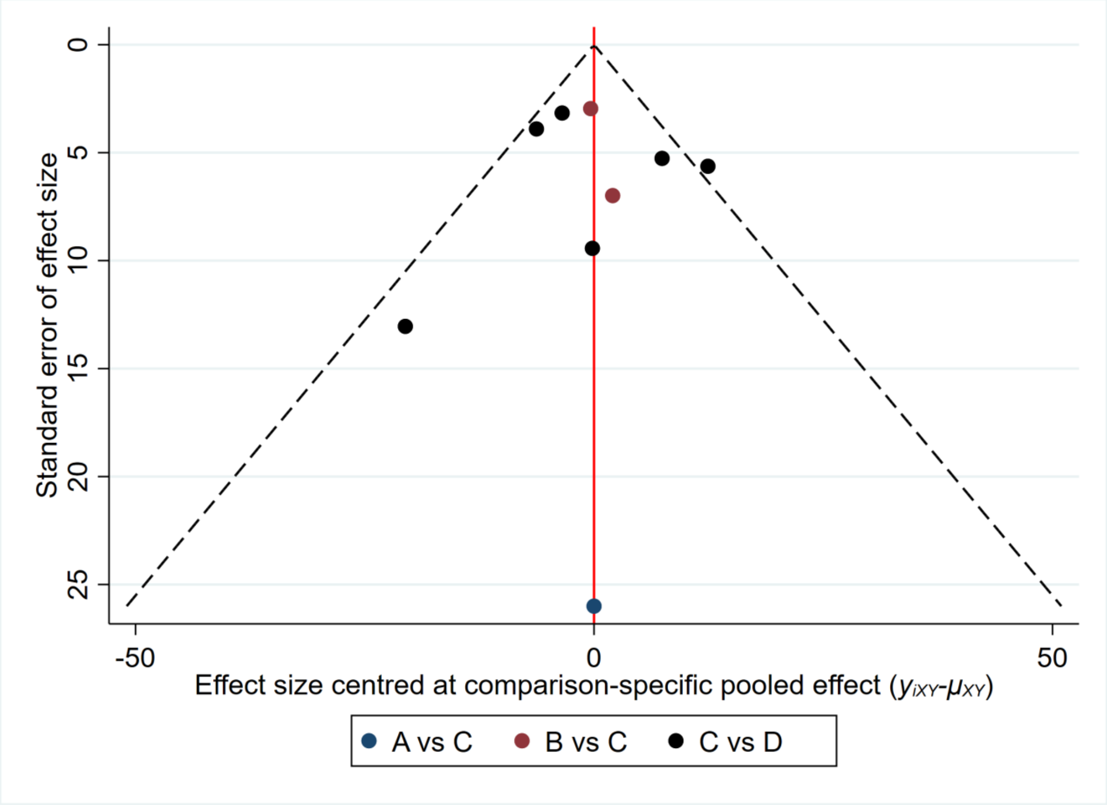

Figure S4(n) Funnel plot of mean difference of LVEDV

Note: A=DDP-4i, B=GLP-1RA, C=Placebo, D=SGLT-2i

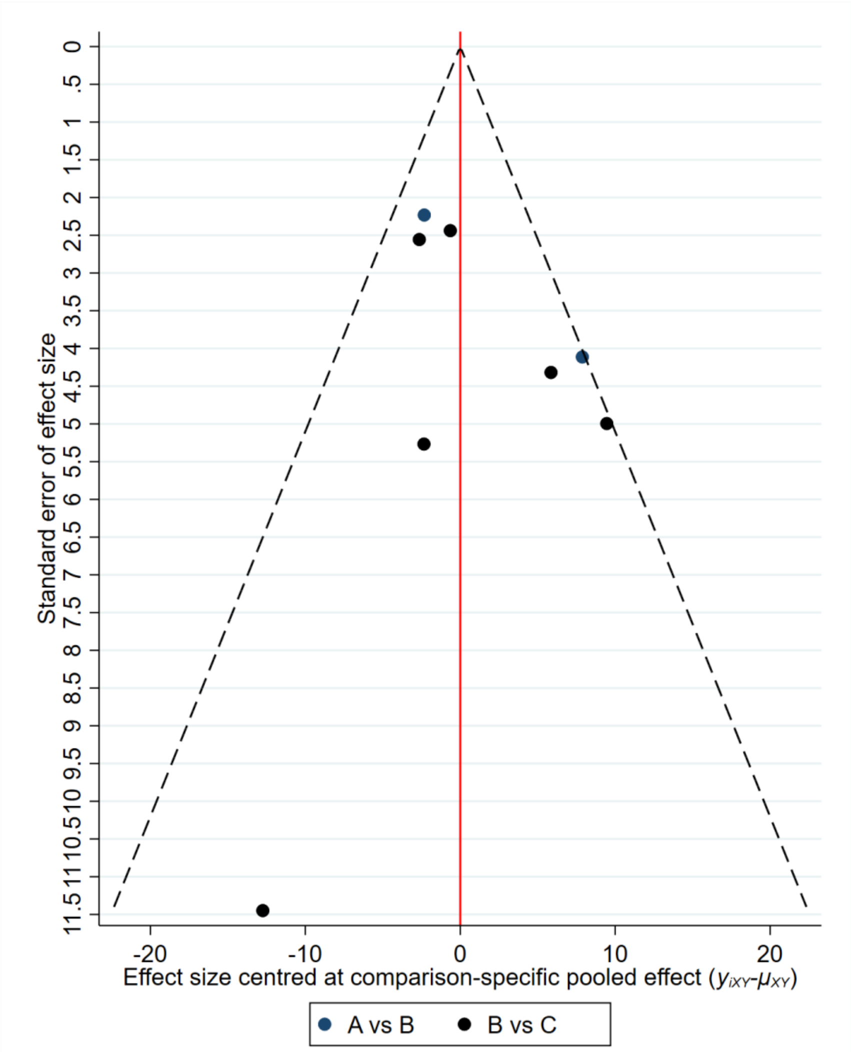

Figure S4(o) Funnel plot of mean difference of LVESV

Note: A=DDP-4i, B=GLP-1RA, C=Placebo, D=SGLT-2i

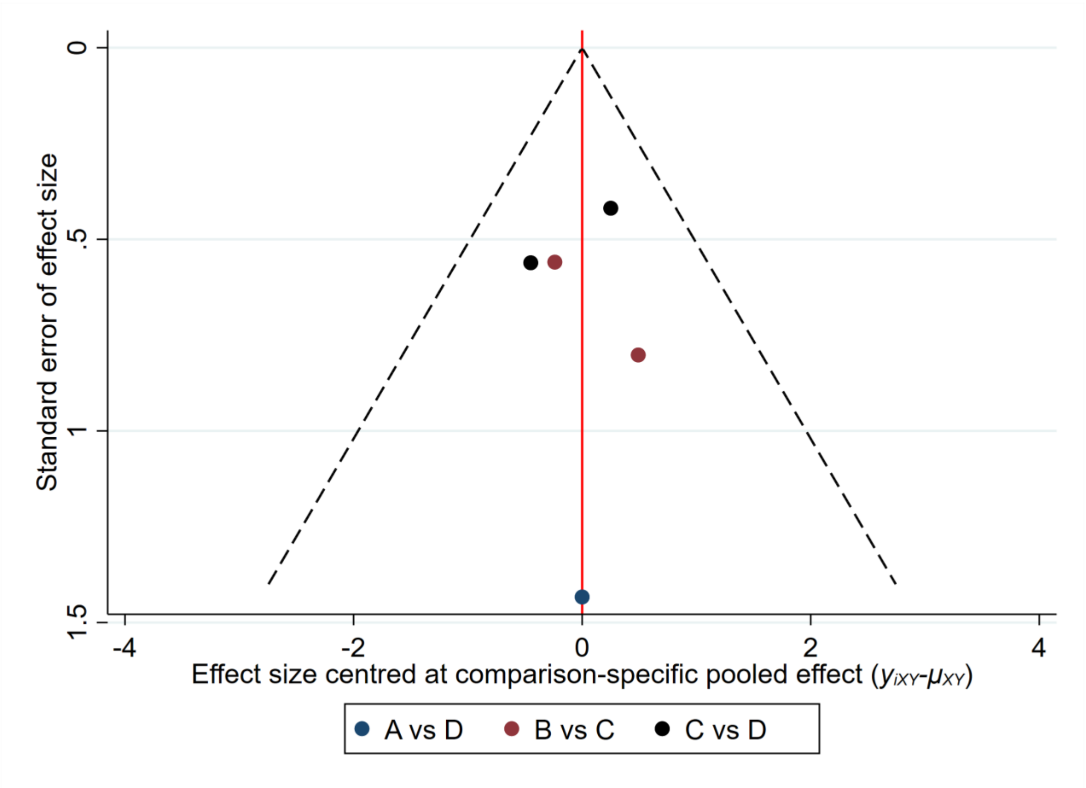

Figure S4(p) Funnel plot of mean difference of E/e’

Note: A=DDP-4i, B=GLP-1RA, C=Placebo, D=SGLT-2i

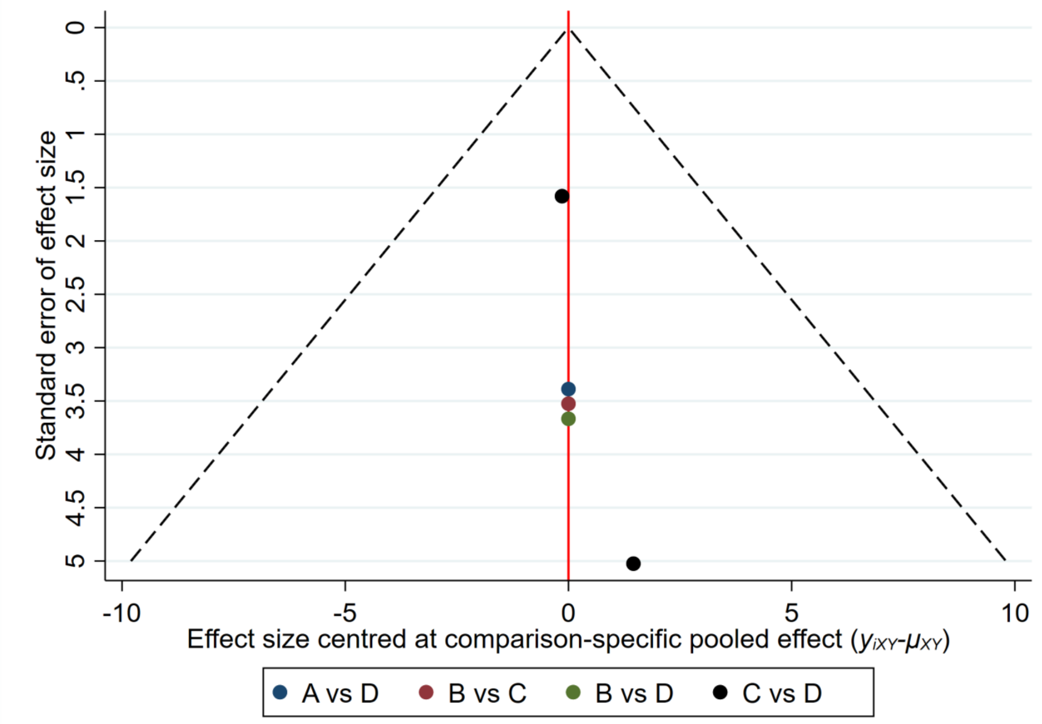

Figure S4(q) Funnel plot of mean difference of SBP

Note: A=DDP-4i, B=GLP-1RA, C=Placebo, D=SGLT-2i

Subgroup of patients with CVD alone

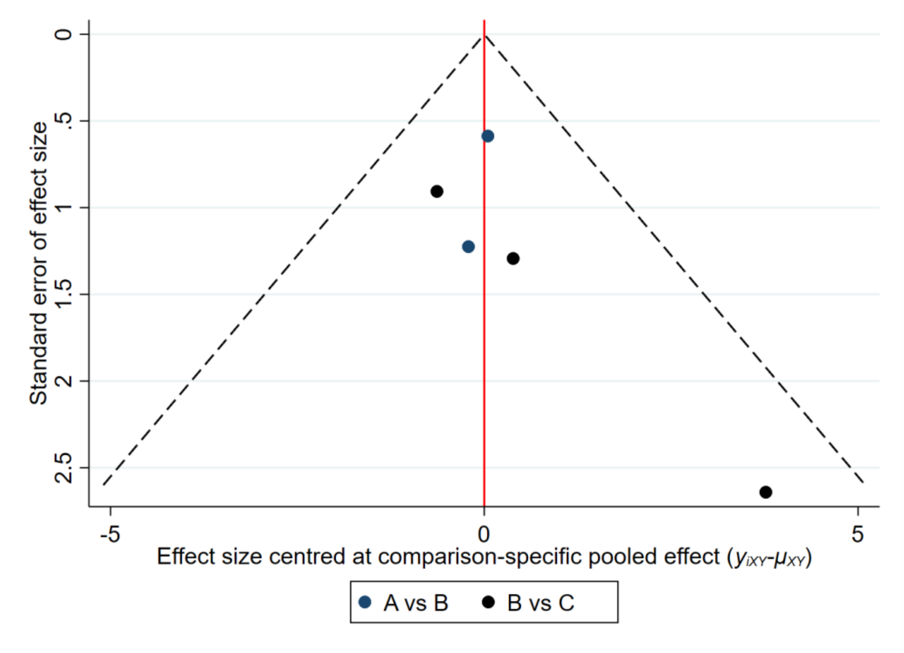

Figure S4(r) Funnel plot of mean difference of LVEF%

Note: A=GLP-1RA, B=Placebo, C=SGLT-2i

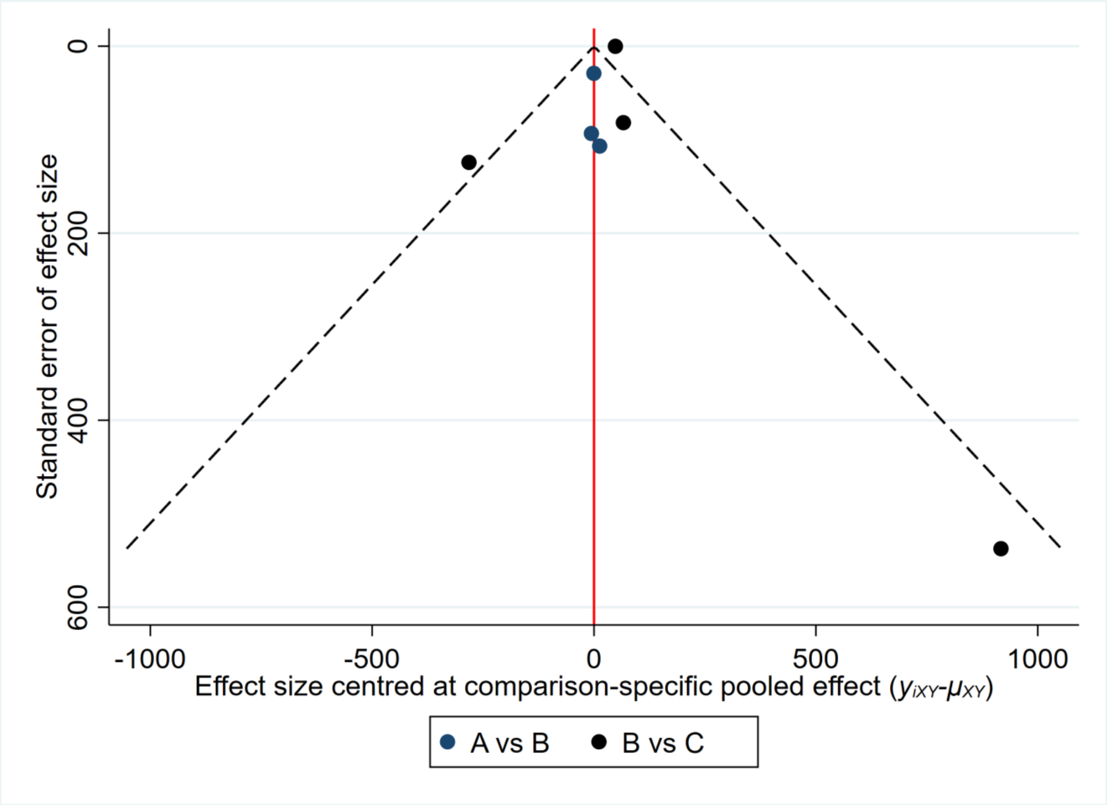

Figure S4(s) Funnel plot of mean difference of NT-pro BNP

Note: A = GLP-1RA, B = Placebo, C = SGLT-2i

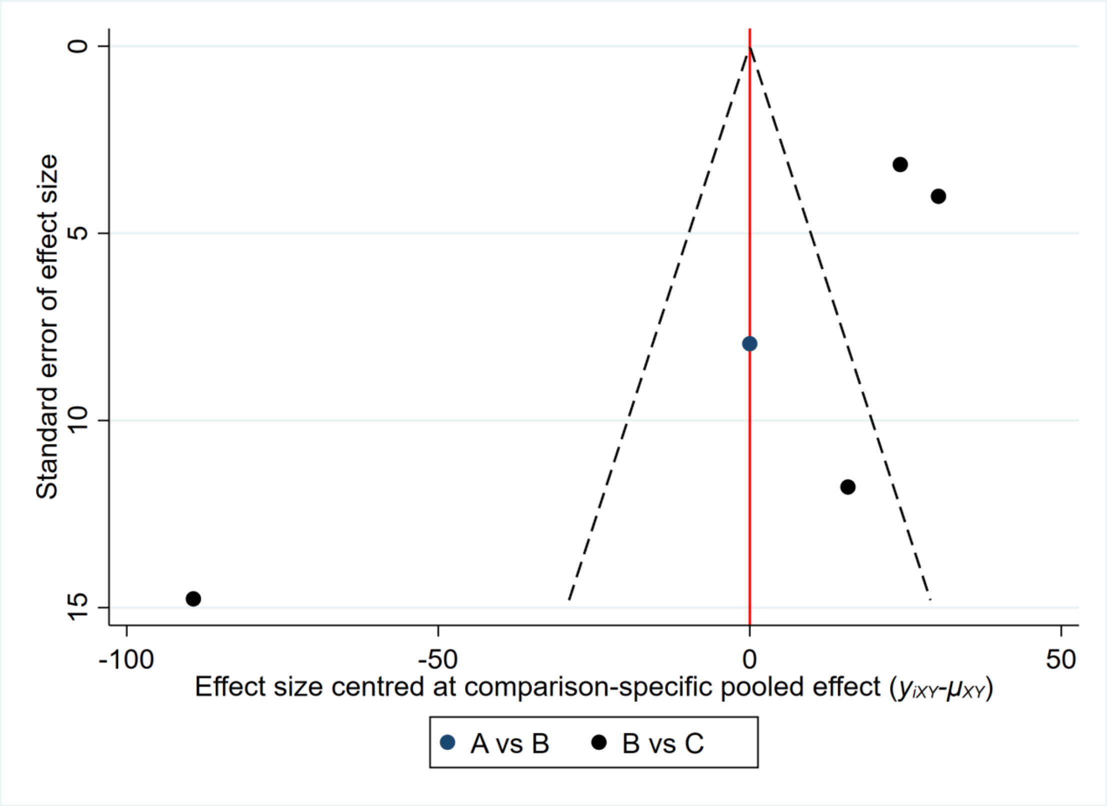

Figure S4(t) Funnel plot of mean difference of 6MWT

Note: A = GLP-1RA, B = Placebo, C = SGLT-2i

Note: e': early diastolic velocity; **E/e'**: mitral inflow E velocity to tissue doppler e' ratio; **E/A**: early diastolic to late diastolic velocities ratio**; CVD**: cardiovascular disease; **DPP-4i**: dipeptidyl peptidase-4 inhibitor; **GLP-1RA**: glucagon-like peptide-1 receptor agonist; **LVEDD**: left ventricular end-diastolic diameter; **LVEDV**: LV end-diastolic volume; **LVEF**: LV ejection fraction; **LVESD**: LV end-systolic diameter; **LVESV**: LV end-systolic volume; **LVMI**: LV mass index; **NT-pro BNP**: immunoreactive amino-terminal pro-brain natriuretic peptide; **SBP**: systolic blood pressure; **SGLT-2i**: sodium glucose cotransporter type 2 inhibitor; **T2DM**: type 2 diabetes; **6MWT**: 6-min walk test.
