# Supplementary material for: Effects of new hypoglycemic drugs on cardiac remodeling: a systematic review and network meta-analysis
Source: BMC Cardiovasc Disord. 2023 Jun 9;23:293. doi: 10.1186/s12872-023-03324-6 (PMC10251583; doi:10.1186/s12872-023-03324-6)
Supplement: Supplementary file 6 — Additional file 6: Figure S5. Forest plot of mean difference.Forest plot of mean difference of LVEF%.Forest plot of mean difference of LVEDD.Forest plot of mean difference of LVEDV.Forest plot of mean difference of LVESD.Forest plot of mean difference of LVESV.Forest plot of mean difference of LVM.Forest plot of mean difference of e’.Forest plot of mean difference of E/e’.Forest plot of mean difference of E/A.Forest plot of mean difference of SBP.Forest plot of mean difference of NT-pro BNP.Forest plot of mean difference of 6MWT.Forest plot of mean difference of LVEF%.Forest plot of mean difference of LVEDV.Forest plot of mean difference of LVESV.Forest plot of mean difference of E/e’.Forest plot of mean difference of SBP.Forest plot of mean difference of LVEF%.Forest plot of mean difference of NT-pro BNP.Forest plot of mean difference of 6MWT. Note: e’: early diastolic velocity; E/e’: mitral inflow E velocity to tissue doppler e’ ratio; E/A: early diastolic to late diastolic velocities ratio; CVD: cardiovascular disease; DPP-4i: dipeptidyl peptidase-4 inhibitor; GLP-1RA: glucagon-like peptide-1 receptor agonist; LVEDD: left ventricular end-diastolic diameter; LVEDV: LV end-diastolic volume; LVEF: LV ejection fraction; LVESD: LV end-systolic diameter; LVESV: LV end-systolic volume; LVMI: LV mass index; NT-pro BNP: immunoreactive amino-terminal pro-brain natriuretic peptide; SBP: systolic blood pressure; SGLT-2i: sodium glucose cotransporter type 2 inhibitor; T2DM: type 2 diabetes; 6MWT: 6-min walk test. [file 12872_2023_3324_MOESM6_ESM.pdf]

FigureS5 Forest plot of mean difference

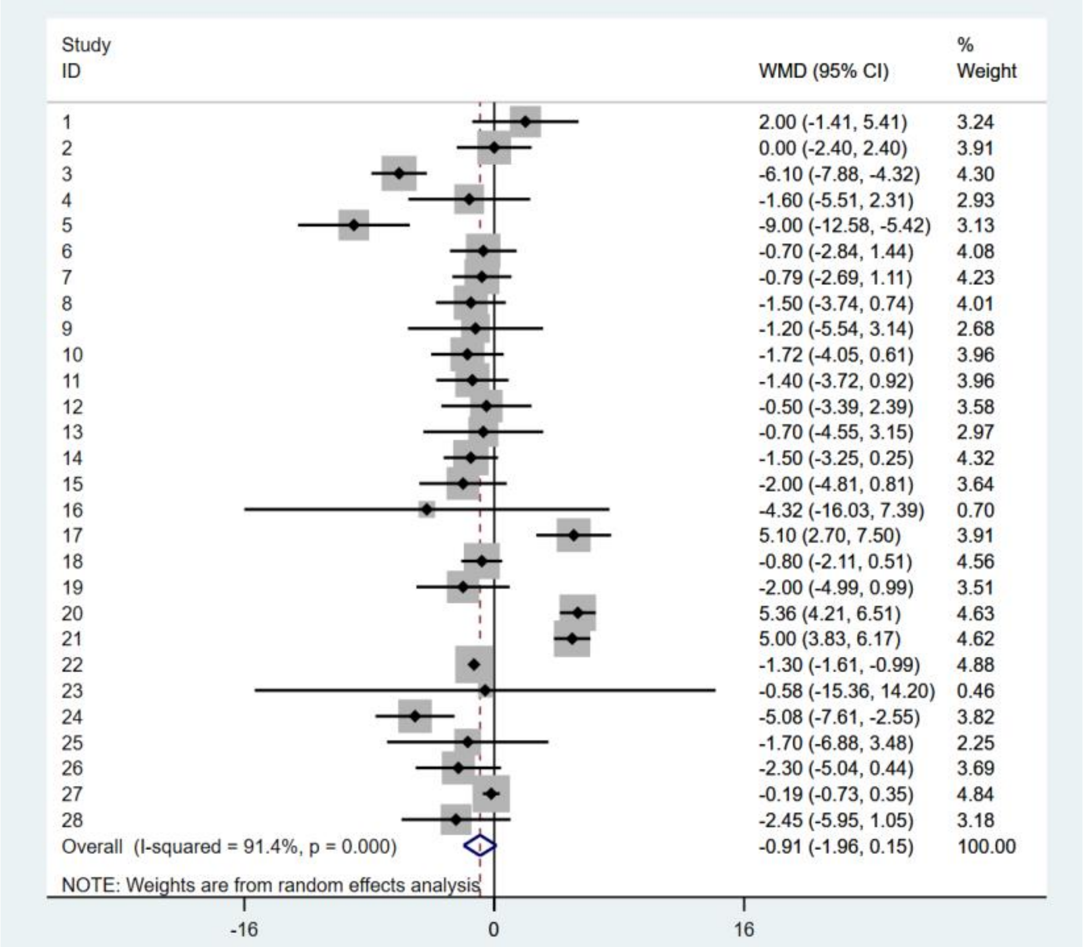

FigureS5(a) Forest plot of mean difference of LVEF%

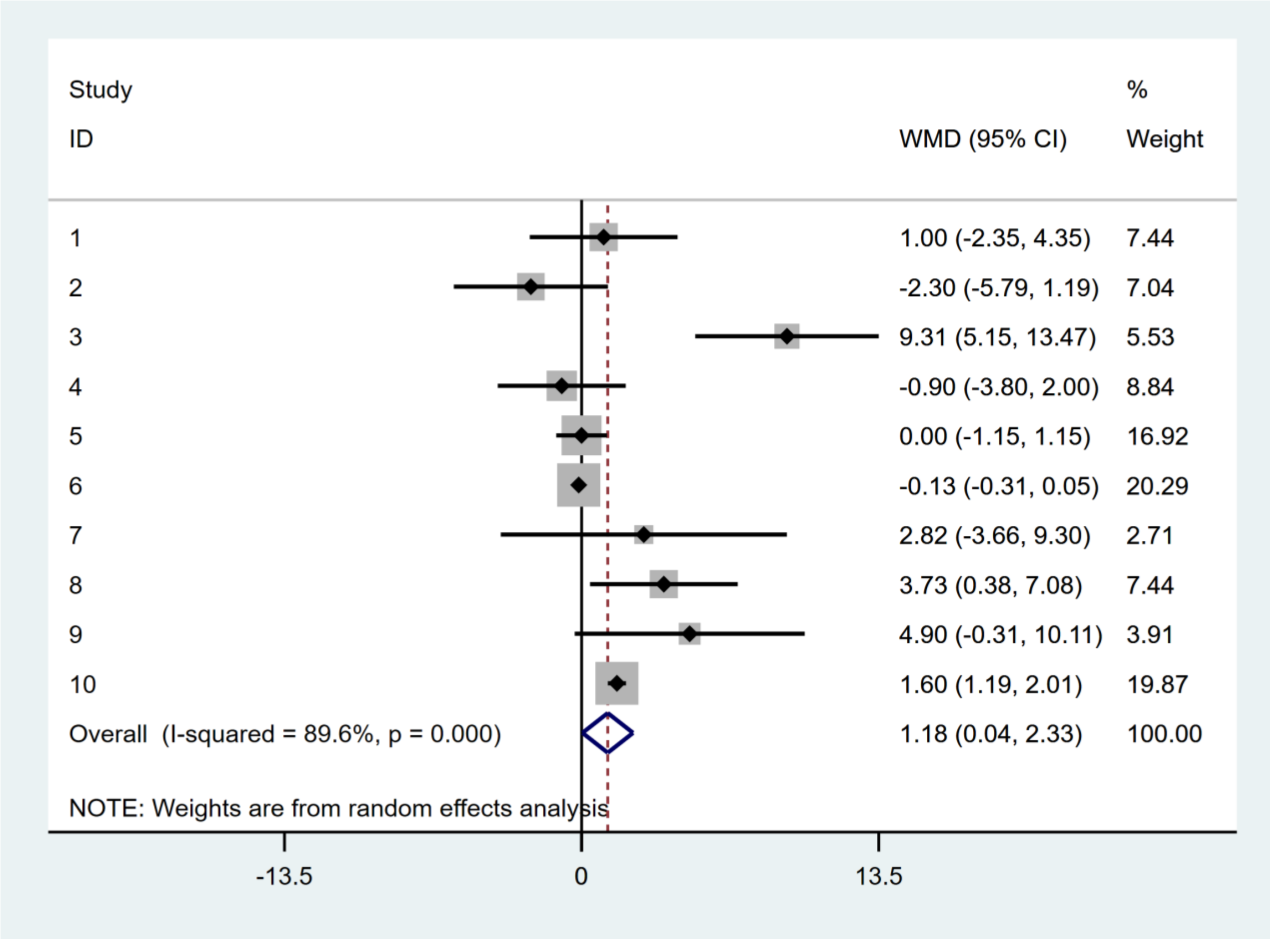

FigureS5(b) Forest plot of mean difference of LVEDD

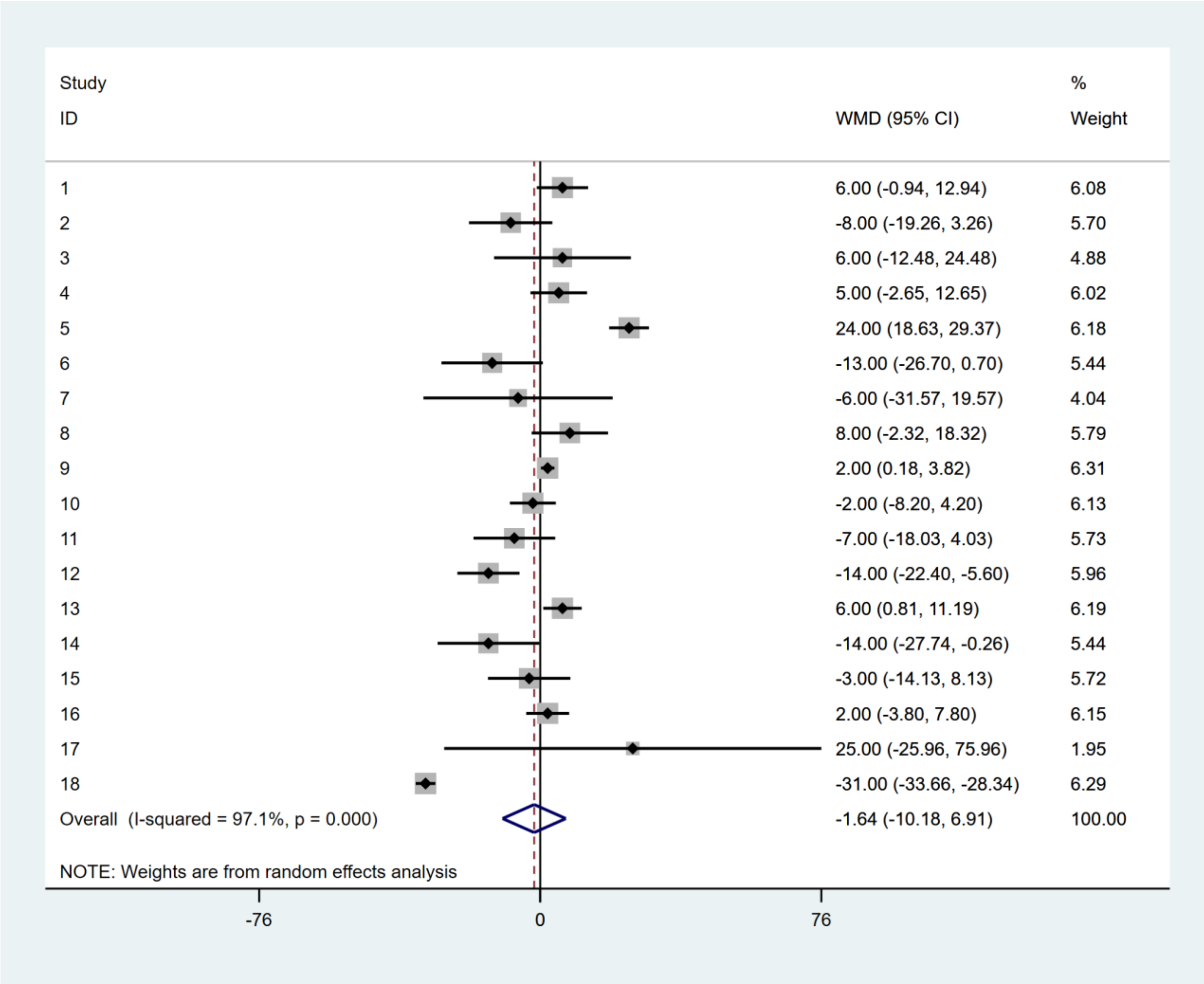

FigureS5(c) Forest plot of mean difference of LVEDV

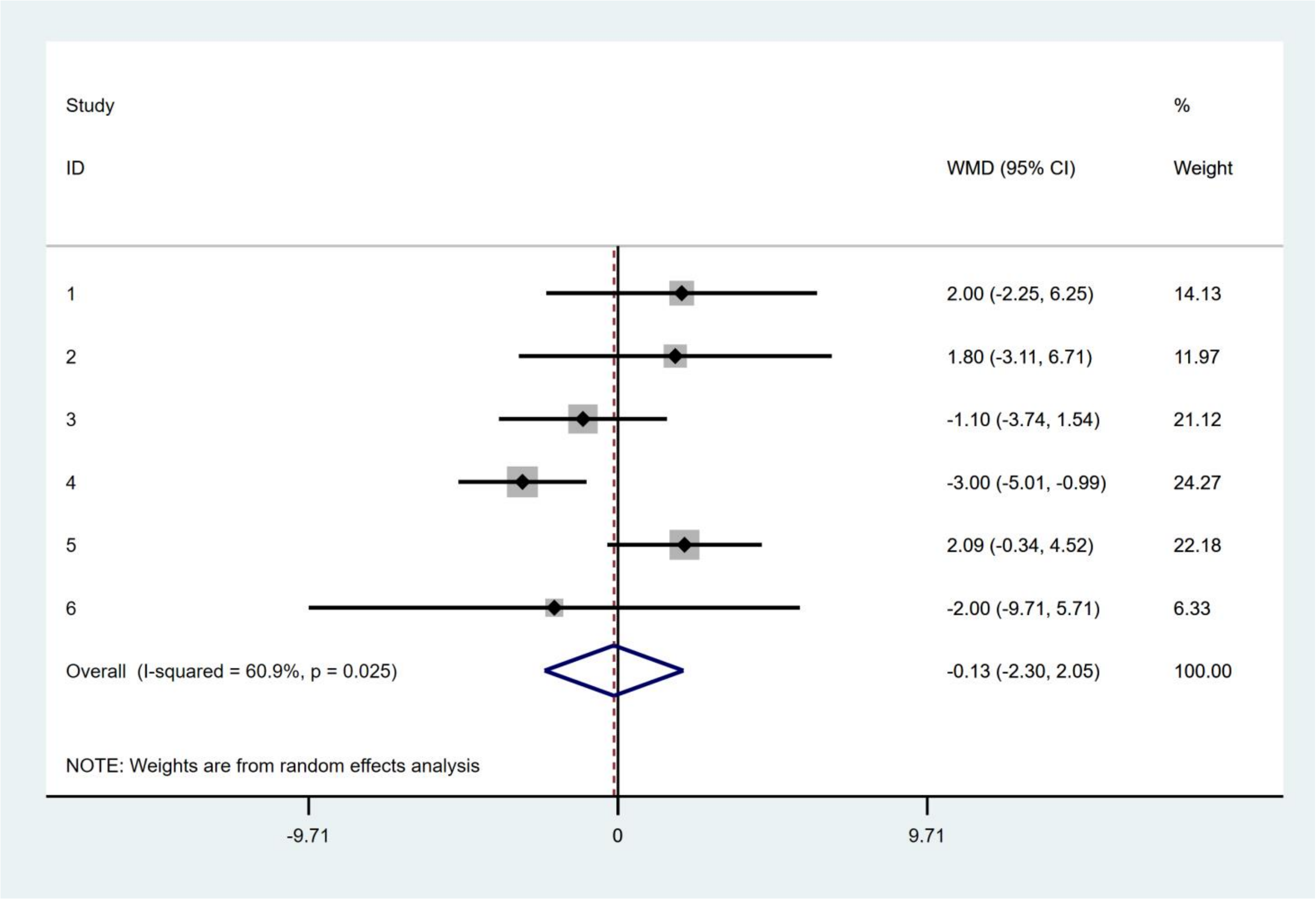

FigureS5(d) Forest plot of mean difference of LVESD

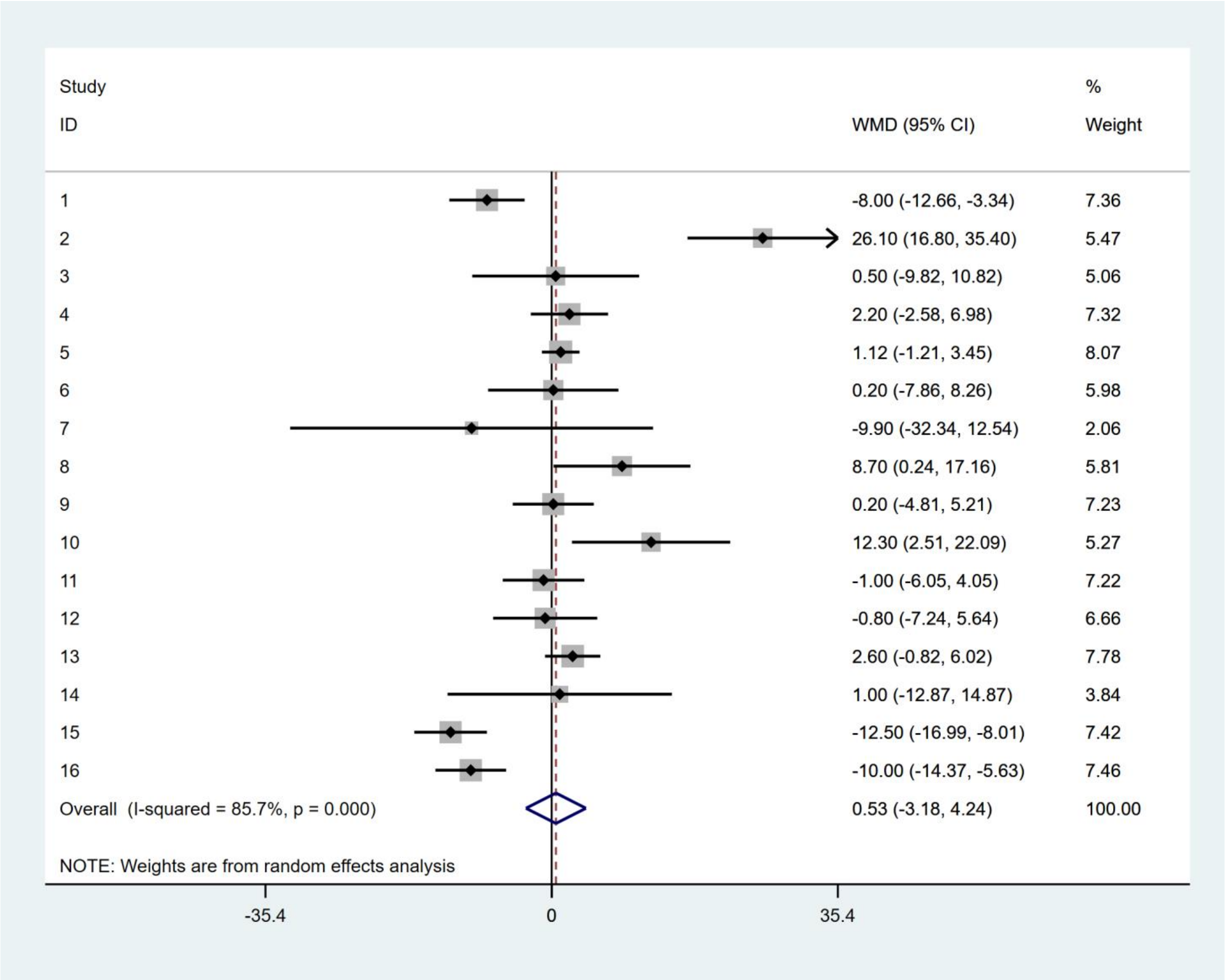

FigureS5(e) Forest plot of mean difference of LVESV

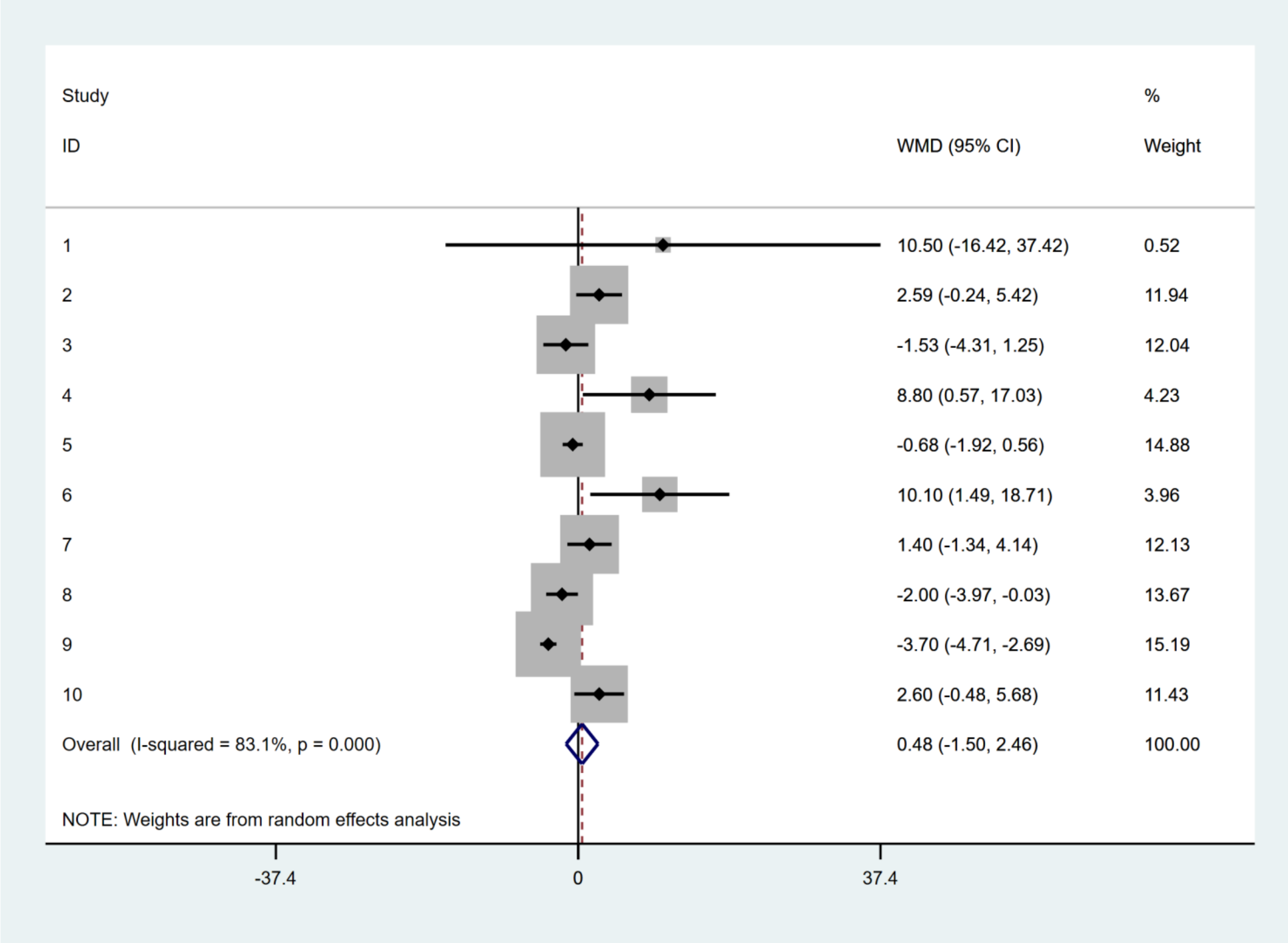

FigureS5(f) Forest plot of mean difference of LVM

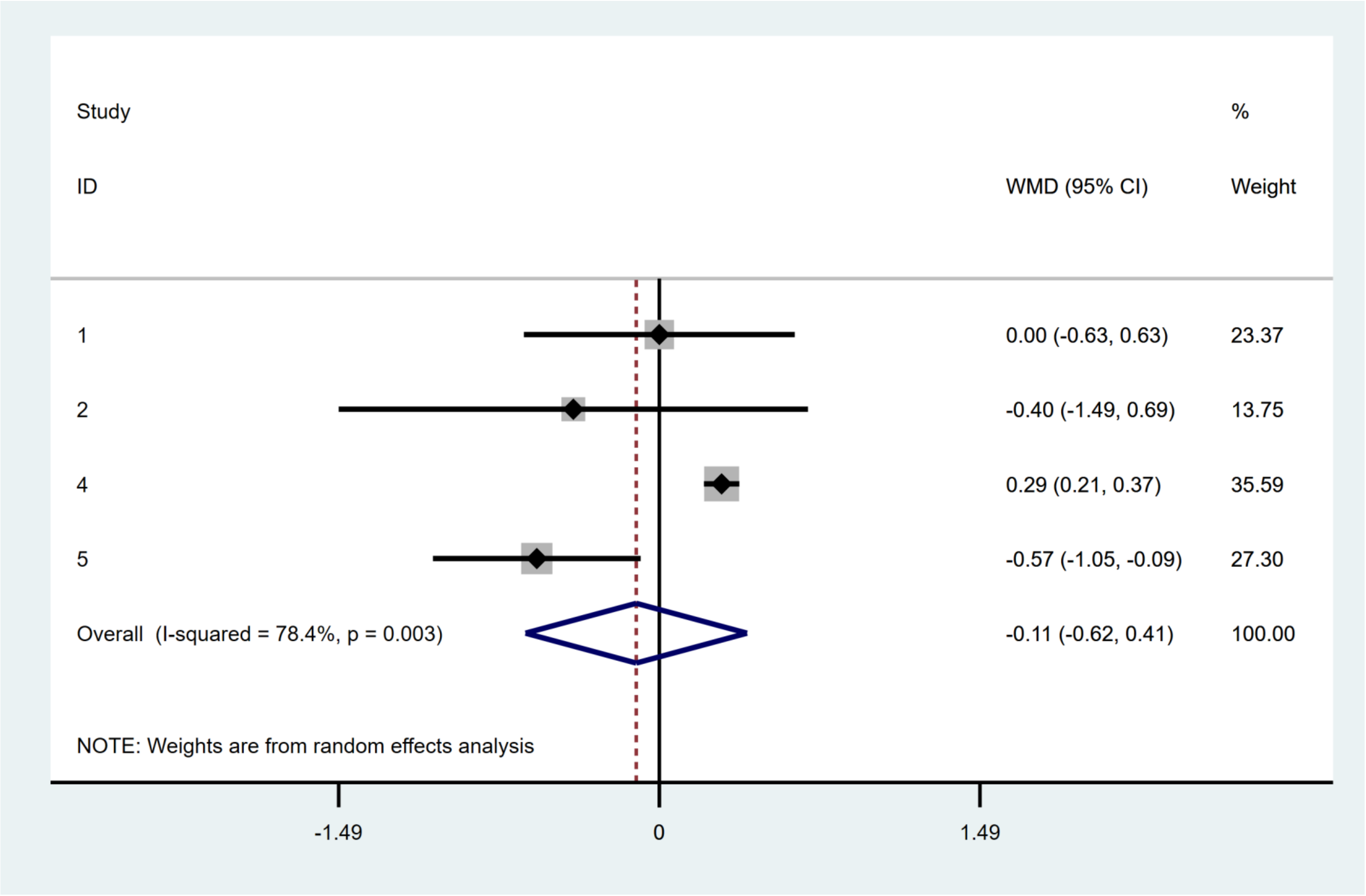

FigureS5(g) Forest plot of mean difference of e’

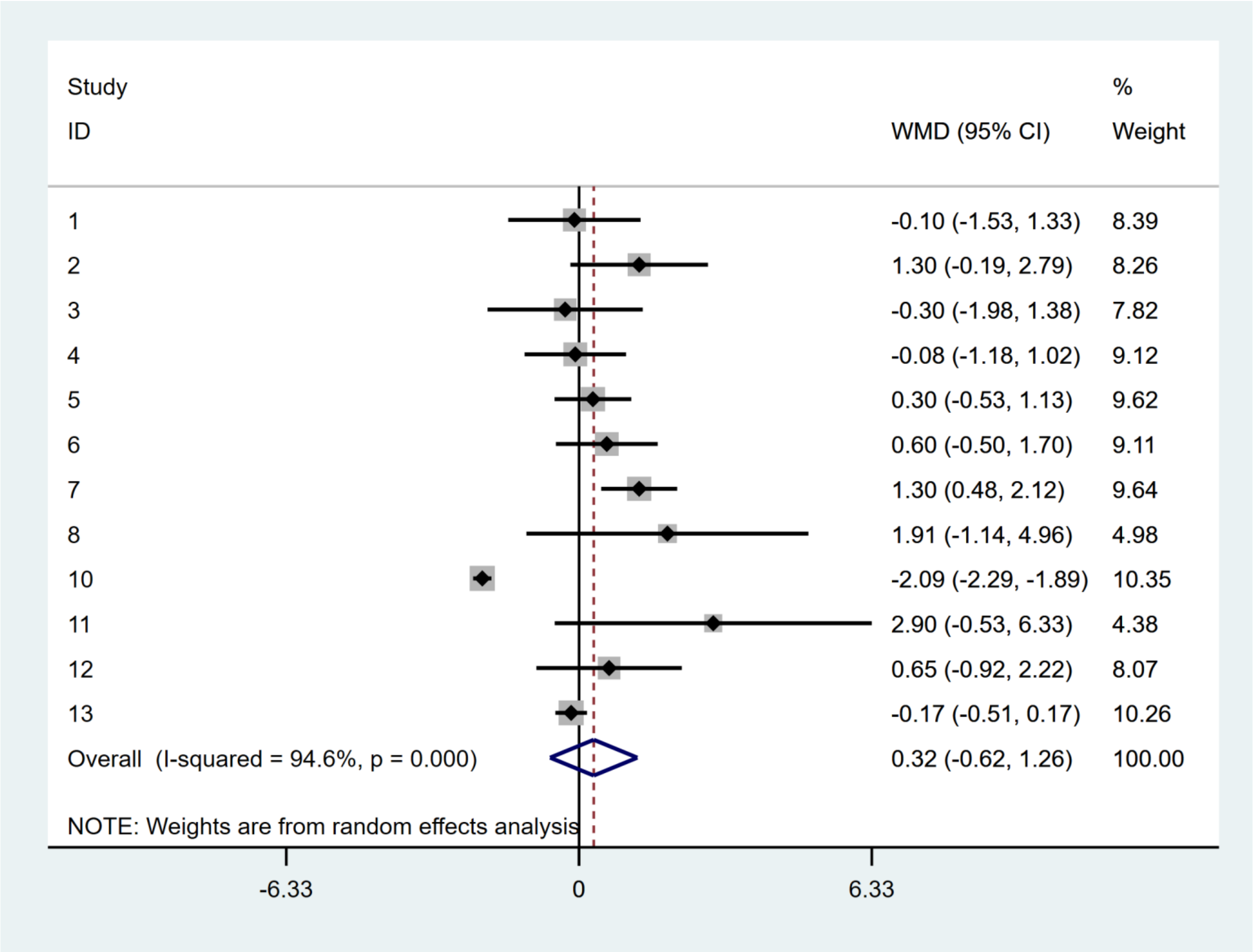

FigureS5(h) Forest plot of mean difference of E/e’

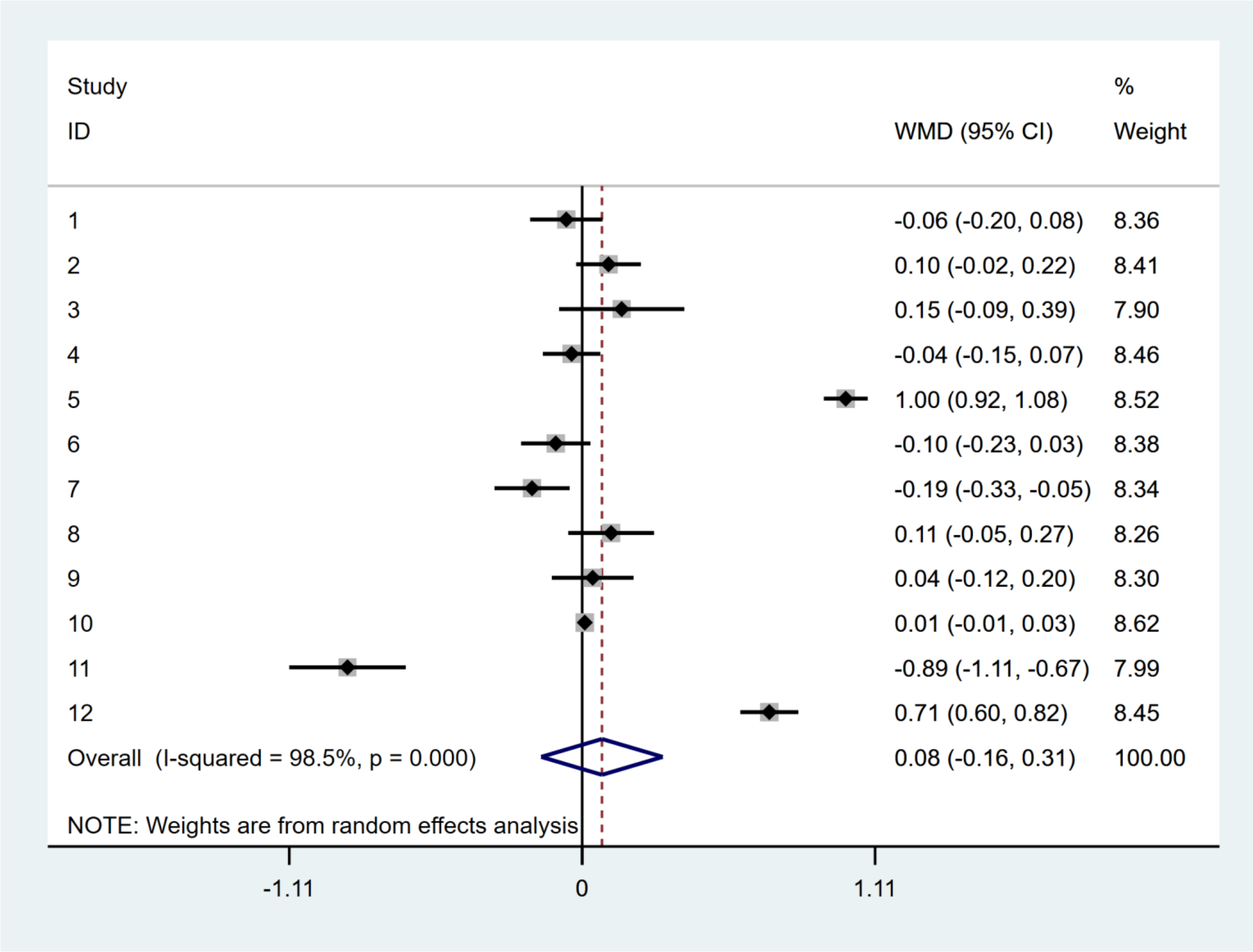

FigureS5(i) Forest plot of mean difference of E/A

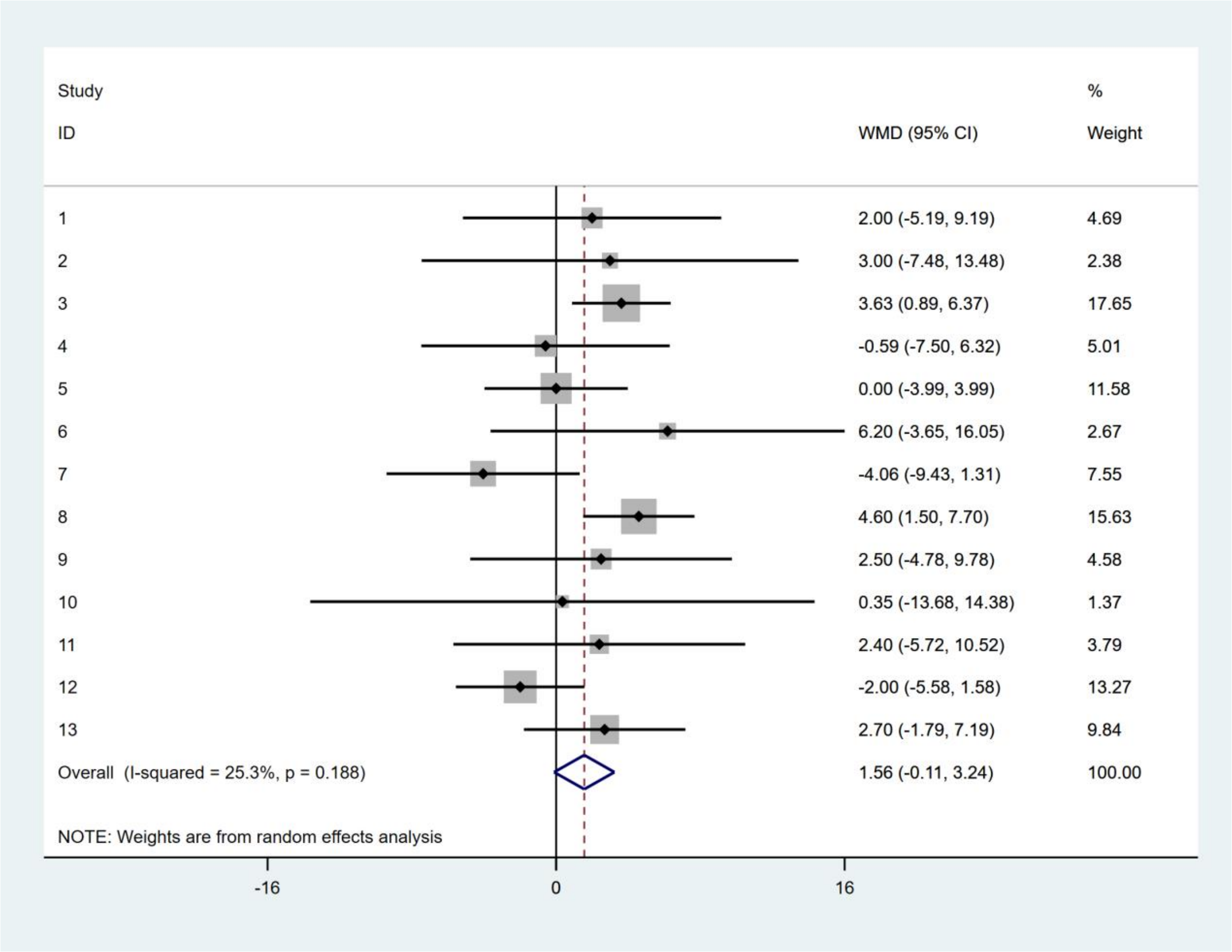

FigureS5(j) Forest plot of mean difference of SBP

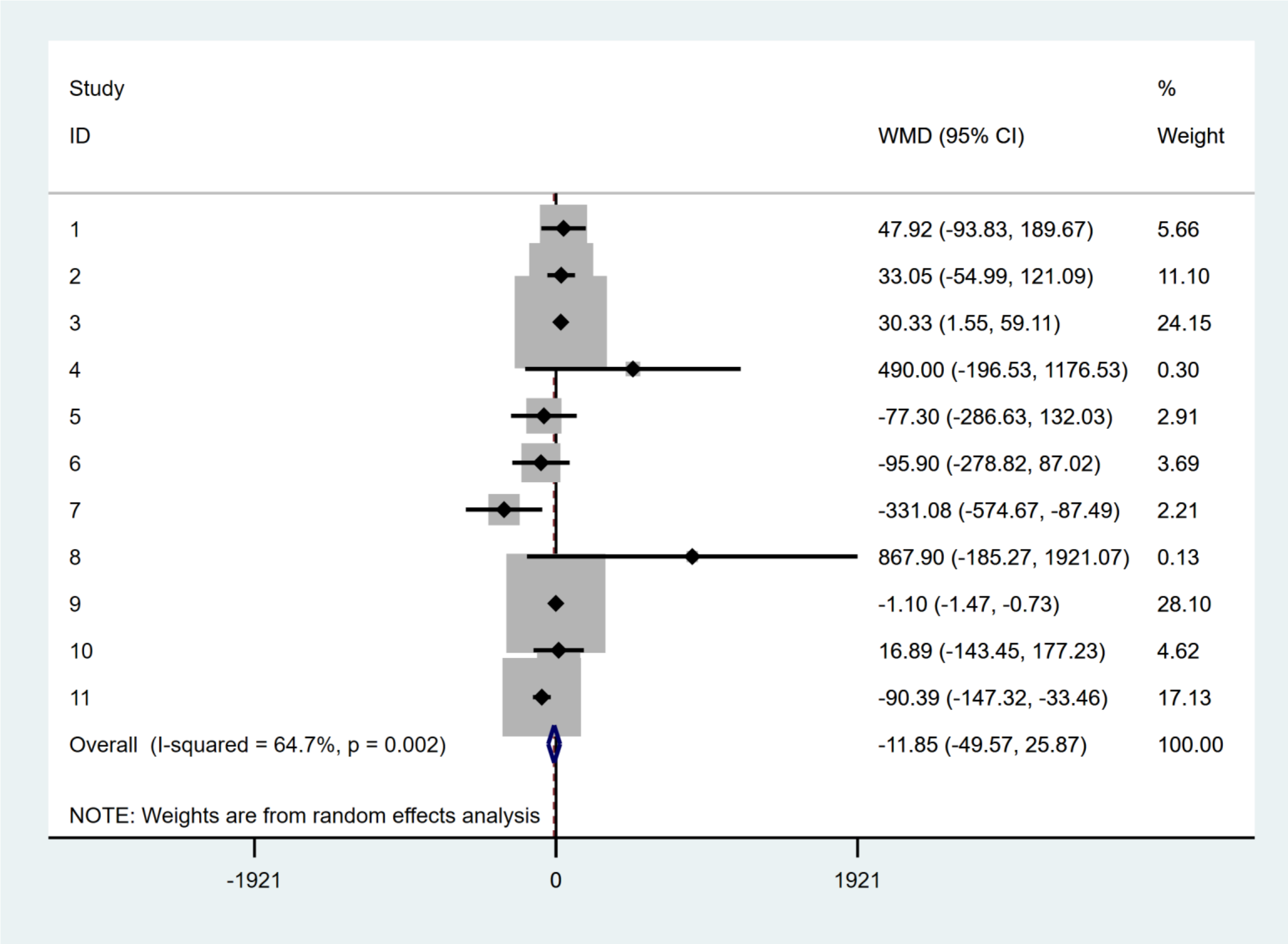

FigureS5(k) Forest plot of mean difference of NT-pro BNP

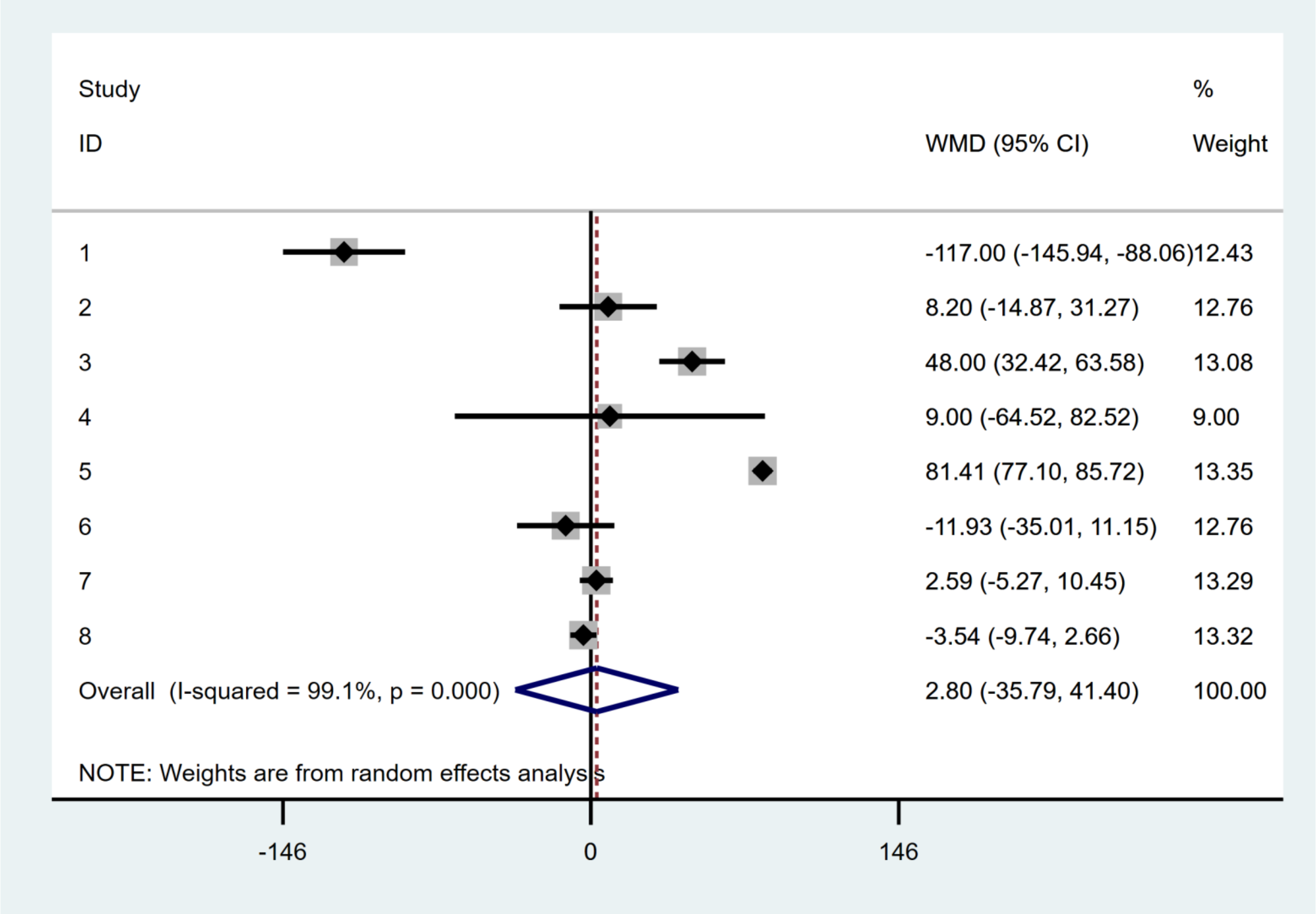

FigureS5(l) Forest plot of mean difference of 6MWT

Subgroup of patients with T2DM and CVD

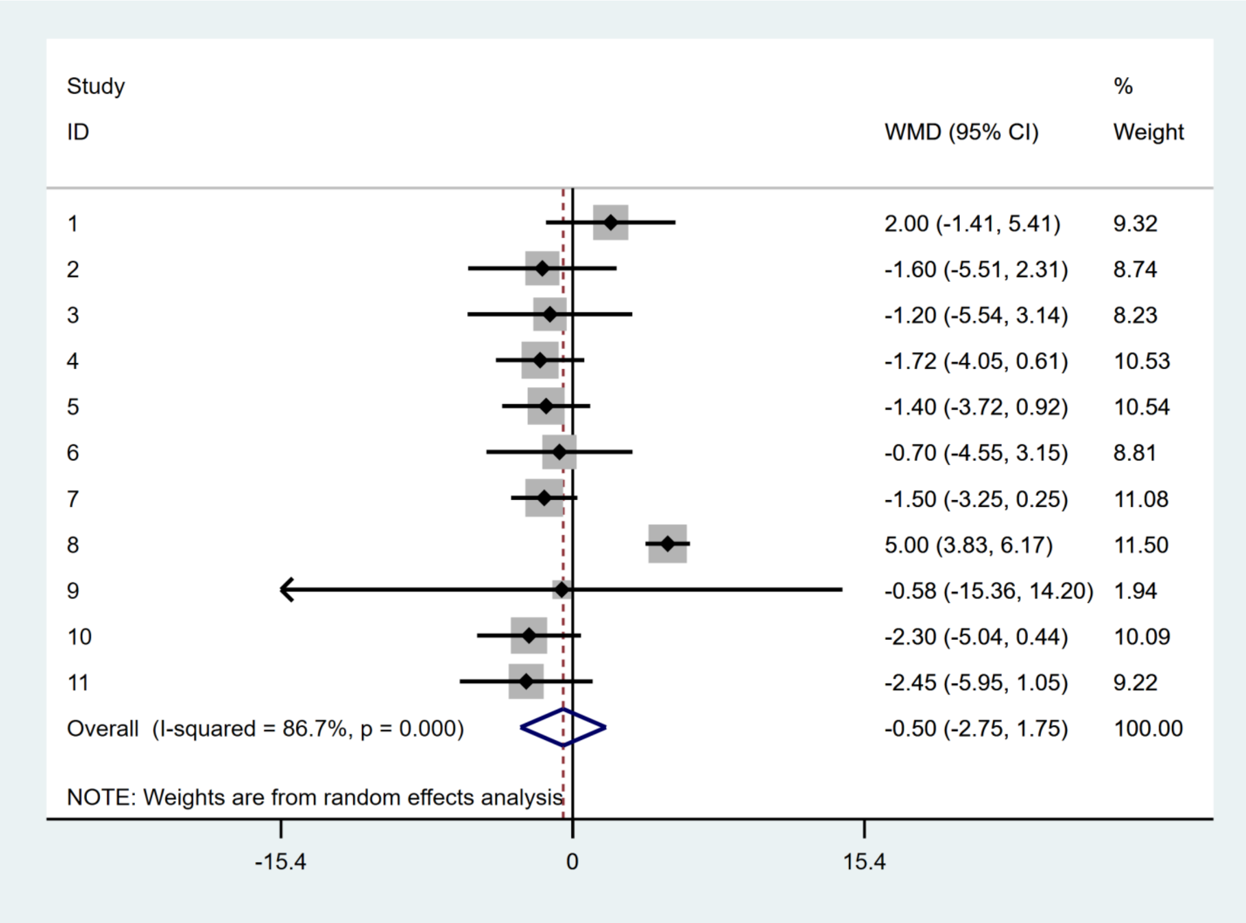

FigureS5(m) Forest plot of mean difference of LVEF%

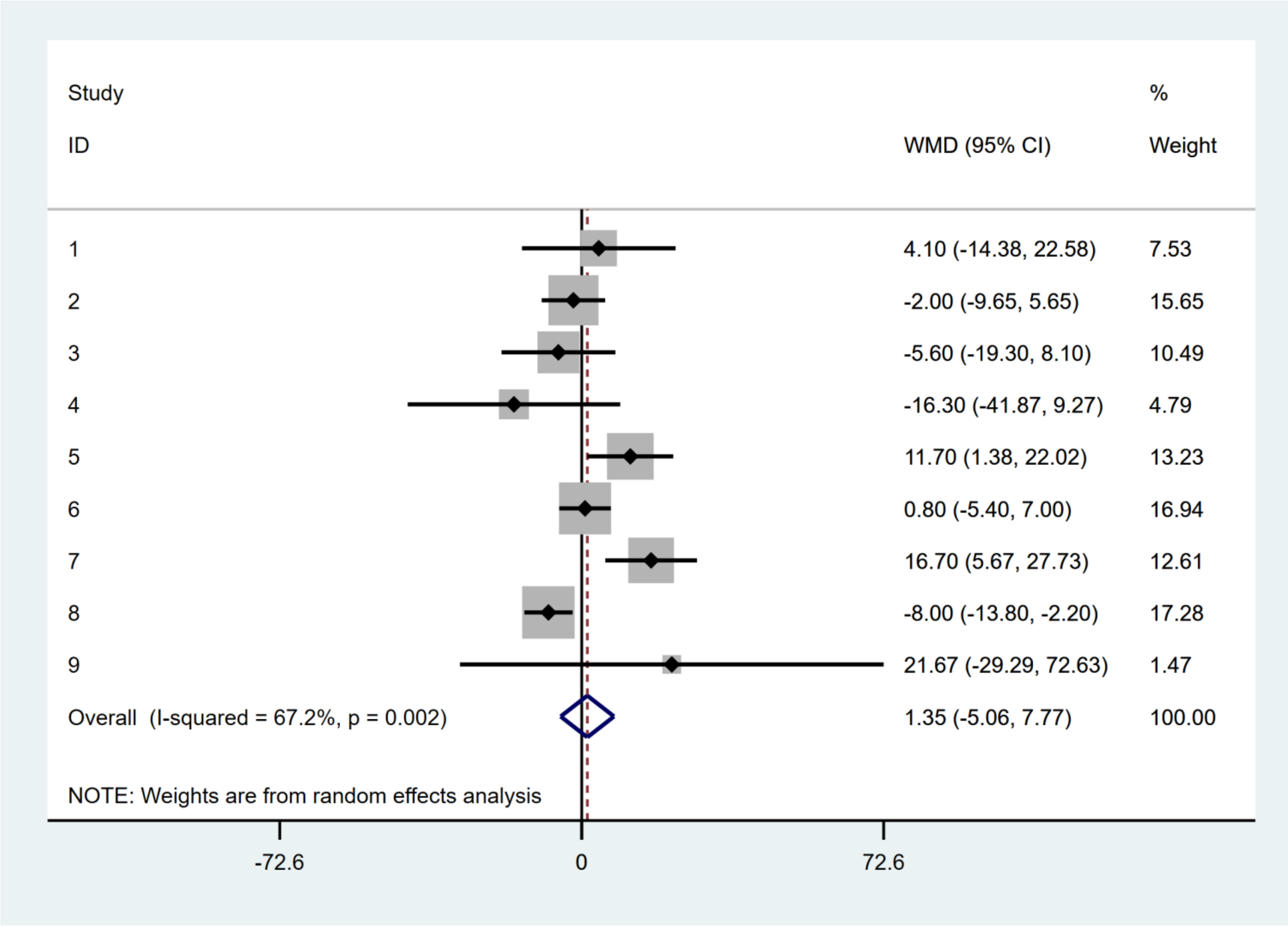

FigureS5(n) Forest plot of mean difference of LVEDV

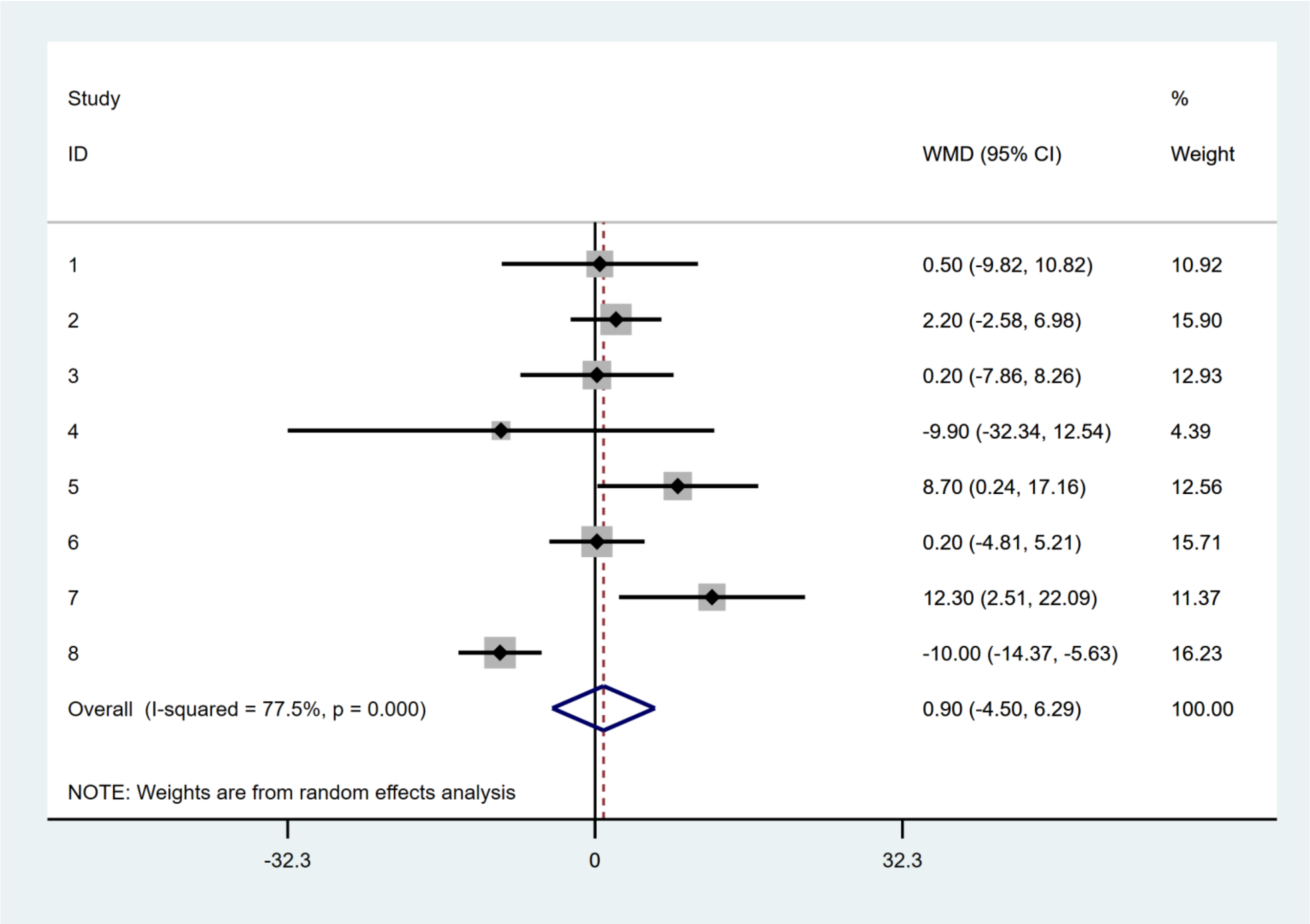

FigureS5(o) Forest plot of mean difference of LVESV

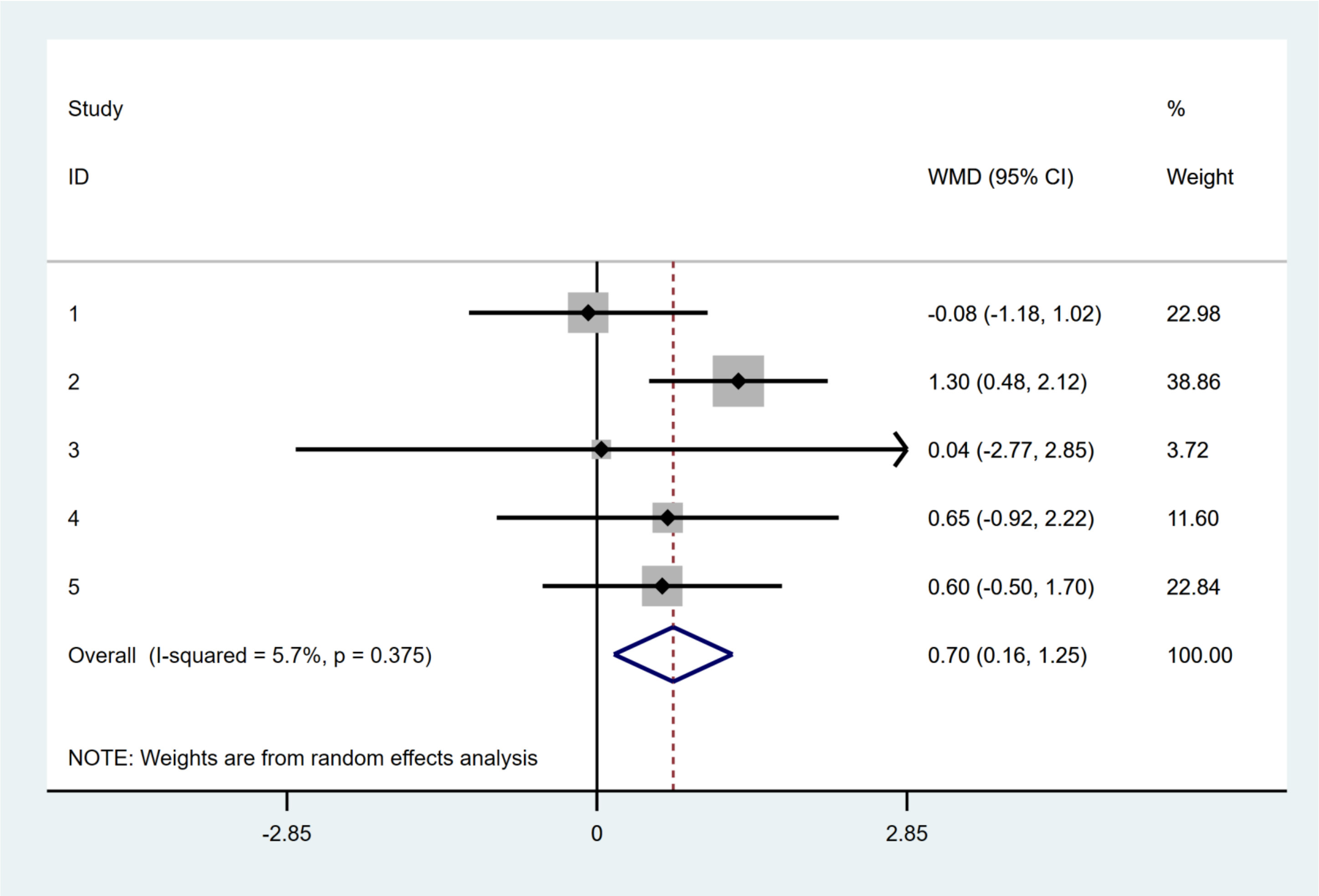

FigureS5(p) Forest plot of mean difference of E/e’

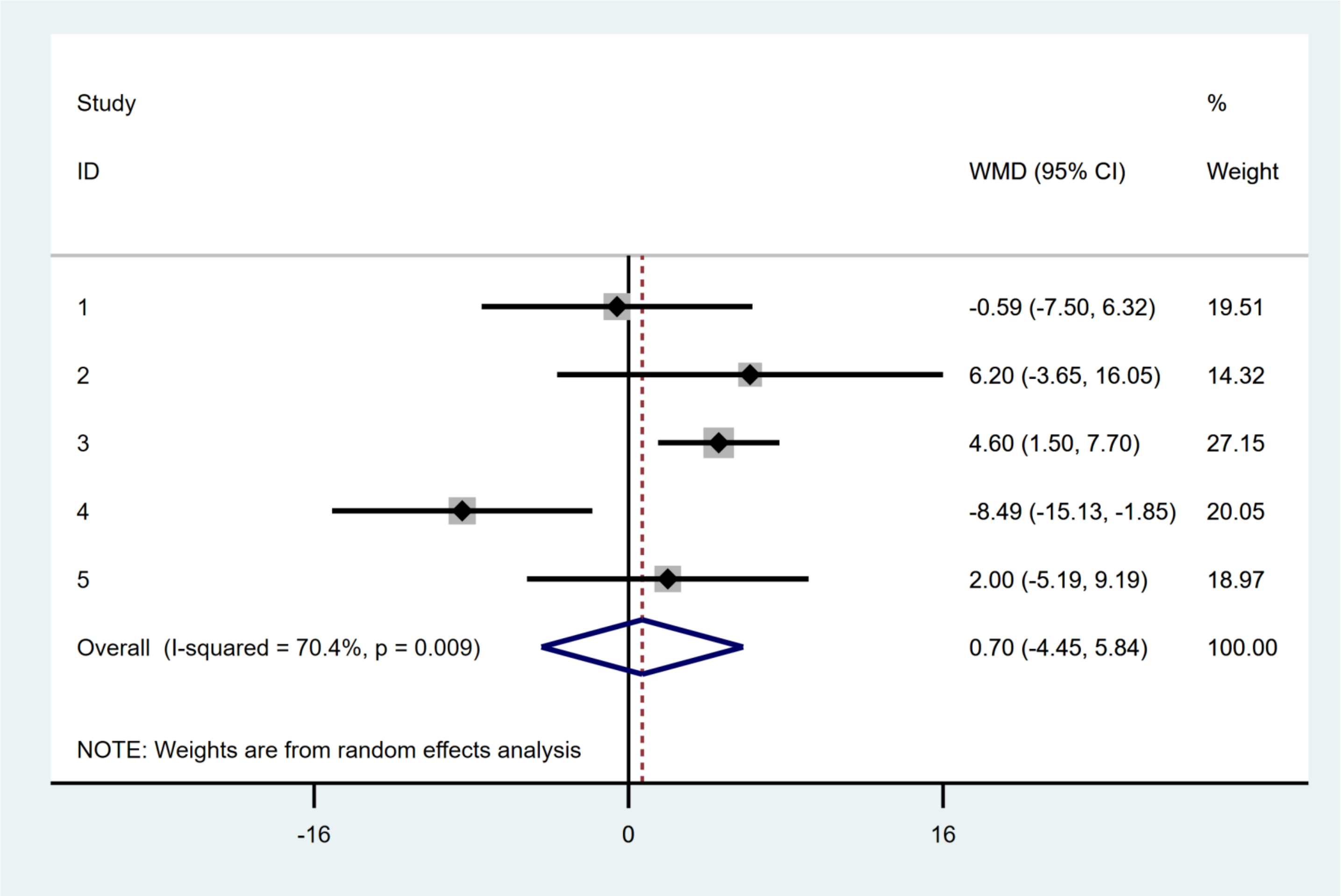

FigureS5(q) Forest plot of mean difference of SBP

Subgroup of patients with CVD alone

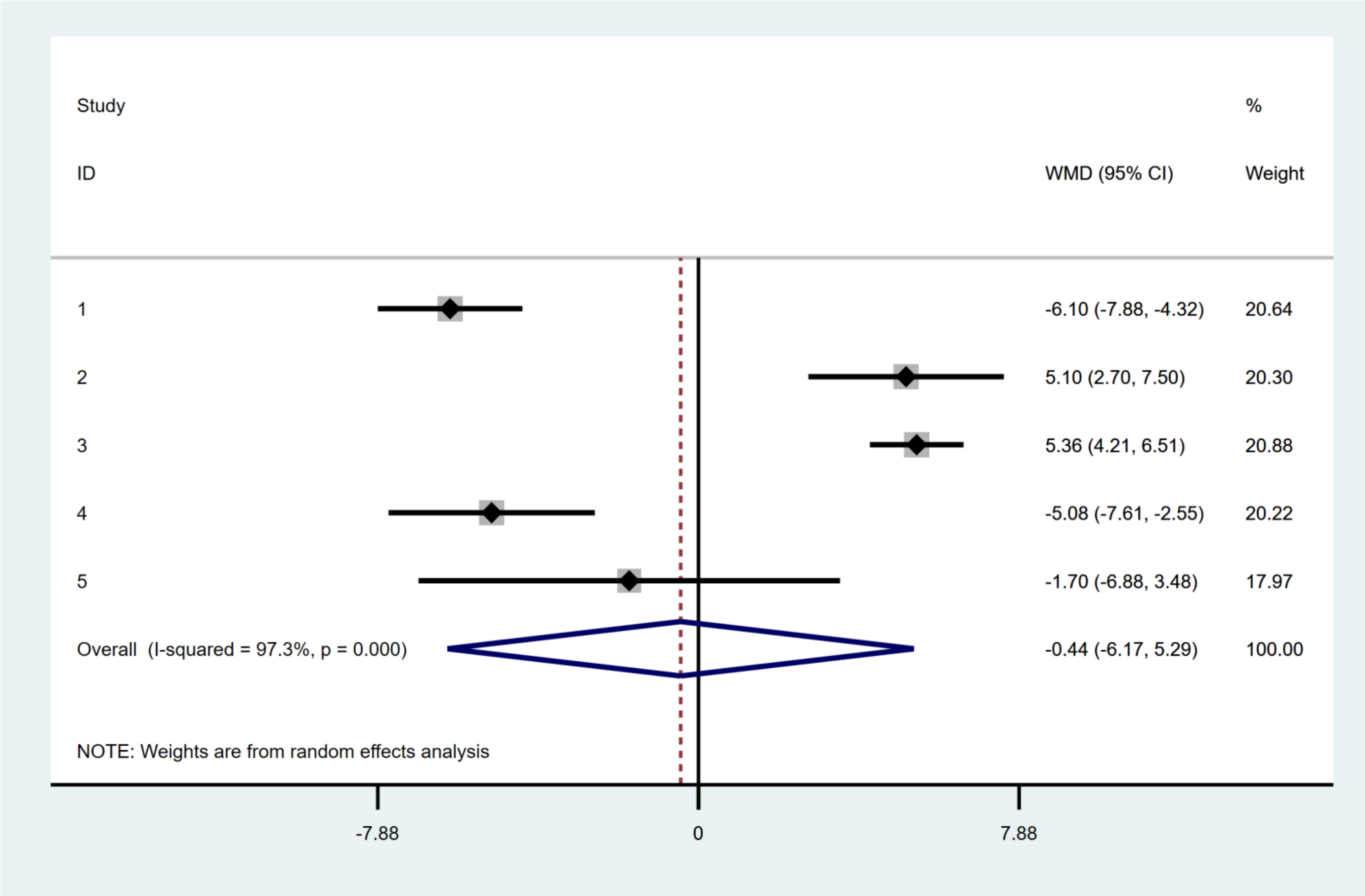

FigureS5(r) Forest plot of mean difference of LVEF%

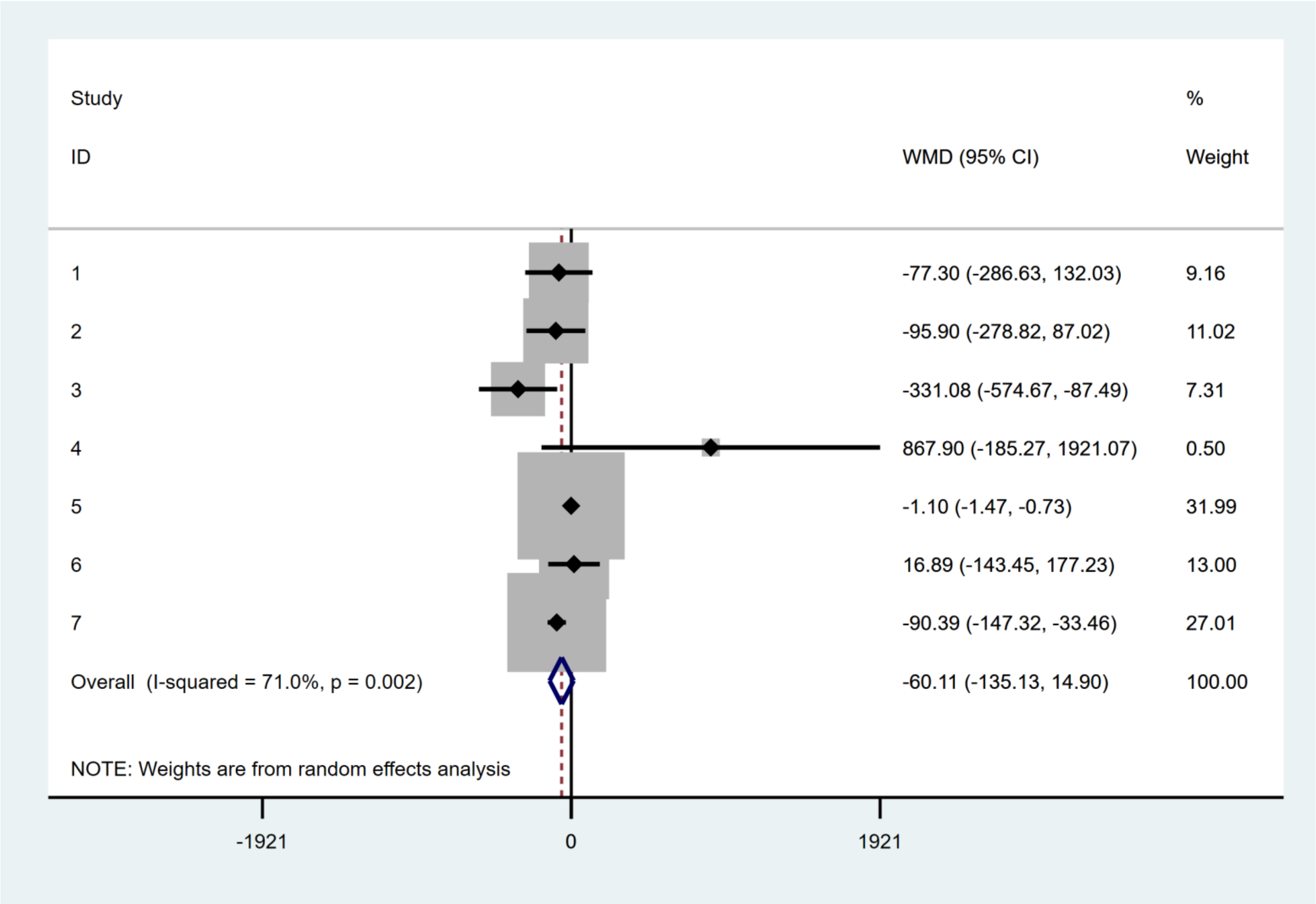

FigureS5(s) Forest plot of mean difference of NT-pro BNP

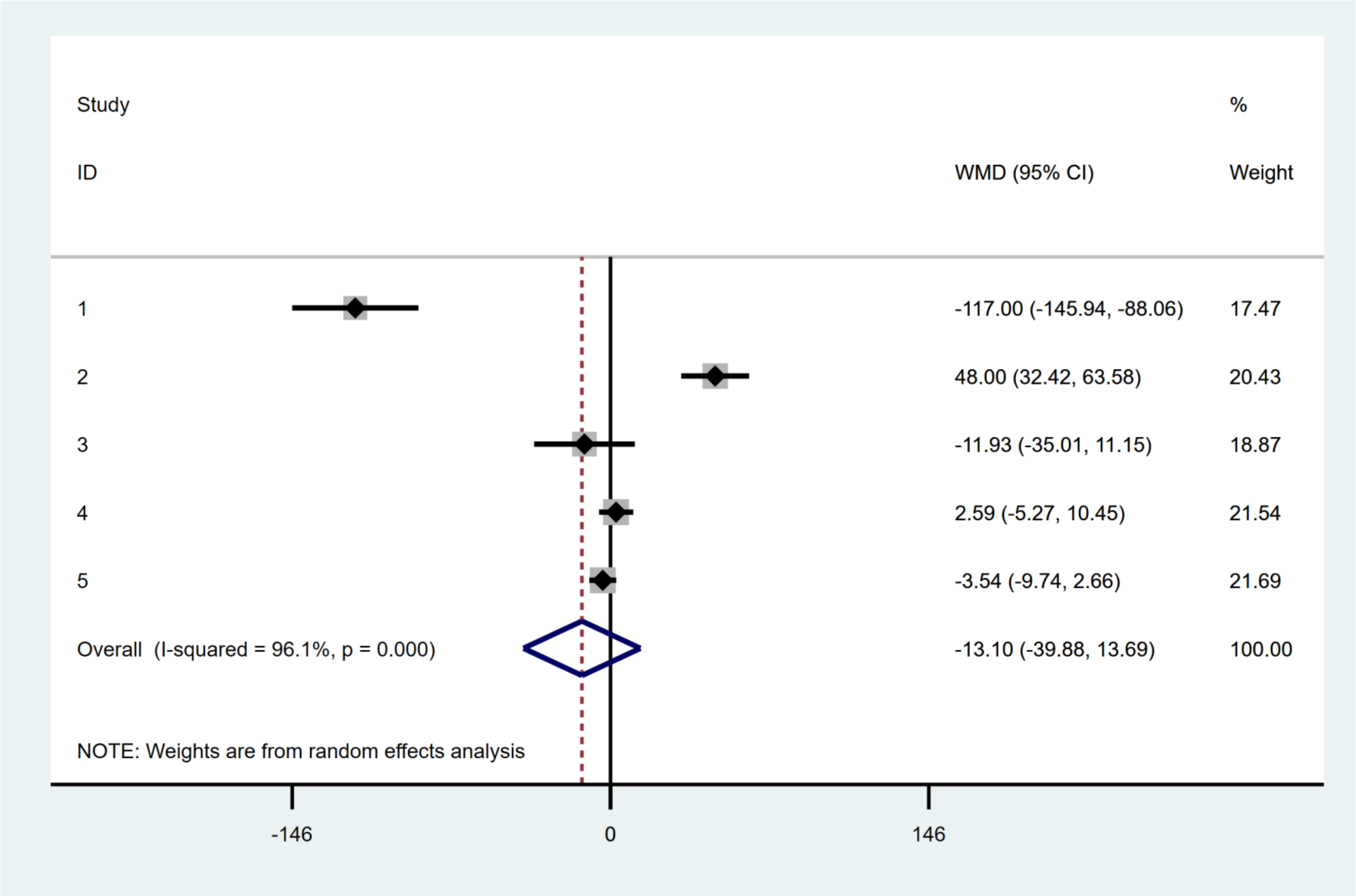

FigureS5(t) Forest plot of mean difference of 6MWT

Note: e': early diastolic velocity; **E/e'**: mitral inflow E velocity to tissue doppler e' ratio; **E/A**: early diastolic to late diastolic velocities ratio**; CVD**: cardiovascular disease; **DPP-4i**: dipeptidyl peptidase-4 inhibitor; **GLP-1RA**: glucagon-like peptide-1 receptor agonist; **LVEDD**: left ventricular end-diastolic diameter; **LVEDV**: LV end-diastolic volume; **LVEF**: LV ejection fraction; **LVESD**: LV end-systolic diameter; **LVESV**: LV end-systolic volume; **LVMI**: LV mass index; **NT-pro BNP**: immunoreactive amino-terminal pro-brain natriuretic peptide; **SBP**: systolic blood pressure; **SGLT-2i**: sodium glucose cotransporter type 2 inhibitor; **T2DM**: type 2 diabetes; **6MWT**: 6-min walk test.
