# Supplementary material for: Effects of new hypoglycemic drugs on cardiac remodeling: a systematic review and network meta-analysis
Source: BMC Cardiovasc Disord. 2023 Jun 9;23:293. doi: 10.1186/s12872-023-03324-6 (PMC10251583; doi:10.1186/s12872-023-03324-6)
Supplement: Supplementary file 7 — Additional file 7: Table S3. Treatment Rankings. Note: e’: early diastolic velocity; E/e’: mitral inflow E velocity to tissue doppler e’ ratio; E/A: early diastolic to late diastolic velocities ratio; CVD: cardiovascular disease; DPP-4i: dipeptidyl peptidase-4 inhibitor; GLP-1RA: glucagon-like peptide-1 receptor agonist; LVEDD: left ventricular end-diastolic diameter; LVEDV: LV end-diastolic volume; LVEF: LV ejection fraction; LVESD: LV end-systolic diameter; LVESV: LV end-systolic volume; LVMI: LV mass index; NT-pro BNP: immunoreactive amino-terminal pro-brain natriuretic peptide; SBP: systolic blood pressure; SGLT-2i: sodium glucose cotransporter type 2 inhibitor; T2DM: type 2 diabetes; 6MWT: 6-min walk test. [file 12872_2023_3324_MOESM7_ESM.pdf]

Table S3 Treatment Rankings

Treatment	LVEF	LVEDD	LVESD	LVEDV	LVESV	LVMI	e'	E/e'	E/A	SBP	NT-pro BNP	6MWT
Placebo	1	4	3	2	3	4	3	2	3	2	2	3
GLP-1RA	2	2	1	1	1	2	4	1	2	4	1	1
DPP-4i	4	3	-	3	-	1	1	3	1	1	4	-
SGLT-2i	3	1	2	4	2	3	2	4	4	3	3	2

Note: e': early diastolic velocity; **E/e'**: mitral inflow E velocity to tissue doppler e' ratio; **E/A**: early diastolic to late diastolic velocities ratio**; CVD**: cardiovascular disease; **DPP-4i**: dipeptidyl peptidase-4 inhibitor; **GLP-1RA**: glucagon-like peptide-1 receptor agonist; **LVEDD**: left ventricular end-diastolic diameter; **LVEDV**: LV end-diastolic volume; **LVEF**: LV ejection fraction; **LVESD**: LV end-systolic diameter; **LVESV**: LV end-systolic volume; **LVMI**: LV mass index; **NT-pro BNP**: immunoreactive amino-terminal pro-brain natriuretic peptide; **SBP**: systolic blood pressure; **SGLT-2i**: sodium glucose cotransporter type 2 inhibitor; **T2DM**: type 2 diabetes; **6MWT**: 6-min walk test.
