# Supplementary material for: Effects of new hypoglycemic drugs on cardiac remodeling: a systematic review and network meta-analysis
Source: BMC Cardiovasc Disord. 2023 Jun 9;23:293. doi: 10.1186/s12872-023-03324-6 (PMC10251583; doi:10.1186/s12872-023-03324-6)
Supplement: Supplementary file 8 — Additional file 8: Figure S6. Curve diagram of SUCRA of outcome indicators.Curve diagram of SUCRA of LVEF% Note: 1 DDP-4i, 2 GLP-1RA, 3 Placebo, 4 SGLT-2i.Curve diagram of SUCRA of LVEDD Note: 1 DDP-4i, 2 GLP-1RA, 3 Placebo, 4 SGLT-2i.Curve diagram of SUCRA of LVEDV Note: 1 DDP-4i, 2 GLP-1RA, 3 Placebo, 4 SGLT-2i.Curve diagram of SUCRA of LVESD Note: 1 GLP-1RA, 2 Placebo, 3 SGLT-2i.Curve diagram of SUCRA of LVESV Note: 1 GLP-1RA, 2 Placebo, 3 SGLT-2i.Curve diagram of SUCRA of LVMI Note: 1 DDP-4i, 2 GLP-1RA, 3 Placebo, 4 SGLT-2i.Curve diagram of SUCRA of e’ Note: 1 DDP-4i, 2 GLP-1RA, 3 Placebo, 4 SGLT-2i.Curve diagram of SUCRA of E/e’ Note: 1 DDP-4i, 2 GLP-1RA, 3 Placebo, 4 SGLT-2i.Curve diagram of SUCRA of E/A Note: 1 DDP-4i, 2 GLP-1RA, 3 Placebo, 4 SGLT-2i.Curve diagram of SUCRA of SBP Note: 1 DDP-4i, 2 GLP-1RA, 3 Placebo, 4 SGLT-2i.Curve diagram of SUCRA of NT-pro BNP Note: 1 DDP-4i, 2 GLP-1RA, 3 Placebo, 4 SGLT-2i.Curve diagram of SUCRA of 6MWT Note: 1 GLP-1RA, 2 Placebo, 3 SGLT-2i.Curve diagram of SUCRA of LVEF%. Note: 1 DDP-4i, 2 GLP-1RA, 3 Placebo, 4 SGLT-2i.Curve diagram of SUCRA of LVEDV Note: 1 DDP-4i, 2 GLP-1RA, 3 Placebo, 4 SGLT-2i.Curve diagram of SUCRA of LVESV Note: 1 DDP-4i, 2 GLP-1RA, 3 Placebo, 4 SGLT-2i.Curve diagram of SUCRA of E/e’ Note: 1 DDP-4i, 2 GLP-1RA, 3 Placebo, 4 SGLT-2i.Curve diagram of SUCRA of SBP Note: 1 DDP-4i, 2 GLP-1RA, 3 Placebo, 4 SGLT-2i.Curve diagram of SUCRA of LVEF%. Note: 1 GLP-1RA, 2 Placebo, 3 SGLT-2i.Curve diagram of SUCRA of NT-pro BNP. Note: 1 GLP-1RA, 2 Placebo, 3 SGLT-2i.Curve diagram of SUCRA of 6MWT. Note: 1 GLP-1RA, 2 Placebo, 3 SGLT-2i Note: e’: early diastolic velocity; E/e’: mitral inflow E velocity to tissue doppler e’ ratio; E/A: early diastolic to late diastolic velocities ratio; CVD: cardiovascular disease; DPP-4i: dipeptidyl peptidase-4 inhibitor; GLP-1RA: glucagon-like peptide-1 receptor agonist; LVEDD: left ventricular end-diastolic diameter; LVEDV: LV end-diastolic volume; LVEF: LV ejection fraction; LVE [file 12872_2023_3324_MOESM8_ESM.pdf]

Figure S6 Curve diagram of SUCRA of outcome indicators

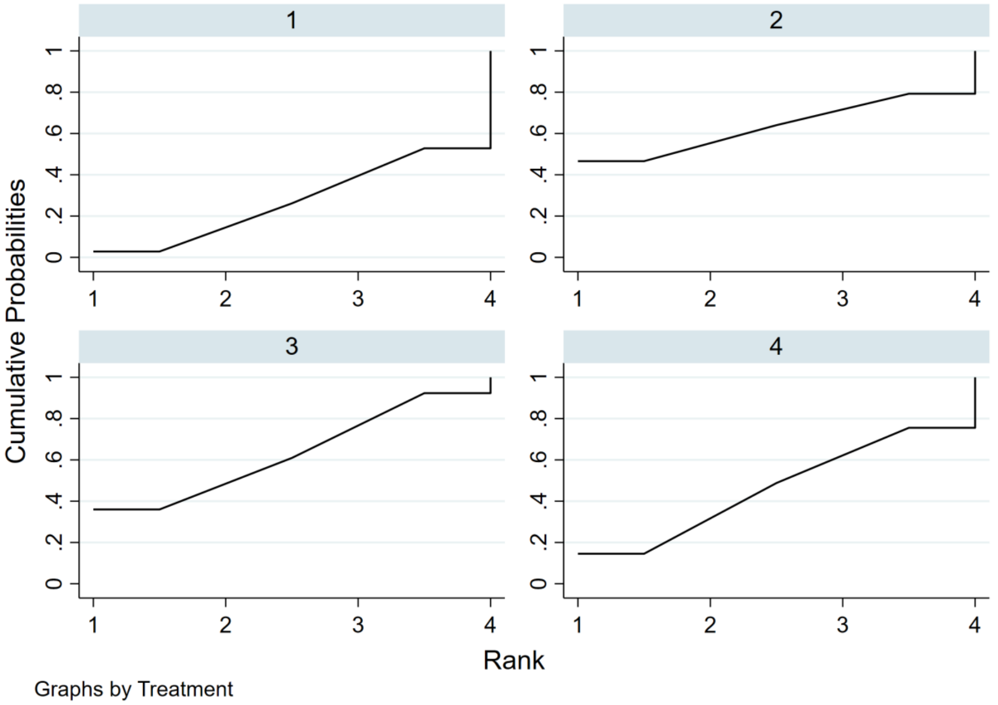

Figure S6(a) Curve diagram of SUCRA of LVEF%

Note: 1 DDP-4i, 2 GLP-1RA, 3 Placebo, 4 SGLT-2i

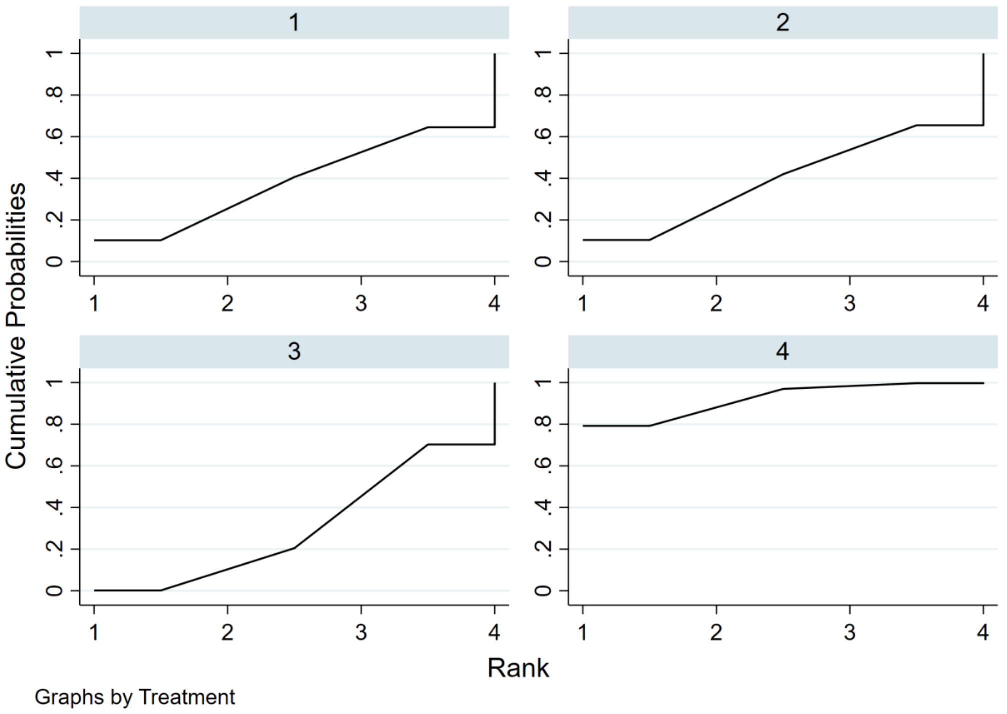

Figure S6(b) Curve diagram of SUCRA of LVEDD

Note: 1 DDP-4i, 2 GLP-1RA, 3 Placebo, 4 SGLT-2i

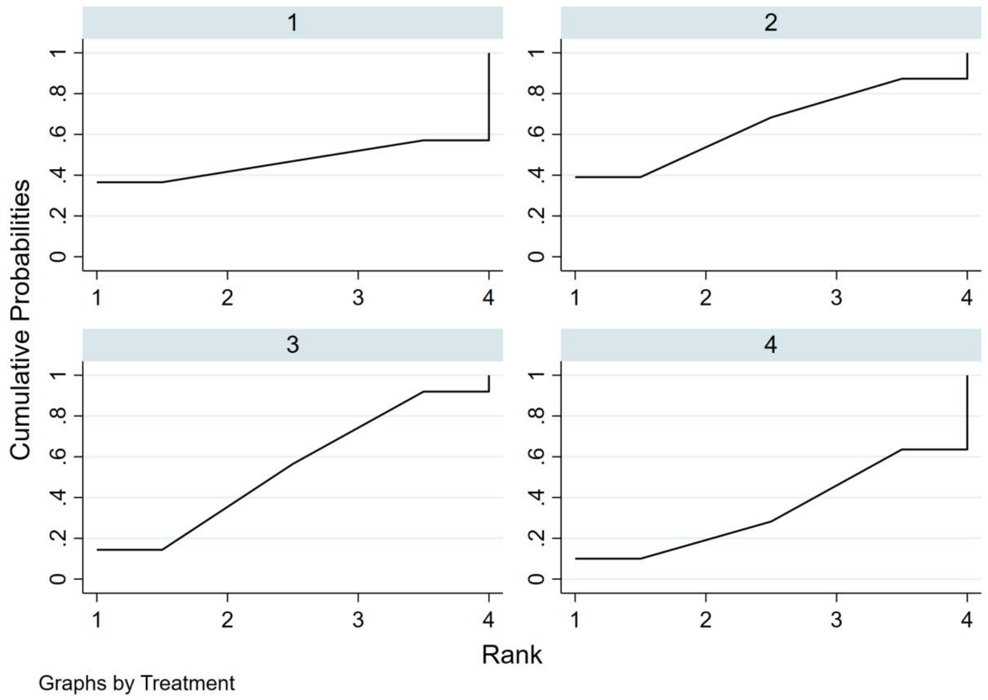

Figure S6(c) Curve diagram of SUCRA of LVEDV

Note: 1 DDP-4i, 2 GLP-1RA, 3 Placebo, 4 SGLT-2i

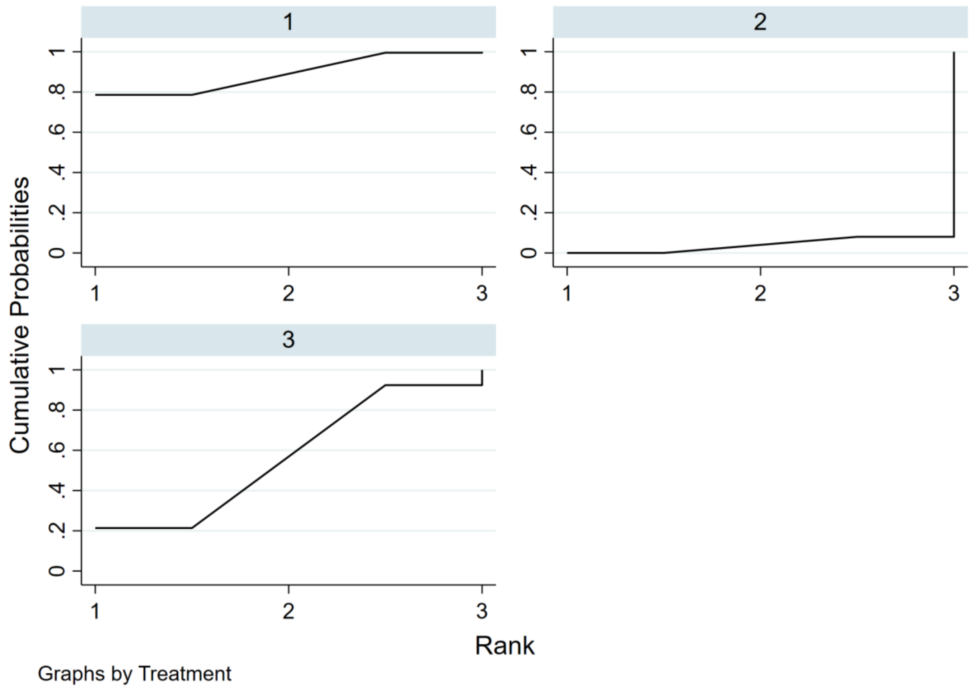

Figure S6(d) Curve diagram of SUCRA of LVESD

Note: 1 GLP-1RA, 2 Placebo, 3 SGLT-2i

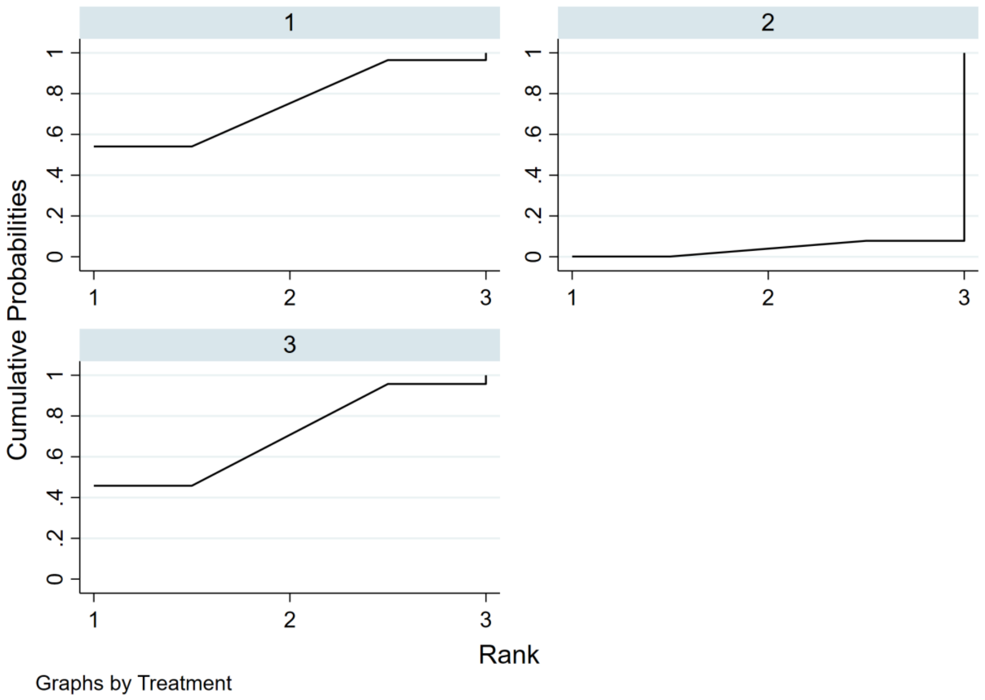

Figure S6(e) Curve diagram of SUCRA of LVESV

Note: 1 GLP-1RA, 2 Placebo, 3 SGLT-2i

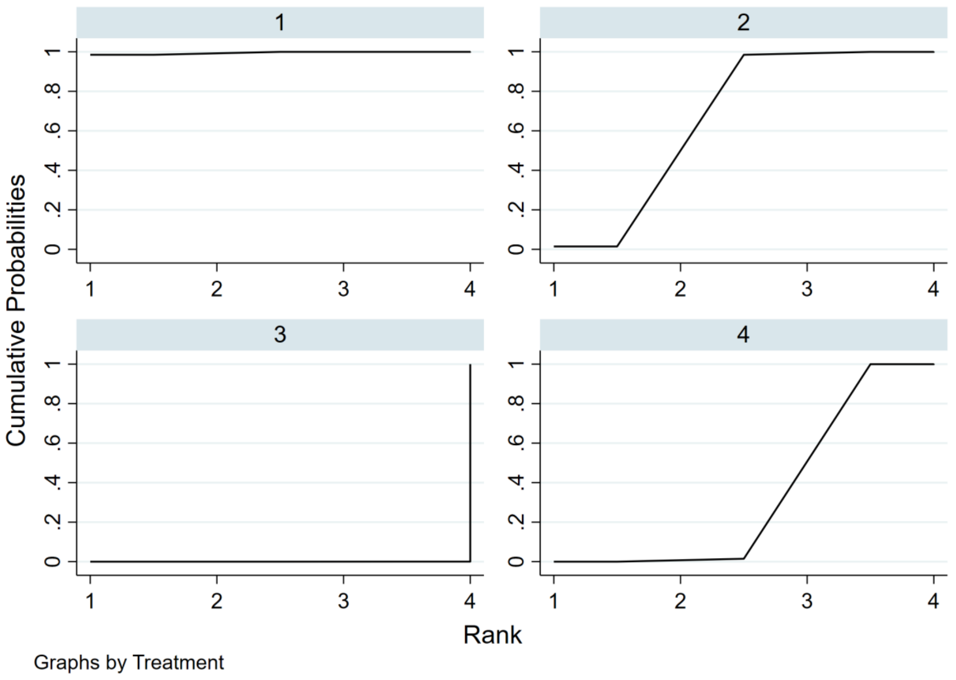

Figure S6(f) Curve diagram of SUCRA of LVMI

Note: 1 DDP-4i, 2 GLP-1RA, 3 Placebo, 4 SGLT-2i

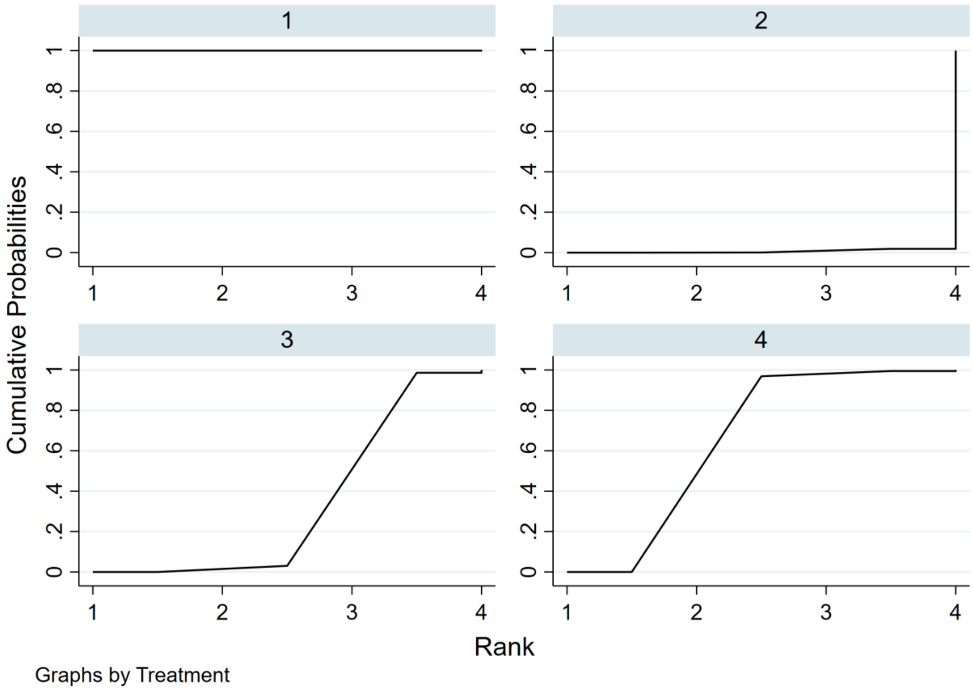

Figure S6(g) Curve diagram of SUCRA of e’

Note: 1 DDP-4i, 2 GLP-1RA, 3 Placebo, 4 SGLT-2i

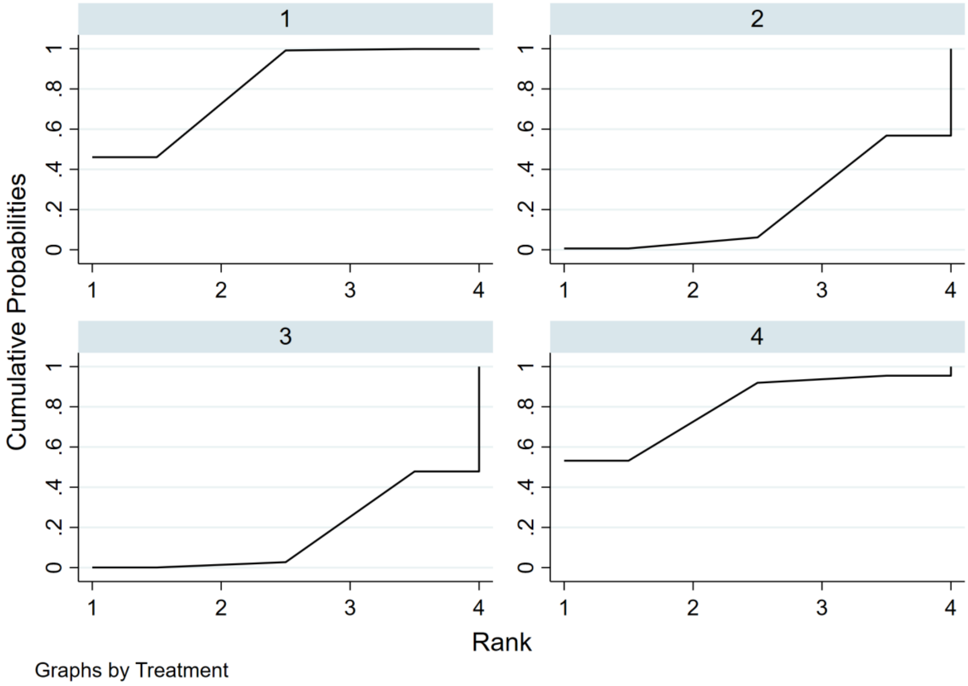

Figure S6(h) Curve diagram of SUCRA of E/e’

Note: 1 DDP-4i, 2 GLP-1RA, 3 Placebo, 4 SGLT-2i

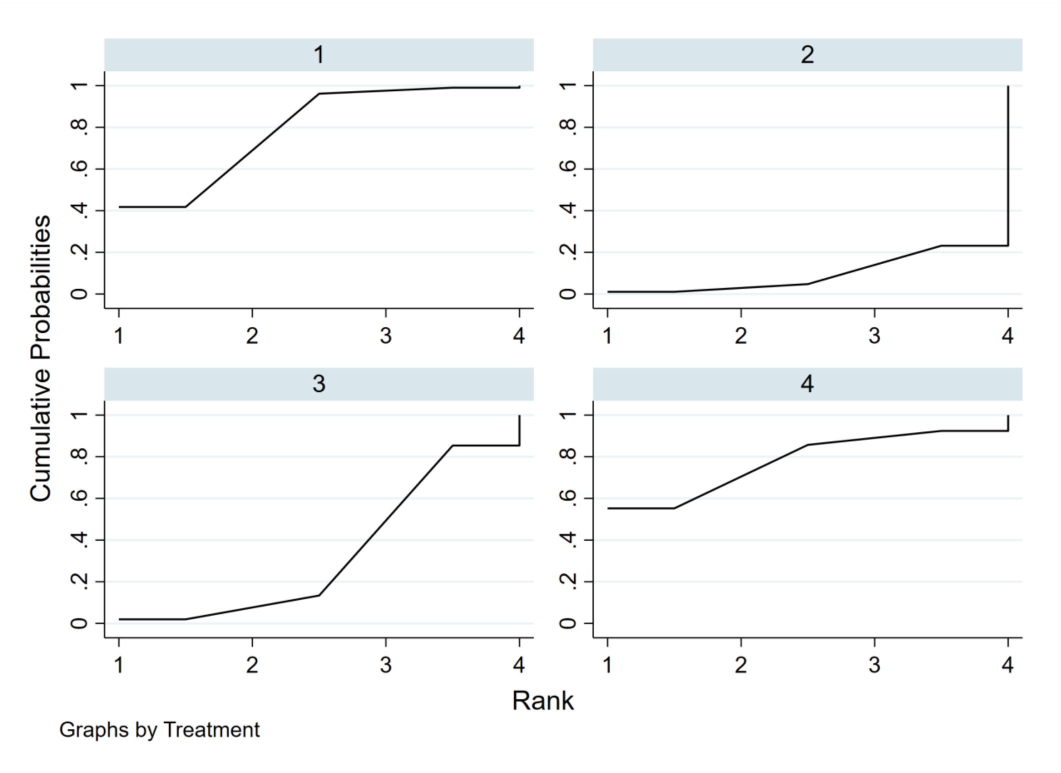

Figure S6(i) Curve diagram of SUCRA of E/A

Note: 1 DDP-4i, 2 GLP-1RA, 3 Placebo, 4 SGLT-2i

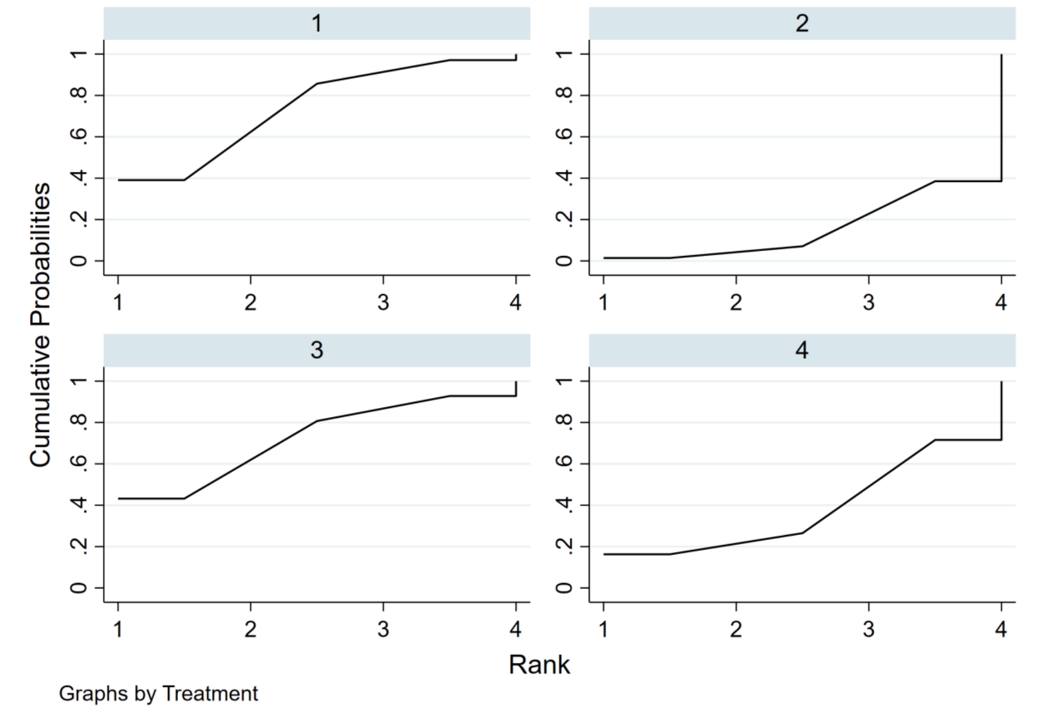

Figure S6(j) Curve diagram of SUCRA of SBP

Note: 1 DDP-4i, 2 GLP-1RA, 3 Placebo, 4 SGLT-2i

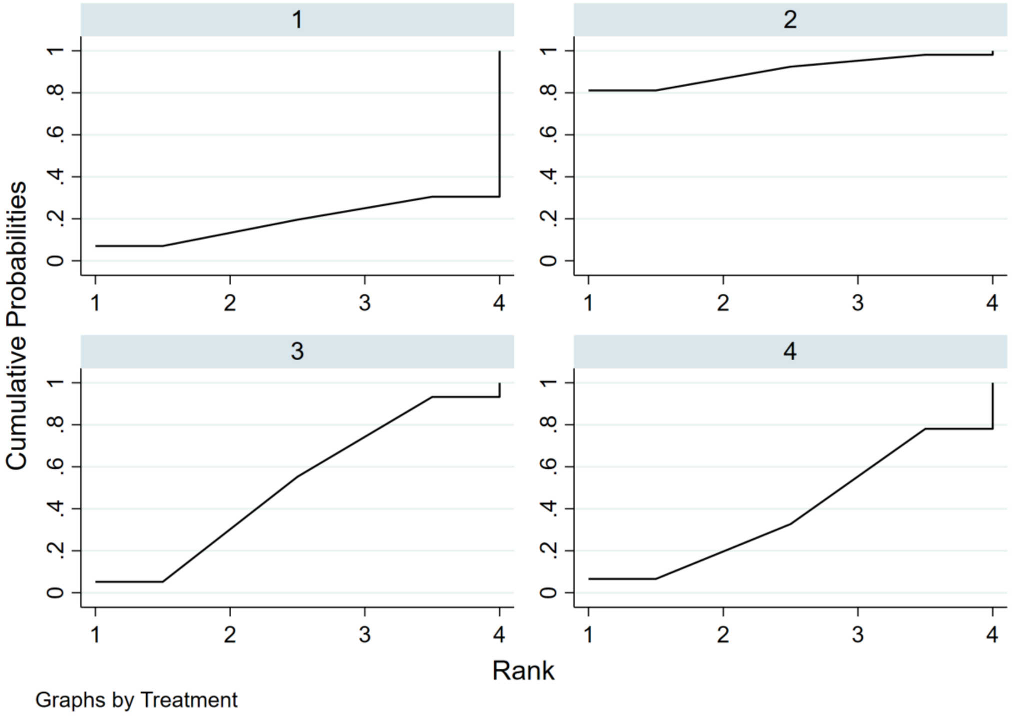

Figure S6(k) Curve diagram of SUCRA of NT-pro BNP

Note: 1 DDP-4i, 2 GLP-1RA, 3 Placebo, 4 SGLT-2i

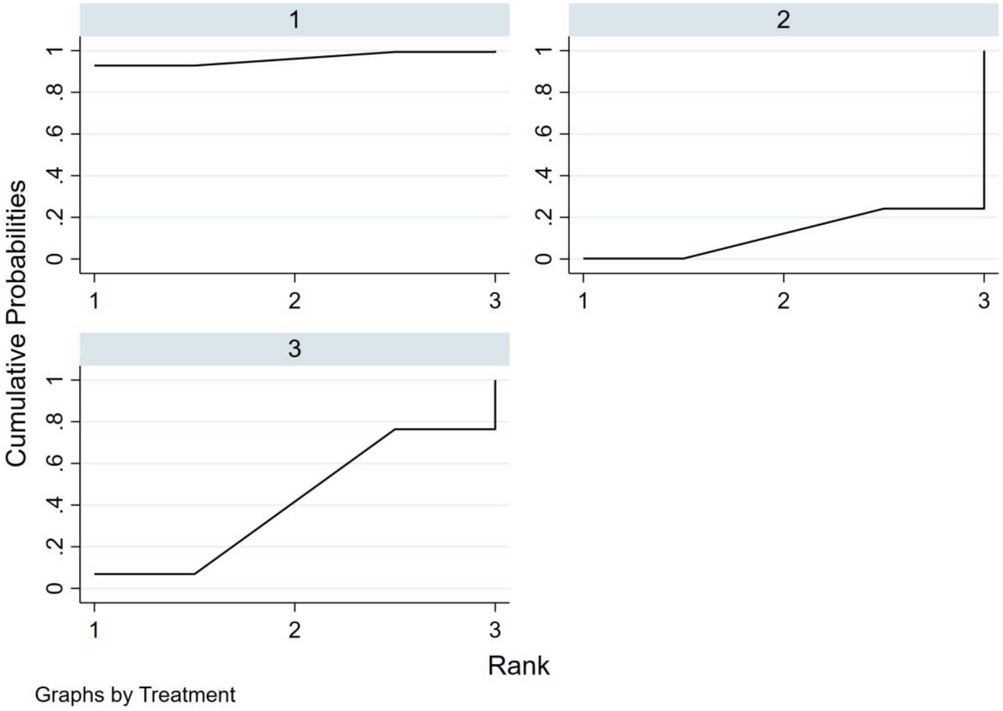

Figure S6(l) Curve diagram of SUCRA of 6MWT

Note: 1 GLP-1RA, 2 Placebo, 3 SGLT-2i

Subgroup of patients with T2DM and CVD

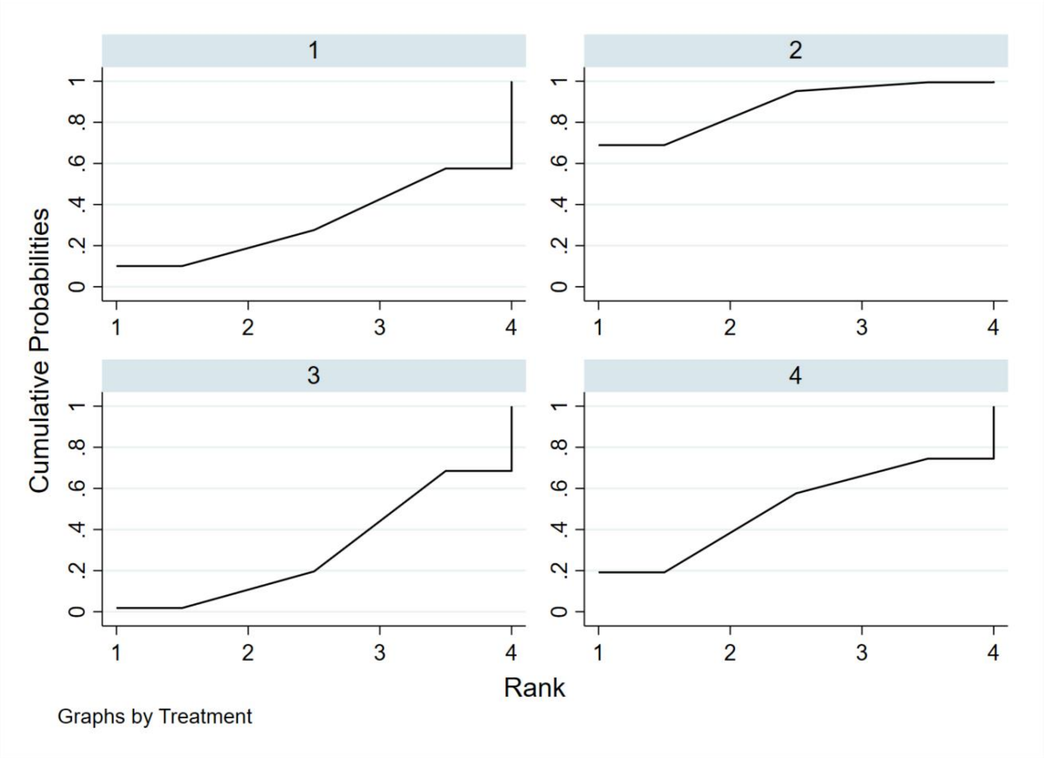

Figure S6(m) Curve diagram of SUCRA of LVEF%.

Note: 1 DDP-4i, 2 GLP-1RA, 3 Placebo, 4 SGLT-2i

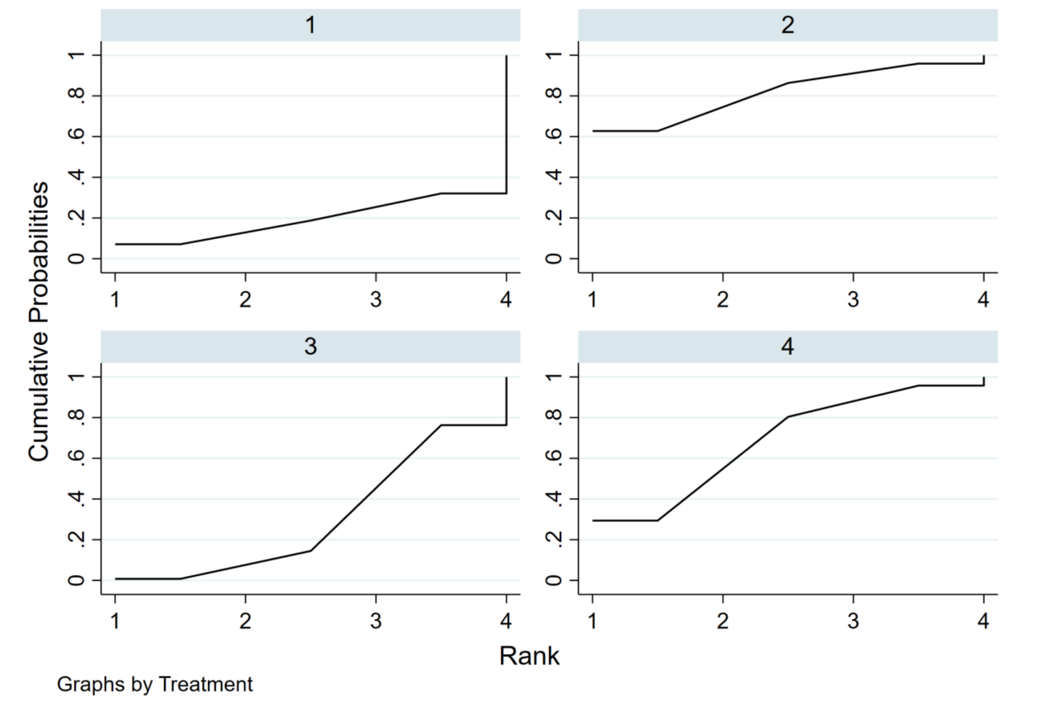

Figure S6(n) Curve diagram of SUCRA of LVEDV

Note: 1 DDP-4i, 2 GLP-1RA, 3 Placebo, 4 SGLT-2i

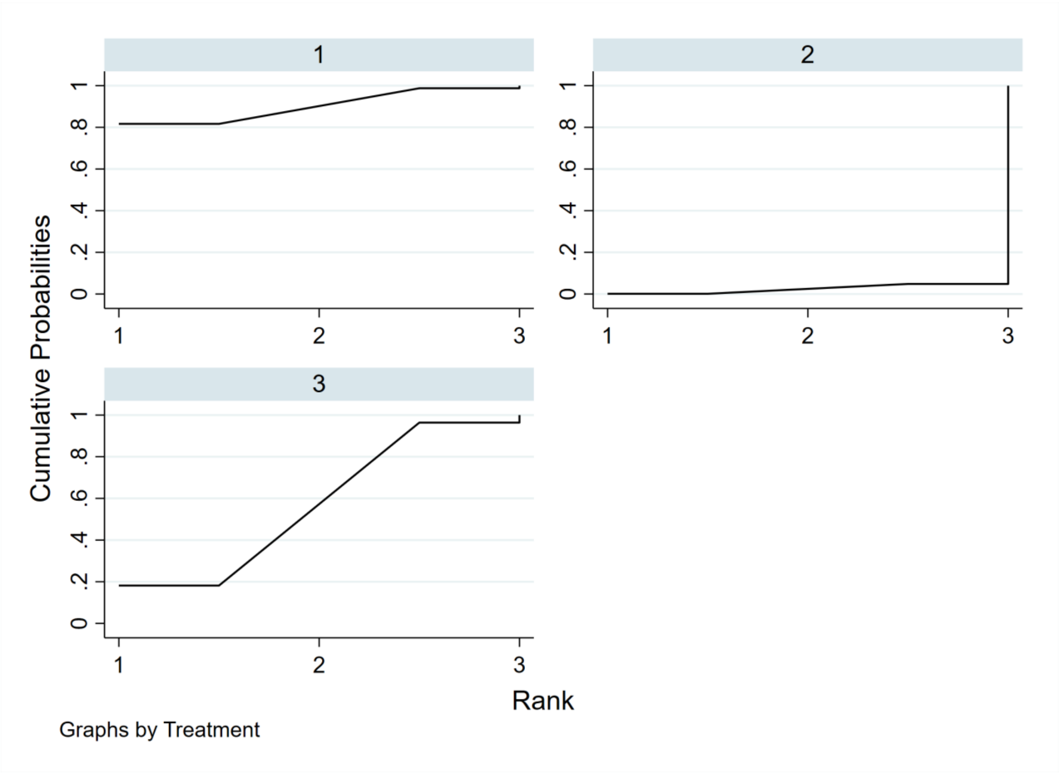

Figure S6(o) Curve diagram of SUCRA of LVESV

Note: 1 DDP-4i, 2 GLP-1RA, 3 Placebo, 4 SGLT-2i

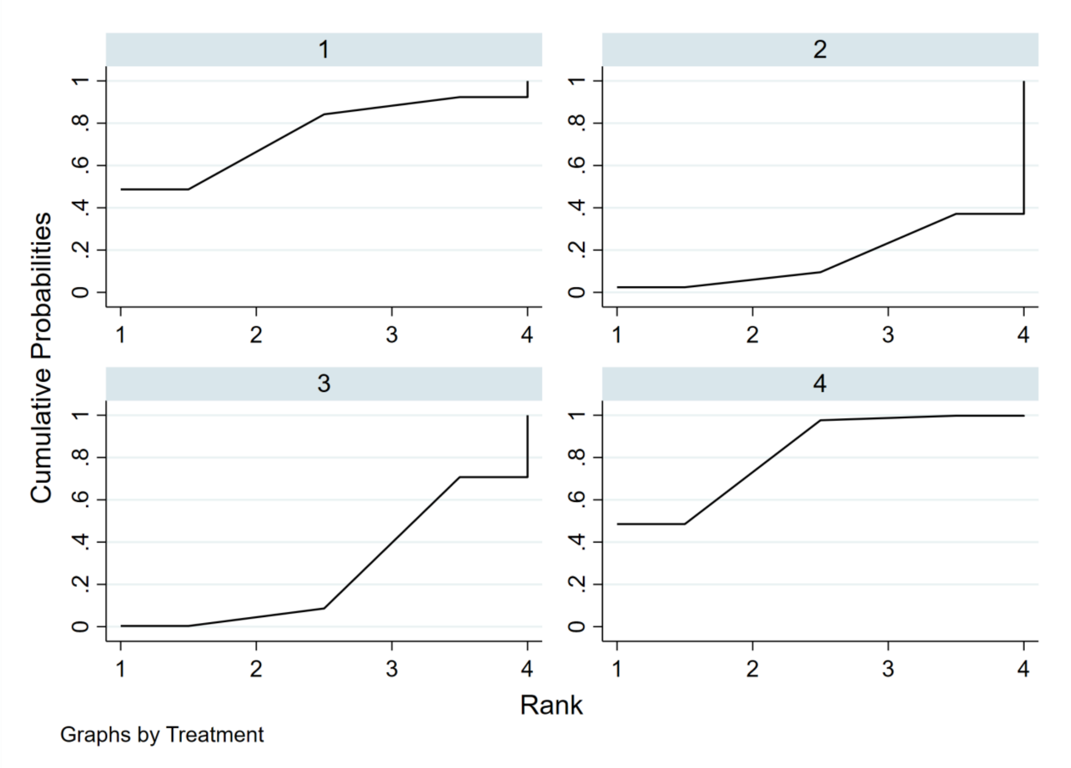

Figure S6(p) Curve diagram of SUCRA of E/e’

Note: 1 DDP-4i, 2 GLP-1RA, 3 Placebo, 4 SGLT-2i

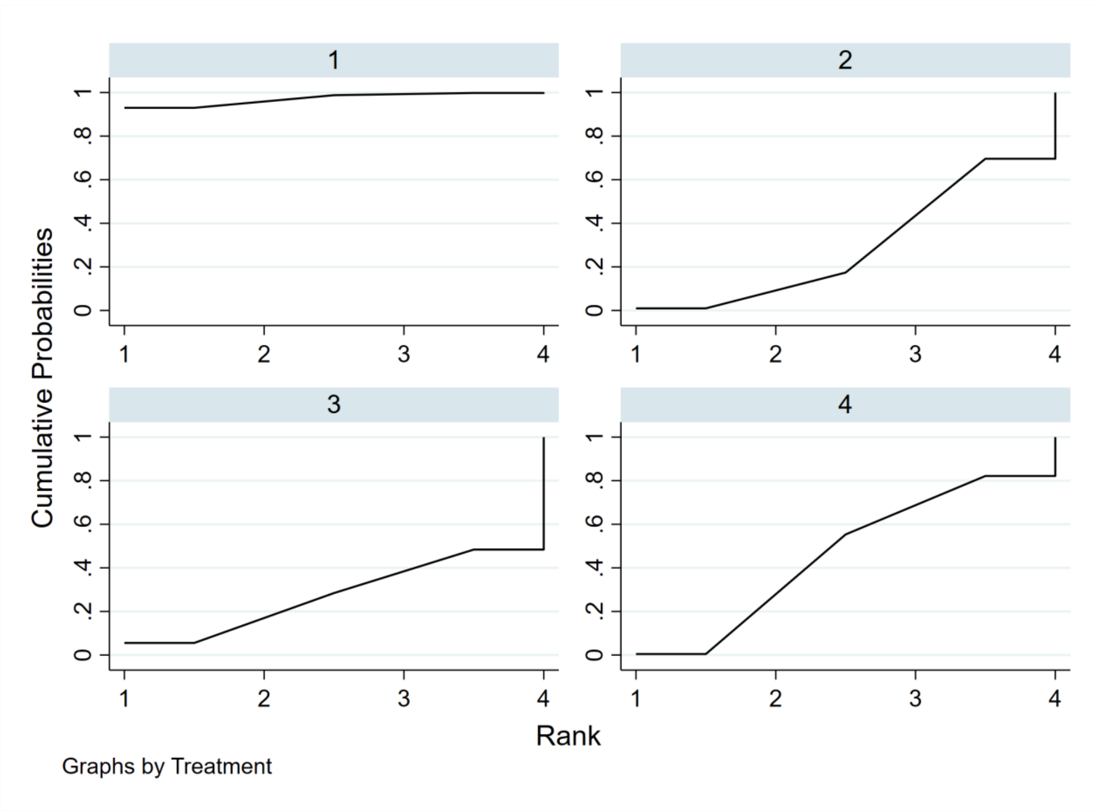

Figure S6(q) Curve diagram of SUCRA of SBP

Note: 1 DDP-4i, 2 GLP-1RA, 3 Placebo, 4 SGLT-2i

Subgroup of patients with CVD alone

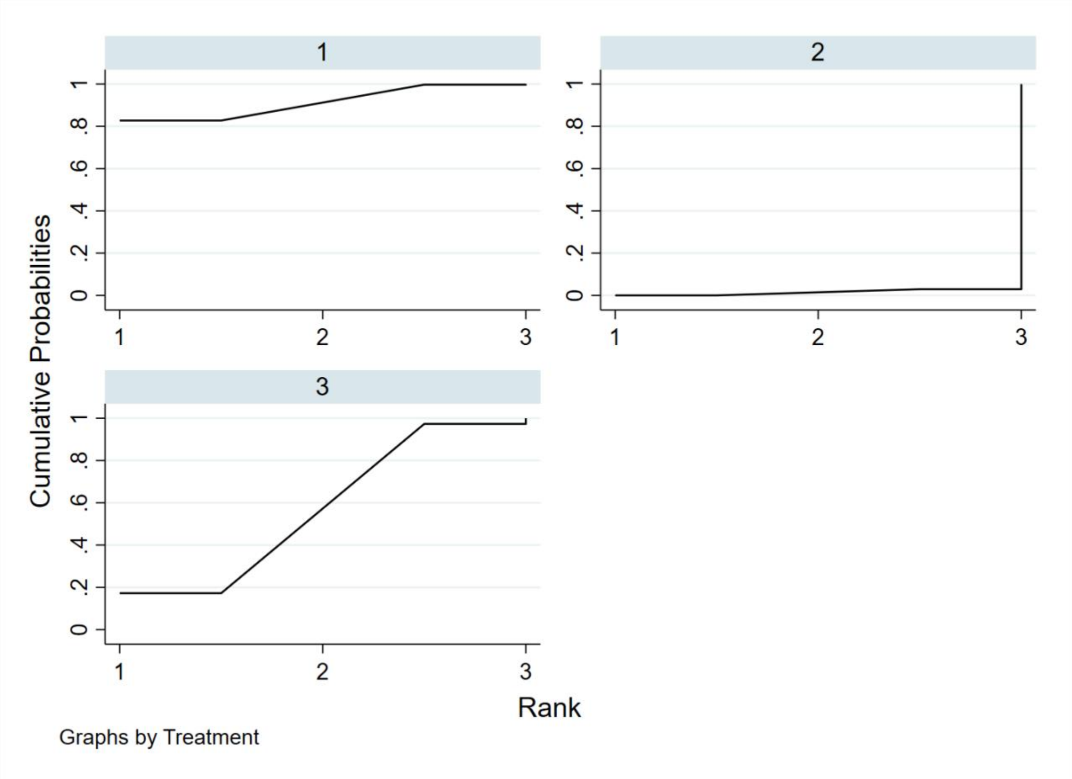

Figure S6(r) Curve diagram of SUCRA of LVEF%.

Note: 1 GLP-1RA, 2 Placebo, 3 SGLT-2i

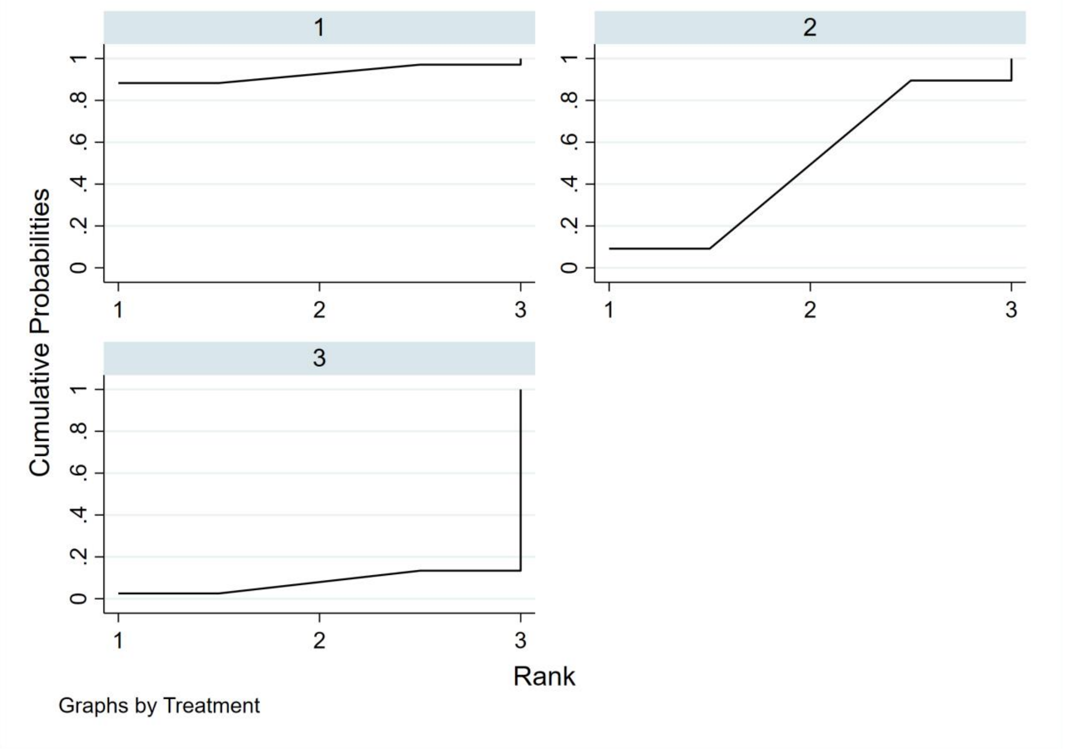

Figure S6(s) Curve diagram of SUCRA of NT-pro BNP.

Note: 1 GLP-1RA, 2 Placebo, 3 SGLT-2i

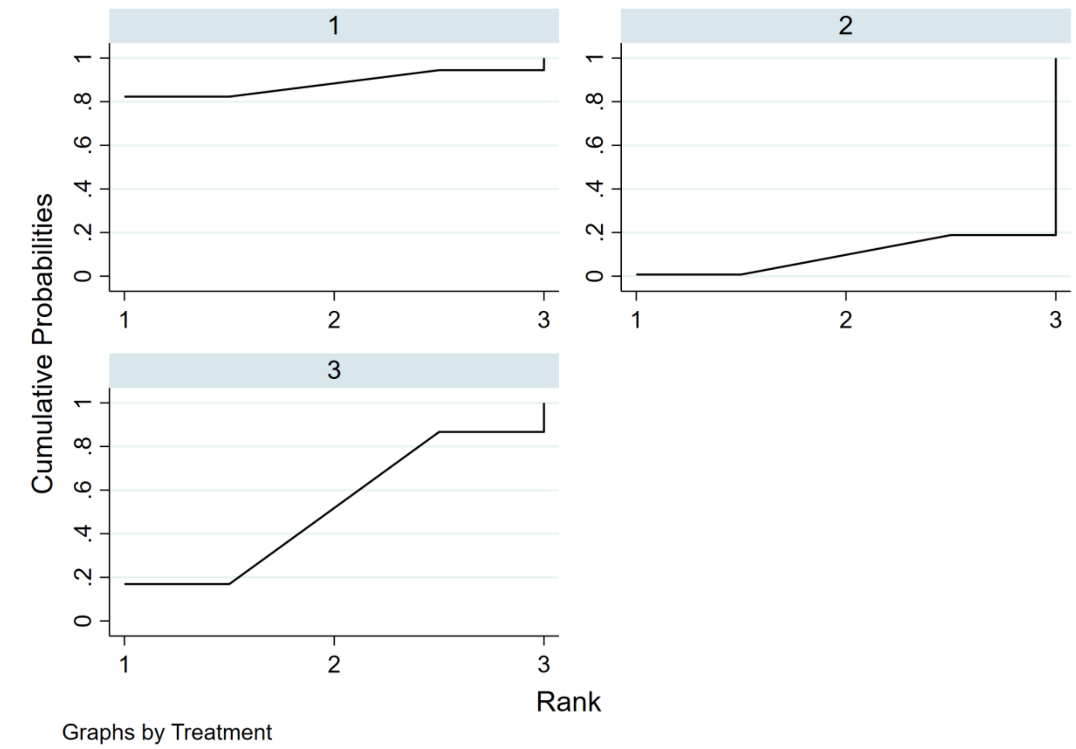

Figure S6(t) Curve diagram of SUCRA of 6MWT.

Note: 1 GLP-1RA, 2 Placebo, 3 SGLT-2i

Note: e': early diastolic velocity; **E/e'**: mitral inflow E velocity to tissue doppler e' ratio; **E/A**: early diastolic to late diastolic velocities ratio**; CVD**: cardiovascular disease; **DPP-4i**: dipeptidyl peptidase-4 inhibitor; **GLP-1RA**: glucagon-like peptide-1 receptor agonist; **LVEDD**: left ventricular end-diastolic diameter; **LVEDV**: LV end-diastolic volume; **LVEF**: LV ejection fraction; **LVESD**: LV end-systolic diameter; **LVESV**: LV end-systolic volume; **LVMI**: LV mass index; **NT-pro BNP**: immunoreactive amino-terminal pro-brain natriuretic peptide; **SBP**: systolic blood pressure; **SGLT-2i**: sodium glucose cotransporter type 2 inhibitor; **T2DM**: type 2 diabetes; **6MWT**: 6-min walk test.
