# Supplementary material for: Effects of new hypoglycemic drugs on cardiac remodeling: a systematic review and network meta-analysis
Source: BMC Cardiovasc Disord. 2023 Jun 9;23:293. doi: 10.1186/s12872-023-03324-6 (PMC10251583; doi:10.1186/s12872-023-03324-6)
Supplement: Supplementary file 9 — Additional file 9: Table S3.Matrix of pairwise comparisons of regmins on change of LVEF% in subgroup of patients with CVD alone.Matrix of pairwise comparisons of regimens on change of LVEF% in subgroup of patients with CVD and T2DM.Matrix of pairwise comparisons of regimens on change of E/e’ in subgroup of patients with CVD and T2DM.Matrix of pairwise comparisons of regimens on change of LVESV in subgroup of patients with CVD and T2DM.Matrix of pairwise comparisons of regimens on change of LVEDV in subgroup of patients with CVD and T2DM.Matrix of pairwise comparisons of regimens on change of SBP in subgroup of patients with CVD and T2DM.Matrix of pairwise comparisons of regmins on change of NT-pro BNP in subgroup of patients with CVD alone.Matrix of pairwise comparisons of regmins on change of 6MWT in subgroup of patients with CVD alone.Matrix of pairwise comparisons of regimens on change of e' in overall population.Matrix of pairwise comparisons of regimens on change of E/e' in overall population.Matrix of pairwise comparisons of regimens on change of E/A in overall population.Matrix of pairwise comparisons of regimens on change of SBP in overall population.Matrix of pairwise comparisons of regimens on change of NT-pro BNP in overall population.Matrix of pairwise comparisons of regimens on change of 6MWT in overall population. [file 12872_2023_3324_MOESM9_ESM.pdf]

TableS3 League table

TableS3(a) Matrix of pairwise comparisons of regmins on change of LVEF% in subgroup of patients with CVD alone (shown as standard mean difference and 95% confidence intervals).

	A	B	C
SCURA(%)			
A	0	-1.65 (-2.81,-0.49)	-0.73 (-2.23,0.77)
B Placebo	1.65 (0.49,2.81)	0	0.92 (-0.02,1.86)
C	0.73 (-0.77,2.23)	-0.92 (-1.86,0.02)	0

Abbre：A GLP-1RA, B Placebo, C SGLT-2i

TableS3(b) Matrix of pairwise comparisons of regimens on change of LVEF% in subgroup of patients with CVD and T2DM (shown as standard mean difference and 95% confidence intervals).

	B	D	A	C
SCURA(%)				
B	0	-0.25 (-0.85,0.34)	-0.43 (-1.14,0.28)	-0.42 (-0.89,0.06)
D	0.25 (-0.34,0.85)	0	-0.07 (-0.87,0.72)	-0.16 (-0.92,0.60)
A	0.43 (-0.28,1.14)	0.07 (-0.72,0.87)	0	0.01 (-0.51,0.54)
C Placebo	0.42 (-0.06,0.89)	0.16 (-0.60,0.92)	-0.01 (-0.54,0.51)	0

Abbre：A DDP-4i, B GLP-1RA, C Placebo, D SGLT-2i

TableS3(c) Matrix of pairwise comparisons of regimens on change of E/e’ in subgroup of patients with CVD and T2DM (shown as standard mean difference and 95% confidence intervals).

	D	A	B	C
SCURA(%)				
D	0	0.01 (-0.60,0.62)	0.53 (-0.05,1.11)	0.43 (0.06,0.79)
A	-0.01 (-0.62,0.60)	0	0.52 (-0.32,1.36)	0.42 (-0.29,1.13)
B	-0.53 (-1.11,0.05)	-0.52 (-1.36,0.32)	0	-0.10 (-0.55,0.35)
C Placebo	-0.43 (-0.79,-0.06)	-0.42 (-1.13,0.29)	0.10 (-0.35,0.55)	0

Abbre： A DDP-4i, B GLP-1RA, C Placebo, D SGLT-2i

Table S3(d) Matrix of pairwise comparisons of regimens on change of LVESV in subgroup of patients with CVD and T2DM (shown as standard mean difference and 95% confidence intervals).

	A	C	B
SCURA(%)			
A	0	0.15 (-0.17,0.47)	0.33 (0.04,0.61)
C	-0.15 (-0.47,0.17)	0	0.18 (-0.02,0.37)
B Placebo	-0.33 (-0.61,-0.04)	-0.18 (-0.37,0.02)	0

Abbre： A GLP-1RA, B Placebo, C SGLT-2i

Table S3(e) Matrix of pairwise comparisons of regimens on change of LVEDV in subgroup of patients with CVD and T2DM (shown as standard mean difference and 95% confidence intervals).

	B	D	C	A
SCURA(%)				
B	0	0.09 (-0.33,0.52)	0.23 (-0.12,0.59)	0.37 (-0.24,0.99)
D	-0.09 (-0.52,0.33)	0	0.14 (-0.09,0.37)	0.28 (-0.27,0.83)
C Placebo	-0.23 (-0.59,0.12)	-0.14 (-0.37,0.09)	0	0.14 (-0.36,0.64)
A	-0.37 (-0.99,0.24)	-0.28 (-0.83,0.27)	-0.14 (-0.64,0.36)	0

Abbre： A DDP-4i, B GLP-1RA, C Placebo, D SGLT-2i

Table S3(f) Matrix of pairwise comparisons of regimens on change of SBP in subgroup of patients with CVD and T2DM (shown as standard mean difference and 95% confidence intervals).

	A	D	B	C
SCURA(%)				
A	0	0.93 (0.44,1.43)	0.64 (0.03,1.25)	-0.24 (-1.04,0.57)
D	-0.93 (-1.43,-0.44)	0	0.12 (-0.32,0.56)	0.41 (0.15,0.67)
B	-0.64 (-1.25,-0.03)	-0.12 (-0.56,0.32)	0	0.29 (-0.22,0.80)
C Placebo	0.24 (-0.57,1.04)	-0.41 (-0.67,-0.15)	-0.29 (-0.80,0.22)	0

Abbre： A DDP-4i, B GLP-1RA, C Placebo, D SGLT-2i

Table S3(g) Matrix of pairwise comparisons of regmins on change of NT-pro BNP in subgroup of patients with CVD alone (shown as standard mean difference and 95% confidence intervals).

	A	B	C
SCURA(%)			
A	0	0.30 (-0.17,0.77)	0.54 (-0.08,1.17)
B Placebo	-0.30 (-0.77,0.17)	0	0.25 (-0.16,0.65)
C	-0.54 (-1.17,0.08)	-0.25 (-0.65,0.16)	0

Abbre： A GLP-1RA, B Placebo, C SGLT-2i

Table S3(h) Matrix of pairwise comparisons of regmins on change of 6MWT in subgroup of patients with CVD alone (shown as standard mean difference and 95% confidence intervals).

	A	C	B
SCURA(%)			
A	0	-0.86 (-2.68,0.97)	-1.31 (-2.95,0.33)
C	0.86 (-0.97,2.68)	0	-0.45 (-1.26,0.35)
B Placebo	1.31 (-0.33,2.95)	0.45 (-0.35,1.26)	0

Abbre： A = GLP-1RA, B = Placebo, C = SGLT-2i

TableS3(i) Matrix of pairwise comparisons of regimens on change of e' in overall population (shown as standard mean difference and 95% confidence intervals).

	A	D	C	B
SCURA(%)				
A	0	-2.96 (-4.24,-1.68)	-3.82 (-4.72,-2.92)	-4.24 (-5.22,-3.27)
D	2.96 (1.68,4.24)	0	-0.86 (-1.77,0.05)	-1.28 (-2.27,-0.30)
C Placebo	3.82 (2.92,4.72)	0.86 (-0.05,1.77)	0	-0.43 (-0.81,-0.04)
B	4.24 (3.27,5.22)	1.28 (0.30,2.27)	0.43 (0.04,0.81)	0

Abbre： A DDP-4i, B GLP-1RA, C Placebo, D SGLT-2i

Table S3(j) Matrix of pairwise comparisons of regimens on change of E/e' in overall population (shown as standard mean difference and 95% confidence intervals).

	A	D	B	C
SCURA(%)				
A	0	5.56 (-2.48,13.60)	5.78 (0.60,10.95)	5.97 (1.59,10.35)
D	-5.56 (-13.60,2.48)	0	6.07 (-2.05,14.19)	6.26 (-1.38,13.90)
B	-5.78 (-10.95,-0.60)	-6.07 (-14.19,2.05)	0	0.19 (-2.60,2.98)
C Placebo	-5.97 (-10.35,-1.59)	-6.26 (-13.90,1.38)	-0.19 (-2.98,2.60)	0

Abbre： A DDP-4i, B GLP-1RA, C Placebo, D SGLT-2i

Table S3(k) Matrix of pairwise comparisons of regimens on change of E/A in overall population (shown as standard mean difference and 95% confidence intervals).

	A	D	C	B
SCURA(%)				
A	0	-2.97 (-6.92,0.98)	-1.84 (-3.96,0.28)	-2.57 (-5.17,0.04)
D	2.97 (-0.98,6.92)	0	-2.06 (-5.77,1.65)	-2.78 (-6.79,1.22)
C Placebo	1.84 (-0.28,3.96)	2.06 (-1.65,5.77)	0	-0.73 (-2.24,0.79)
B	2.57 (-0.04,5.17)	2.78 (-1.22,6.79)	0.73 (-0.79,2.24)	0

Abbre： A DDP-4i, B GLP-1RA, C Placebo, D SGLT-2i

Table S3(l) Matrix of pairwise comparisons of regimens on change of SBP in overall population (shown as standard mean difference and 95% confidence intervals).

	A	D	B	C
SCURA(%)				
A	0	0.00 (-0.33,0.33)	0.46 (-0.20,1.13)	0.40 (-0.13,0.93)
D	-0.00 (-0.33,0.33)	0	-0.28 (-1.06,0.49)	0.12 (-0.34,0.58)
B	-0.46 (-1.13,0.20)	0.28 (-0.49,1.06)	0	0.40 (-0.22,1.02)
C Placebo	-0.40 (-0.93,0.13)	-0.12 (-0.58,0.34)	-0.40 (-1.02,0.22)	0

Abbre： A DDP-4i, B GLP-1RA, C Placebo, D SGLT-2i

TableS3(m) Matrix of pairwise comparisons of regimens on change of NT-pro BNP in overall population (shown as standard mean difference and 95% confidence intervals).

	D	B	C	A
SCURA(%)				
D	0	-0.14 (-0.94,0.66)	-0.14 (-0.87,0.60)	0.28 (-0.49,1.04)
B	0.14 (-0.66,0.94)	0	0.00 (-0.31,0.32)	0.14 (-0.43,0.71)
C Placebo	0.14 (-0.60,0.87)	-0.00 (-0.32,0.31)	0	0.14 (-0.33,0.62)
A	-0.28 (-1.04,0.49)	-0.14 (-0.71,0.43)	-0.14 (-0.62,0.33)	0

Abbre： A DDP-4i, B GLP-1RA, C Placebo, D SGLT-2i

TableS3(n) Matrix of pairwise comparisons of regimens on change of 6MWT in overall population (shown as standard mean difference and 95% confidence intervals).

	A	C	B
SCURA(%)			
A	0	-1.18 (-2.74,0.38)	-1.52 (-2.76,-0.29)
C	1.18 (-0.38,2.74)	0	-0.34 (-1.30,0.61)
B Placebo	1.52 (0.29,2.76)	0.34 (-0.61,1.30)	0

Abbre： A GLP-1RA, B Placebo, C SGLT-2i

Note: Abbre: abbreviations; **e'**: early diastolic velocity; **E/e'**: mitral inflow E velocity to tissue doppler e' ratio; **E/A**: early diastolic to late diastolic velocities ratio**; CVD**: cardiovascular disease; **DPP-4i**: dipeptidyl peptidase-4 inhibitor; **GLP-1RA**: glucagon-like peptide-1 receptor agonist; **LVEDD**: left ventricular end-diastolic diameter; **LVEDV**: LV end-diastolic volume; **LVEF**: LV ejection fraction; **LVESD**: LV end-systolic diameter; **LVESV**: LV end-systolic volume; **LVMI**: LV mass index; **NT-pro BNP**: immunoreactive amino-terminal pro-brain natriuretic peptide; **SBP**: systolic blood pressure; **SGLT-2i**: sodium glucose cotransporter type 2 inhibitor; **T2DM**: type 2 diabetes; **6MWT**: 6-min walk test.
