# Supplementary material for: Effects of new hypoglycemic drugs on cardiac remodeling: a systematic review and network meta-analysis
Source: BMC Cardiovasc Disord. 2023 Jun 9;23:293. doi: 10.1186/s12872-023-03324-6 (PMC10251583; doi:10.1186/s12872-023-03324-6)
Supplement: Supplementary file 10 — Additional file 10. [file 12872_2023_3324_MOESM10_ESM.pdf]

SBP

(1) Evidence Network. 14 pieces of literature reported SBP involving 3 antidiabetic regimens. The dot size indicates the sample size using the intervention, the line thickness represents the number of RCTs using the two-point treatment intervention, forming three closed-loop formation. The network evidence of SBP is exhibited in Additional fle 4 Figure S3(j).

(2) Network Meta-analysis. The results showed that no significant difference was identified between the 4 interventions. Results are shown in Additional fle 9: TableS3(l) and Additional fle 6: FigureS5(j).

Results of subgroup analyses

In the subgroup analysis, SBP was reported in 5 literatures that included patients with T2DM combined with CVD, involving 3 hypoglycemic drugs. The network comparison was conducted among the 3 hypoglycemic drugs, resulting in a total of 12 pair-wise comparisons. Compared with placebo, the MD and 95% CI of SGLT-2i in the treatment of SBP were -0.41mmHg and (-0.67,-0.15), respectively. The detailed results are shown in Additional fle 9: TableS3(f).

NT-pro BNP

(1) Evidence Network. NT-pro BNP was reported in 11 studies, involving 3 antidiabetic therapies. The point size represents the sample size of the intervention, the line thickness represents the number of RCTs using two-point treatment intervention, and all interventions represent direct comparison without closed-loop formation. The network evidence of NT-pro BNP is shown in Additional fle 4 Figure S3(k).

(2) Network Meta-analysis. There was no difference in mean change in NT-pro BNP between each of the 3 drugs and placebo or in pairwise comparison between any two of the 3 drugs in treatment effect. (Additional fle 9: TableS3(m) and Additional fle 6: FigureS5(k)).

Results of subgroup analyses

In subgroup analysis, 7 literatures reported NT-pro BNP in patients with CVD disease only. The network comparison was carried out in two hypoglycemic drugs, resulting in a total of six paired comparisons. No statistically significant result was found in mean change in NT-pro BNP between each of the 3 drugs and placebo or in pairwise comparison between any two of the 3 drugs in treatment effect (Additional fle 9: TableS3(g)).

6MWT

(1) Evidence Network. 6MWT was reported in 15 studies, involving 2 antidiabetic therapies (GLP-1RA and SGLT-2i). All interventions represent direct comparison without closed-loop formation. The network evidence of 6-min walk is shown in Additional fle 4 Figure S3(l).

(2) Network Meta-analysis. After pooling 15 studies, a significant association

between GLP-1RA therapy and improvement of 6-min walk distance was found in the overall population compared with placebo [MD=1.52m 95%CI (0.29, 2.76)]. No statistically significant result was found in mean change in NT-proBNP between SGLT-2i and placebo or in pairwise comparison between the two 2 drugs in treatment effect (Additional fle 9: TableS3(n) and Additional fle 6: FigureS5(l)).

Results of subgroup analyses

In subgroup analysis, 4 literatures reported 6MWT in patients with CVD alone. Network comparison was conducted among two hypoglycemic drugs, resulting in a total of 6 pairwise comparisons without statistical significance. The specific results are shown in Additional fle 9: TableS3(h).

Note: CVD: cardiovascular disease; **DPP-4i**: dipeptidyl peptidase-4 inhibitor; **GLP-1RA**: glucagon-like peptide-1 receptor agonist; **NT-pro BNP**: immunoreactive amino-terminal pro-brain natriuretic peptide; **SBP**: systolic blood pressure; **SGLT-2i**: sodium glucose cotransporter type 2 inhibitor; **T2DM**: type 2 diabetes; **6MWT**: 6-min walk test.
